# Intrinsic B cell TLR-BCR linked coengagement induces class-switched, hypermutated, neutralizing antibody responses in absence of T cells

**DOI:** 10.1126/sciadv.ade8928

**Published:** 2023-04-28

**Authors:** Carlos E. Rivera, Yulai Zhou, Daniel P. Chupp, Hui Yan, Amanda D. Fisher, Raphael Simon, Hong Zan, Zhenming Xu, Paolo Casali

**Affiliations:** ^1^ Department of Microbiology, Immunology & Molecular Genetics, University of Texas Long School of Medicine, UT Health Science Center, San Antonio, TX 78229, USA.; ^2^ Center for Vaccine Development, University of Maryland School of Medicine, Baltimore, MD 21201, USA.; ^3^ Department of Medicine, University of Texas Long School of Medicine, UT Health Science Center, San Antonio, TX 78229, USA.

## Abstract

Maturation of antibody responses entails somatic hypermutation (SHM), class-switch DNA recombination (CSR), plasma cell differentiation, and generation of memory B cells, and it is thought to require T cell help. We showed that B cell Toll-like receptor 4 (TLR4)–B cell receptor (BCR) (receptor for antigen) coengagement by 4-hydroxy-3-nitrophenyl acetyl (NP)–lipopolysaccharide (LPS) (*Escherichia coli* lipid A polysaccharide O-antigen) or TLR5-BCR coengagement by *Salmonella* flagellin induces mature antibody responses to NP and flagellin in *Tcr*β*^−/−^Tcr*δ*^−/−^* and NSG/B mice. TLR-BCR coengagement required linkage of TLR and BCR ligands, “linked coengagement.” This induced B cell CSR/SHM, germinal center–like differentiation, clonal expansion, intraconal diversification, plasma cell differentiation, and an anamnestic antibody response. In *Tcr*β*^−/−^Tcr*δ*^−/−^* mice, linked coengagement of TLR4-BCR by LPS or TLR5-BCR by flagellin induced protective antibodies against *E. coli* or *Salmonella* Typhimurium. Our findings unveiled a critical role of B cell TLRs in inducing neutralizing antibody responses, including those to microbial pathogens, without T cell help.

## Teaser

B cell linked coengagement of TLR-BCR induces class-switched, hypermutated, neutralizing antibody responses in absence of T cells.

## INTRODUCTION

The maturation of an antibody response entails production of class-switched and somatically mutated immunoglobulin G (IgG), IgE, and/or IgA that, hence, are effective in neutralizing invading microbial pathogens, such as viruses and bacteria, in killing tumor cells or in damaging tissues and organs (autoantibodies). B cell somatic hypermutation (SHM) and class-switch DNA recombination (CSR) are initiated by activation-induced cytidine deaminase (AID), which introduces nicks and/or double-stranded breaks in immunoglobulin (Ig) loci (*1*). SHM inserts mainly point mutations into rearranged Ig variable V(D)J DNA regions, thereby providing the structural substrate for positive selection of higher-affinity antibody submutants by the antigen driving the response (*1*). CSR replaces the constant heavy chain (C_H_) region, generally Cμ (IgM) with Cδ, Cγ, Cε, or Cα (IgD, IgG, IgE, or IgA), thereby diversifying the antibody biological effector functions (*2*–*4*). Later in the antibody response, B cells bearing class-switched and somatically mutated receptors [B cell receptor (BCR)] with a high affinity for antigen differentiate to plasma cells and memory B cells. Plasma cells are terminally differentiated elements that secrete massive amounts of antibodies (*5*, *6*). Memory B cells are quiescent elements, which upon reactivation by the antigen that induced their generation, respond by differentiating to plasma cells or by undergoing further round(s) of SHM and affinity maturation (*7*–*9*).

It has long been held that the generation of mature class-switched and somatically mutated antibody responses requires, in addition to B cell BCR engagement by antigen, the intervention of activated T helper cells (*10*). These T cells express surface CD154 (CD40 ligand) that engages CD40 constitutively expressed on B cells. Upon BCR engagement by antigen and in the presence of cytokines, such as interleukin-4 (IL-4), IL-6, IL-21, transforming growth factor–β (TGF-β), or interferon-γ (IFN-γ), CD154:CD40 engagement activates B cells to undergo SHM/CSR and differentiation to plasma cells or memory B cells, generally within the specialized environment of germinal centers (GCs) (*11*, *12*). Therefore, B cells bearing BCR mutants with higher affinity for the antigen driving the response are positively selected and clonally expanded (*13*, *14*). By contrast, in T-independent responses, T cell CD154:B cell CD40 engagement is substituted by surface or intracellular B cell Toll-like receptor (TLR) engagement by TLR ligands (*10*, *15*). T-independent antigenic stimuli have been generally associated with failure to induce specific mature antibody responses entailing SHM/CSR, GC formation, and generation of memory B cells (*8*, *11*, *16*), although “memory”-like B cells with a peculiar phenotype and making only IgM are induced by T-independent type II 4-hydroxy-3-nitrophenyl acetyl (NP)–Ficoll (*17*, *18*).

T cell–deficient mice, however, have been shown to make IgG antibodies in response to live polyoma virus (*19*), palmitoylated human β-amyloid (Aβ) peptide–lipid A liposomes (*20*) and bacterial phage Qβ-derived virus-like particles (*21*). Nevertheless, these reports of antibody responses in the absence of T cells provided no or scant information on SHM/CSR, plasmablast and plasma cell differentiation, potential antibody neutralizing activity, generation of memory B cells, or anamnestic response and underlying mechanisms, with the response to Aβ liposomes being the only one to suggest a TLR as a possible mediator of CSR induction (*20*). As we and others, however, have shown using a well-controlled in vitro B cell culture platform, TLR or BCR engagement alone induces only marginal, no AID, or Blimp-1 expression, leading to abortive CSR or plasma cell differentiation (*22*–*25*). As we have also shown, TLR engagement, by ligands of surface TLR1/2 or TLR4 or endosomal TLR7 or TLR9, synergizes with BCR engagement to induce AID and Blimp-1 expression, leading to CSR and plasma cell differentiation, as efficiently as CD154:CD40 engagement does (*24*, *25*). This concomitant TLR and BCR coengagement, however, would not be sufficient for induction of fully mature (i.e., including SHM and generation of memory B cells) and neutralizing T-independent antibody responses (*19*–*21*). We argue here that induction of these responses requires physical linkage of the molecule(s) that simultaneously engage TLR and BCR (“TLR-BCR linked coengagement”). This B cell TLR-BCR linked coengagement would synergize to induce T-independent, class-switched, somatically mutated, high-affinity antibody responses entailing GC-like B cell and plasma cell differentiation as well as generation of memory B cells.

We assessed the ability of B cell TLR4-BCR and TLR5-BCR coengagement to induce a specific mature antibody response, including SHM, CSR, B cell clonal expansion, intraclonal diversification, plasma cell differentiation, and generation of memory B cells by injecting *Tcr*β*^−/−^Tcr*δ*^−/−^* mice (devoid of T cells) with NP conjugated to *Escherichia coli* lipopolysaccharides (NP-LPS), which coengages TLR4 and NP-cognate BCR, or *Salmonella* (*S.*) Typhimurium flagellin, which coengages TLR5 and flagellin-cognate BCR, and analyzed the antibody responses to NP and flagellin. Induction of these responses requires physical linkage of TLR and BCR ligands, as we established by using NP-LPS, NP-Ficoll admixed with LPS, or LPS alone in vivo and in vitro Vaccination of *Tcr*β*^−/−^Tcr*δ*^−/−^* mice with *E. coli* LPS, which coengages B cell TLR4 by its lipid A moiety and cognate BCR by its polysaccharidic O-antigen moiety, and *S.* Typhimurium flagellin induced neutralizing and protective antibody responses against *E. coli* and against *S.* Typhimurium. A similar TLR4-BCR and TLR5-BCR linked coengagement in nonobese diabetic/severe combined immunodeficiency (NOD) *scid* gamma (NSG)/B mice (immunodeficient NSG mice lack not only T cells but also all other immune elements and are grafted with purified C57BL/6 B cells) induced class-switched, high-affinity antibodies to NP and flagellin. Last, analysis of surface TLR4 and TLR5 expression by single-cell flow imaging showed that these two TLRs are present on mouse and human naïve B cells and are both up-regulated upon TLR-BCR linked coengagement, leading to CSR and plasma cell differentiation. Intrinsic B cell TLR-BCR linked coengagement likely plays critical roles in antibody responses to T-independent antigens, particularly microbial components. In addition, it would play an important role in early stages of responses to select T-dependent antigens when T cell help is not yet available and potentiate the late-stage response by adding to T cell help. It likely represents an evolutionary conserved mechanism, one that may inform the development of TLR-based vaccines, particularly important for subjects with an immature or declining T cell compartment, such as the infant and the elderly.

## RESULTS

### TLR4-BCR coengagement induces a specific, class-switched response in *Tcr*β*^−/−^Tcr*δ*^−/−^* mice

**Fig. 1.**
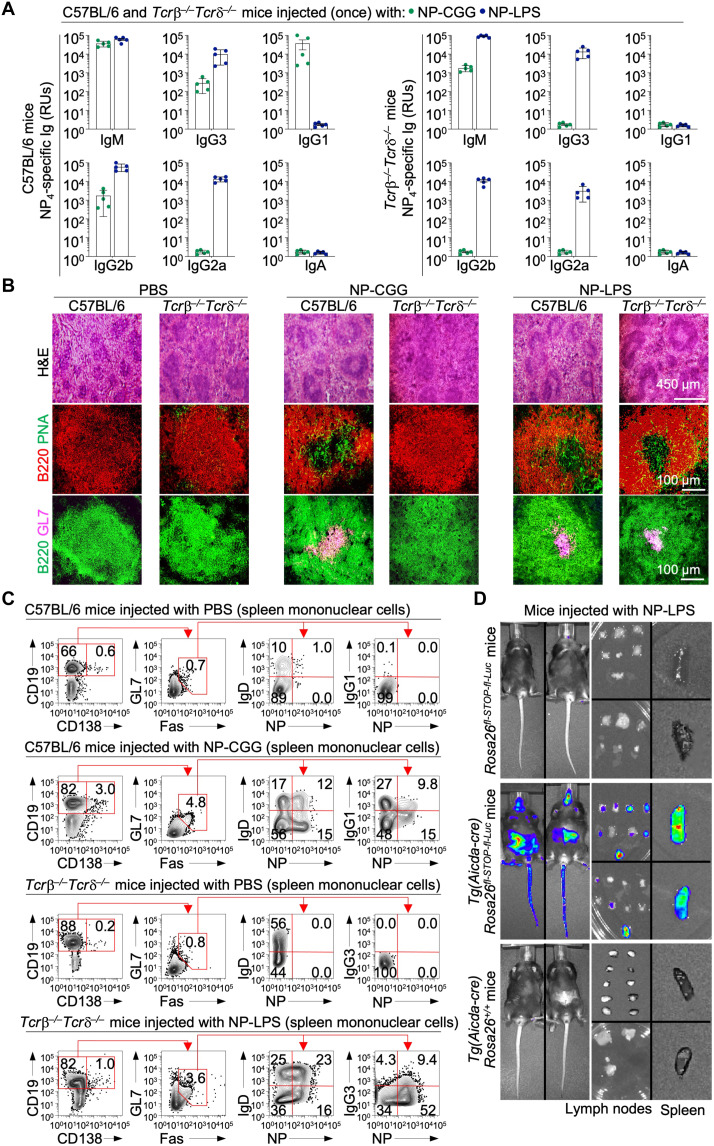
NP-LPS induces GC-like structures and GC-like B cell differentiation in *Tcr*β*^−/−^Tcr*δ*^−/−^* mice. (**A**) C57BL/6 and *Tcr*β*^−/−^Tcr*δ*^−/−^* mice were injected intraperitoneally with T-independent NP-LPS (in PBS) or T-dependent NP-CGG (in PBS) (*n* = 5 mice per group). Sera were collected 14 days after injection and analyzed for NP_4_-specific IgM, IgG3, IgG1, IgG2b, IgG2a, and IgA by enzyme-linked immunosorbent assay (ELISA). Antibody titers are expressed as relative units (RUs). Histograms depict mean values ± SEM. Each dot represents an individual mouse. Ig, immunoglobulin. (**B**) C57BL/6 and *Tcr*β*^−/−^Tcr*δ*^−/−^* mice were injected intraperitoneally with PBS, NP-CGG, or NP-LPS (*n* = 3 mice per group; data are from one representative mouse of each group) and euthanized 10 days later. Spleens were analyzed for GC-like formations by hematoxylin and eosin (H&E) (scale bar, 400 μm) and immunofluorescence staining (scale bar, 100 μm). Frozen spleen sections were costained with fluorescein isothiocyanate (FITC)–anti-B220 and phycoerythrin (PE)–anti-GL7 monoclonal antibodies (mAbs) or Alexa Fluor 488–conjugated PNA and phycoerythrin (PE)–anti-B220 mAb. (**C**) C57BL/6 mice were injected intraperitoneally with PBS or NP-CGG; *Tcr*β*^−/−^Tcr*δ*^−/−^* mice were injected intraperitoneally with PBS or NP-LPS. Mice were euthanized 14 days later (*n* = 4 mice per group). Total B cells, GC-like B cells, and plasmablasts/plasma cells were identified in spleens by anti-CD19, anti-GL7, anti-CD95 (Fas), and anti-CD138 mAbs. NP-specific GL7^+^Fas^+^ IgD^+^, NP-specific GL7^+^Fas^+^ IgG1^+^, and NP-specific GL7^+^Fas^+^ IgG3^+^ B cells were identified by NP-PE, anti-IgD, anti-IgG1, and anti-IgG3 mAbs, as analyzed by flow cytometry (numbers are percentages of total mononuclear cells analyzed). (**D**) *Rosa26^fl-STOP-fl-Luc^*, *Tg(Aicda-cre)Rosa26^fl-STOP-fl-Luc^*, and *Tg(Aicda-cre)Rosa26^+/+^* mice (*n* = 2 mice per genotype) were injected intraperitoneally with NP-LPS and euthanized 14 days later. AID-expressing B cells were identified in the spleens, lymph nodes, and splanchnic district by luciferase expression using bioluminescence imaging.

To formally prove the T cell independence of the LPS-induced antibody response, as suggested by data in vitro and in vivo (*24*–*27*), we injected *Tcr*β*^−/−^Tcr*δ*^−/−^* (T cell deficient; fig. S1A) and C57BL/6 mice (controls) with NP-LPS in phosphate-buffered saline (PBS). NP-LPS–injected *Tcr*β*^−/−^Tcr*δ*^−/−^* mice made specific NP_4_-binding IgG3, IgG2b, and IgG2a but not IgG1 antibodies—NP_4_ binding reflects an antibody Fab high affinity for NP (Fig. 1A) (*28*, *29*). These antibodies were produced by NP_4_-specific IgG3-, IgG2b-, and IgG2a-secreting cells [antibody-secreting cells (ASCs); i.e., plasmablasts and plasma cells)] at levels comparable to those made by C57BL/6 mouse controls, albeit with marginally lower IgG2b and IgG2a; in C57BL/6 mice, IgG2b and IgG2a production was likely boosted by TGF-β and IFN-γ (*2*, *30*), as secreted by T cells (Fig. 1A and fig. S2). In *Tcr*β*^−/−^Tcr*δ*^−/−^* mice, NP-LPS induced NP-specific class-switched IgG3^+^, IgG2b^+^, and IgG2a^+^ B cells to an extent comparable to that in C57BL/6 mice (figs. S1, B and C and S3). NP-LPS–induced class-switched B cells expressed germline Iγ3-Cγ3, Iγ2b-Cγ2b, and Iγ2a-Cγ2a transcripts; *Aicda* and *Prdm1*; and post-recombination Iμ-Cγ3, Iμ-Cγ2b, and Iμ-Cγ2a transcripts (fig. S1D). The failure of *Tcr*β*^−/−^Tcr*δ*^−/−^* mice to make anti-NP IgG3, IgG1, IgG2b, and IgG2a antibodies in response to (T-dependent) NP conjugated with chicken γ-globulins (NP-CGG in PBS) reflected the lack of T cell help in these mice. As expected, both *Tcr*β*^−/−^Tcr*δ*^−/−^* and C57BL/6 mice made marginal amounts of NP-specific IgA in response to NP-LPS or NP-CGG, and control *Aicda^−/−^* mice injected with NP-LPS made no NP- specific IgG3^+^, IgG1^+^, or IgG2b^+^ B cells (fig. S1, E and F).

Consistent with the failure to mount NP-specific IgG1 antibodies in response to NP-CGG and unlike C57BL/6 mice, NP-CGG–injected *Tcr*β*^−/−^Tcr*δ*^−/−^* mice did not develop GC-like structures (Fig. 1B). By contrast and consistent with their making of NP-specific IgG3 and IgG2b, NP-LPS–injected *Tcr*β*^−/−^Tcr*δ*^−/−^* mice developed spleen GC-like structures, as NP-LPS–injected C57BL/6 mice did. These GC-like structures were evocative of GC-like formations in C57BL/6 mice immunized with T-dependent NP-CGG (Fig. 1B). In *Tcr*β*^−/−^Tcr*δ*^−/−^* mice, production of IgG to NP and emergence of GC-like structures included peanut agglutinin–positive (PNA^+^) and GL7^+^ B cells, as similarly elicited in C57BL/6 mice by NP-LPS (Fig. 1B). As in C57BL/6 mice injected with T-dependent NP-CGG, GC-like structures in NP-LPS–injected *Tcr*β*^−/−^Tcr*δ*^−/−^* mice included NP-specific GL7^+^Fas^+^ GC-like B cells and CD19^+^CD138^+^ plasmablasts (Fig. 1C and figs. S1G and S4, A and B). Further supporting the B cell TLR4-BCR coengagement-mediated GC-like B cell differentiation, GC-like formation, and generation of NP-specific class-switched antibodies, NP-LPS induced high levels of AID expression, as shown in the lymph nodes and spleen of *Tg(Aicda-cre)Rosa26^fl-STOP-fl-Luc^* mice but not in control *Rosa26^fl-STOP-fl-Luc^* and *Tg(Aicda-cre)Rosa26^+/+^* mice (Fig. 1D). The lower levels of NP-LPS–induced IgG3^+^ B cells and NP-specific IgG3 antibodies in mixed bone marrow μMT/*Tlr4^−/−^* chimeric mice (constructed by grafting irradiated C57BL/6 mice with bone marrow cells from B cell–deficient μMT mice and *Tlr4^−/−^* mice) than in μMT/*Tlr4^+/+^*chimeric controls emphasized the critical role of B cell TLR4 in the T-independent NP-LPS antibody response (fig. S5)—the residual IgG3^+^ B cells in NP-LPS–injected μMT/*Tlr4^−/−^* mice were possibly made by some “leaked” B cells from μMT donor bone marrow.

Thus, B cell TLR4-BCR coengagement supports a class-switched, high-affinity antibody response involving GC-like B cell differentiation, formation of GC-like structures, and generation of plasma cells in the absence of T cells.

### TLR4-BCR coengagement requires physical linkage of TLR and BCR ligands (linked coengagement) for induction of a specific and class-switched antibody response

**Fig. 2.**
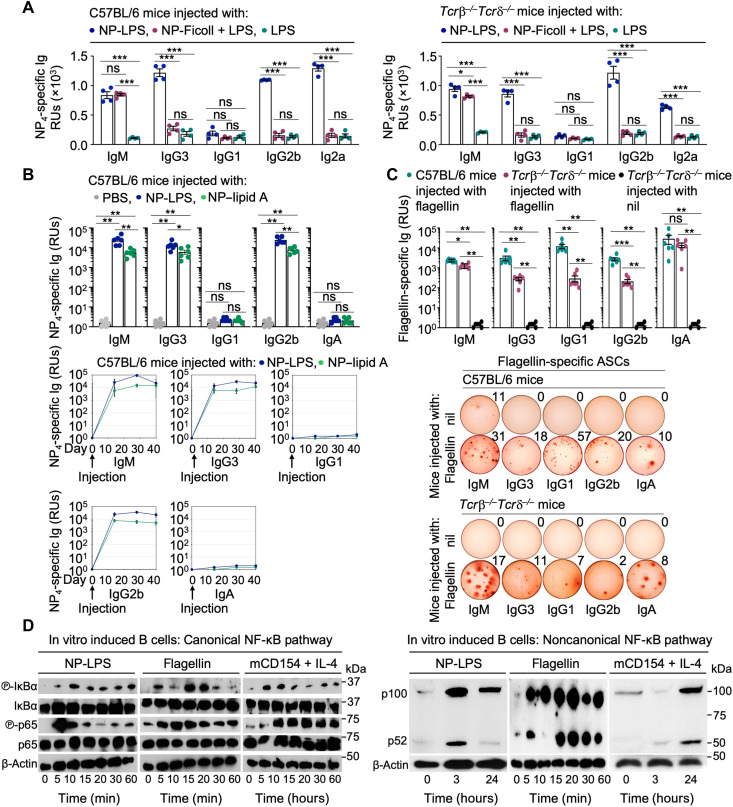
TLR-BCR coengagement requires physical linkage of B cell TLR and BCR ligands for elicitation of T-independent class-switched antibody responses and activates the canonical and noncanonical NF-κB pathways. (**A**) C57BL/6 and *Tcr*β*^−/−^Tcr*δ*^−/−^* mice were injected intraperitoneally with NP-LPS, NP-Ficoll admixed with LPS, or LPS alone (*n* = 4 mice per group). Sera were collected 42 days after injection and analyzed for NP_4_-specific IgM, IgG3, IgG1, IgG2b, and IgG2a by ELISA (titers expressed as RUs). Histograms depict mean values ± SEM. Each dot represents an individual mouse. (**B**) C57BL/6 mice were injected intraperitoneally with PBS, NP–lipid A, or NP-LPS (*n* = 6 mice per group). Sera were collected on days 0, 14, 28, and 40 and analyzed for NP_4_-specific IgM, IgG3, IgG1, IgG2b, and IgA by ELISA (titers expressed as RUs). Histograms depict mean NP_4_-specific antibody titers ± SEM at day 14. Each dot represents an individual mouse. Graphs depict NP_4_-specific antibody levels through day 40 (titers expressed as RUs). Data points are mean values ± SEM of six mice at different times. (**C**) C57BL/6 and *Tcr*β*^−/−^Tcr*δ*^−/−^* mice were injected intraperitoneally with *S.* Typhimurium flagellin (in alum) or nil (alum) and euthanized 14 days later (*n* = 6 mice per group). Sera were analyzed for flagellin-specific IgM, IgG3, IgG1, IgG2b, and IgA by ELISA (titers expressed as RUs). Histograms depict mean values ± SEM. Each dot represents an individual mouse. Spleens were analyzed for flagellin-specific IgM, IgG3, IgG1, IgG2b, and IgA ASCs by enzyme-linked immunosorbent spot (ELISPOT), and numbers depict ASCs per 10^5^ plated cells. (**D**) Induction of the canonical and noncanonical NF-κB pathway in *Tcr*β*^−/−^Tcr*δ*^−/−^* B cells stimulated in vitro with NP-LPS, *S.* Typhimurium flagellin, or mCD154 plus IL-4 for indicated times. *P < 0.05, **P < 0.01, and ***P < 0.001 (unpaired *t* test).

To address whether induction of a mature antibody response to NP by NP-LPS in the absence of T cells required physical linkage of TLR and BCR ligands, we injected *Tcr*β*^−/−^Tcr*δ*^−/−^* and C57BL/6 mice with NP-LPS, NP-Ficoll admixed with LPS, or LPS alone. In *Tcr*β*^−/−^Tcr*δ*^−/−^* mice, NP-LPS, but not NP-Ficoll admixed with LPS or LPS alone, induced NP-specific high-affinity IgG3, IgG2b, and IgG2a and did so at levels comparable to those in C57BL/6 mouse controls; as expected, neither C57BL/6 nor *Tcr*β*^−/−^Tcr*δ*^−/−^* mice made detectable levels of IgG1 antibodies to NP (Fig. 2A). Further proof for requirement of physical linkage of TLR4 and BCR ligands for TLR4-BCR coengagement was provided in vitro. Purified C57BL/6 B cells stimulated with NP-LPS, but not LPS admixed with NP-Ficoll, differentiated to NP-specific class-switched IgG3^+^ B cells (fig. S6A). Last, the critical role of LPS lipid A moiety in TLR4-BCR coengagement was verified by conjugating lipid A purified from *E. coli* LPS with NP (NP–lipid A). NP–lipid A injection of C57BL/6 mice induced a NP-specific high-affinity IgG3 and IgG2b antibody response comparable in magnitude to that induced by NP-LPS in similar mouse controls (Fig. 2B). Thus, the induction of a specific high-affinity and class-switched antibody response by TLR4-BCR coengagement requires physical linkage of the TLR and BCR ligands (“linked coengagement”).

### Induction of a mature antibody response by TLR-BCR linked coengagement extends to TLR5

To determine whether induction of a T-independent class-switched antibody response by TLR-BCR coengagement extended beyond TLR4, we injected *Tcr*β*^−/−^Tcr*δ*^−/−^* and C57BL/6 mice with purified *S.* Typhimurium flagellin (in alum). This is a 50-kDa protein that “absolutely” requires T cell help for induction of a specific antibody response (*31*–*33*). Flagellin, however, also efficiently binds TLR5 through both its NH_2_- and COOH ends (*34*–*36*). Hence, it could inherently satisfy the TLR-BCR ligands’ linkage requirement. Flagellin injection in *Tcr*β*^−/−^Tcr*δ*^−/−^* mice induced specific IgG3, IgG2b, and IgA antibodies, as secreted by flagellin-specific ASCs, at levels comparable to those of their C57BL/6 mouse controls, albeit with lower IgG3 and IgG2b, possibly as a result of lower levels of TGF-β, which, in C57BL/6 mice, is secreted by T cells (Fig. 2C) (*37*). By contrast, *Tcr*β*^−/−^Tcr*δ*^−/−^* mice injected with nil (alum only) did not make these flagellin-specific antibodies or ASCs. Flagellin-injected *Tcr*β*^−/−^Tcr*δ*^−/−^* mice, however, made some flagellin-specific IgG1 despite the lack of T cells—CSR to IgG1 is directed by IL-4, which is made by not only T cells but also alum-induced GR1^+^ cells, such as eosinophils (*38*, *39*), which are present in *Tcr*β*^−/−^Tcr*δ*^−/−^* mice. Further supporting the intrinsic B cell–activating role of TLR5-BCR linked coengagement, in vitro stimulation of purified naïve IgM^+^IgD^+^ B cells by flagellin induced class-switched flagellin-specific IgG3 and IgG2b, as secreted by IgG3 and IgG2b ASCs (fig. S6, B and C). Thus, induction of a specific and class-switched antibody response in the absence of T cells extends to B cell TLR5-BCR linked coengagement, as mediated by *S.* Typhimurium flagellin.

### TLR-BCR linked coengagement activates the canonical and noncanonical NF-κB pathways

We have previously shown that TLR4 and TLR9 signaling synergizes with BCR signaling to activate both the canonical and noncanonical nuclear factor κΒ (NF-κΒ) pathways to induce AID expression as CD40:CD154 engagement does (*24*). To assess that TLR5-BCR linked coengagement can also activate the B cell canonical and noncanonical NF-κB pathways, we analyzed the kinetics of inhibitor of NF-κBα (IκBα) and p65 phosphorylation (canonical pathway) together with p100 to p52 processing (noncanonical pathway) in B cells stimulated with *S.* Typhimurium flagellin, NP-LPS, or mCD154 plus IL-4 as a T-dependent control. Similar to NP-LPS, flagellin induced IκBα and p65 phosphorylation, which peaked at 10 to 15 min after stimulation, as was also the case in B cells stimulated by mCD154 plus IL-4 (Fig. 2D). Consistent with what we previously found (*24*, *25*), NP-LPS and mCD154 plus IL-4 induced peak levels of B cell p100 conversion to p52 within 3 and 24 hours, respectively. By contrast, flagellin induced B cell NF-κB p100 and p100 conversion to p52 within 5 to 60 min. (Fig. 2D), followed by exhaustion of such conversion and reversion to p100 expression only by 3 hours (fig. S6D). Thus, linked coengagement of B cell TLR4-BCR by NP-LPS or TLR5-BCR by flagellin activates both the canonical and noncanonical NF-κB pathways.

### B cell TLR4-BCR and TLR5-BCR linked coengagements induce antibody responses in NSG/B mice

**Fig. 3.**
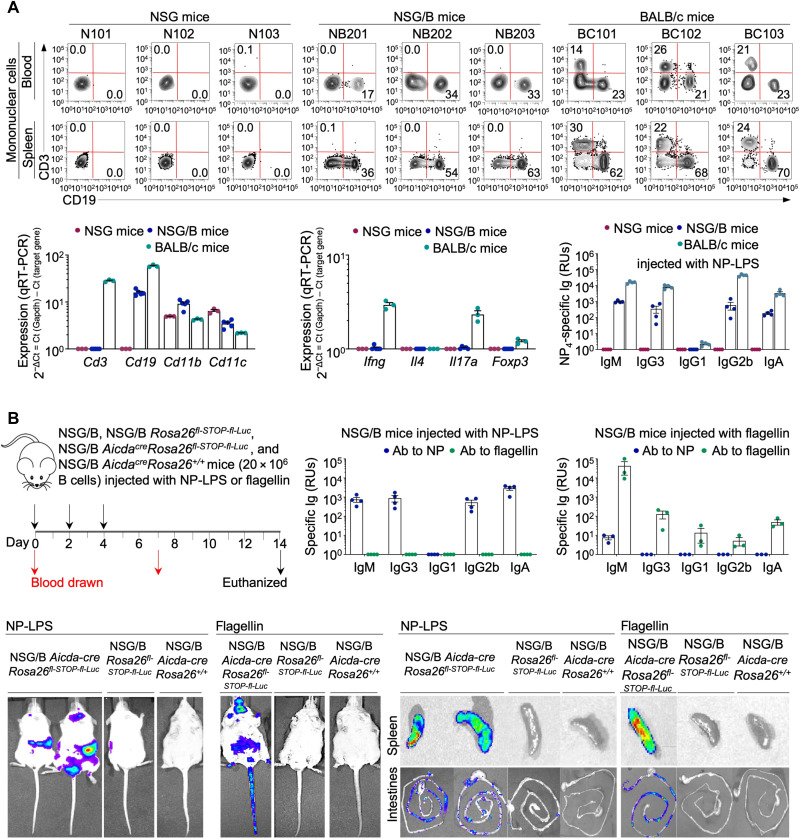
Induction of T-independent B cell–intrinsic class-switched antibody responses to NP and flagellin in NSG/B mice. (**A**) NSG, NSG/B, and BALB/c mice (*n* = 3 per group) were injected intraperitoneally with NP-LPS; peripheral blood and spleen B and T cells were identified (percentages of total mononuclear cells) by anti-CD19 and anti-CD3 mAbs. Spleen mononuclear cells were analyzed for *CD3*, *CD19*, *CD11b*, *CD11c*, and cytokine transcripts by quantitative reverse transcription polymerase chain reaction (qRT-PCR) (normalized to *Gapdh*, 2^−ΔCt^ method). NSG, NSG/B, and BALB/c mice (*n* = 4) were injected intraperitoneally with NP-LPS at days 0, 2, and 4. NP_4_-specific IgM, IgG3, IgG1, IgG2b, and IgA were measured by ELISA (titers are RUs) at day 14. Histograms depict mean values ± SEM. Each dot represents an individual mouse. (**B**) NSG/B, NSG/B *Rosa26^fl-STOP-fl-Luc^*, NSG/B *Aicda-creRosa26^fl-STOP-fl-Luc^*, and NSG/B *Aicda-cre Rosa26^+/+^* mice were constructed by grafting NSG mice with B cells (2.0 × 10^7^) from C57BL/6, *Rosa26^fl-STOP-fl-Luc^*, *Aicda-cre Rosa26^fl-STOP-fl-Luc^*, or *Aicda-cre Rosa26^+/+^* mice. NSG/B mice were injected intraperitoneally with NP-LPS (*n* = 5) or *S.* Typhimurium flagellin (*n* = 3) at days 0, 2, and 4. Serum NP_4_-specific and flagellin-specific IgM, IgG3, IgG1, IgG2b, and IgA were measured by ELISA (titers are RUs) at day 14. Histograms depict mean values ± SEM. Dots are individual mice. NSG/B *Rosa26^fl-STOP-fl-Luc^*, NSG/B *Aicda-cre Rosa26^fl-STOP-fl-Luc^*, and NSG/B *Aicda-cre Rosa26^+/+^* mice (*n* = 2 per genotype) were injected intraperitoneally with NP-LPS or flagellin under the same schedule. AID was identified in the spleen, intestines, and splanchnic district by luciferase bioluminescence imaging. Ab, antibody.

To further prove the role of B cell–intrinsic TLR-BCR linked coengagement in the T-independent antibody response, we constructed NSG/B mice by engrafting immunodeficient NOD.Cg-*Prkdc^scid^ Il2rg^tm1Wjl^*/SzJ (NSG) mice with highly purified B cells from C57BL/6 mice (NSG/B mice) (Fig. 3A). NSG mice lack B, T, and natural killer (NK) cells. They have dendritic (CD11c^+^) cells (DCs) and monocytes (CD11b^+^ cells), but these cells are functionally defective (*40*) and do not express IL-4, IL-17, and FoxP3 (Fig. 3A and fig. S7A). Upon injection with NP-LPS, NSG/B but not NSG mice made total (fig. S7B) and specific high-affinity IgG3, IgG2b, and IgA (in addition to IgM) antibodies to NP, as BALB/c mice did (Fig. 3A). In NSG/B mice, NP-specific high-affinity IgA antibodies were elicited after three NP-LPS injections, in contrast with the virtually nondetectable levels of NP-specific IgA in *Tcr*β*^−/−^Tcr*δ*^−/−^* mice injected only once with NP-LPS, as in other experiments (Figs. 1A and 6B). To prove the specificity of the TLR4-BCR and TLR5-BCR linked coengagement–mediated antibody response, we injected NSG/B mice with NP-LPS or *S.* Typhimurium flagellin and tested them for both specific anti-NP antibodies and anti-flagellin antibodies. NSG/B mice injected with NP-LPS made specific high-affinity IgG3, IgG2b, and IgA to NP but not flagellin, as secreted by NP-specific ASCs (Fig. 3B and fig. S7C). The effect, if any, that B cell activating factor (BAFF) might have had on B cells in NSG/B mice was not determined. Conversely, NSG/B mice injected with flagellin made specific IgG3, IgG2b, IgA, and IgG1 to flagellin but not NP, as secreted by flagellin-specific ASCs (Fig. 3B and fig. S7C) and as flagellin-immunized *Tcr*β*^−/−^Tcr*δ*^−/−^* mice did (Fig. 2C). TLR4-BCR– and TLR5-BCR–mediated, class-switched specific antibody responses in NSG/B mice reflected B cell AID expression in the spleen, small intestine, and splanchnic district at large, as shown by NSG/B *Aicda-cre Rosa26^fl-STOP-fl-Luc^* mice but not control NSG/B *Rosa26^fl-STOP-fl-Luc^* and NSG/B *Aicda-cre Rosa26^+/+^* mice injected with NP-LPS or flagellin (Fig. 3B). Thus, B cell TLR4-BCR and TLR5-BCR linked coengagements induce class-switched and specific antibody responses in the absence of T cells and virtually all other immune cells.

### The T-independent class-switched B cell TLR4-BCR linked coengagement–induced response is clonal and hypermutated

**Fig. 4.**
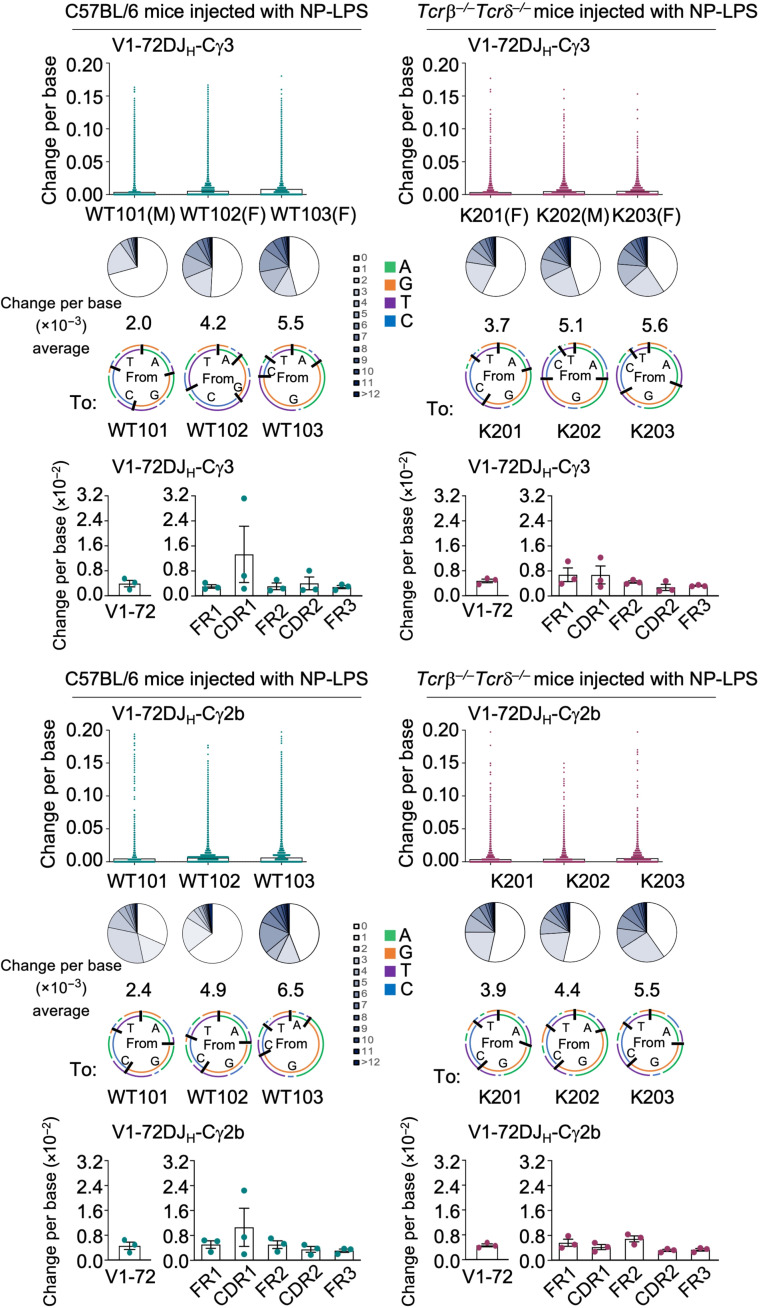
SHM of the class-switched anti-NP antibody response mediated by TLR4-BCR linked coengagement in *Tcr*β*^−/−^Tcr*δ*^−/−^* mice. C57BL/6 and *Tcr*β*^−/−^Tcr*δ*^−/−^* mice were injected intraperitoneally with NP-LPS on days 0 and 21 (*n* = 3 mice per group, one male and two females). Mice were euthanized 14 days after the second injection (day 35). V1-72DJ_H_-Cγ3 and V1-72DJ_H_-Cγ2b transcripts were analyzed by Illumina MiSeq amplicon sequencing. Sequence analysis allowed for identification of point mutations in the V1-72 region of V1-72DJ_H_-Cγ3 and V172DJ_H_-Cγ2b transcripts in C57BL/6 and *Tcr*β*^−/−^Tcr*δ*^−/−^* mice. Dots depict mutation frequencies in each sequence. Pie chart slices depict the proportions of transcripts carrying given numbers of point mutations; slice gray gradients depict increasing numbers of point mutations (from 0 to more than 12) per transcript. Numbers below pie charts depict average overall point-mutation frequency (change per base) of all transcripts. Donut charts depict the nature of point mutations. Histograms depict the overall mutation frequency (change per base) and frequency of point mutations in V1-72 framework regions (FRs) and complementarity determining regions (CDRs) within V1-72DJ_H_-Cγ3 and V1-72DJ_H_-Cγ2b transcripts. Each dot represents the mean of approximately 100,000 sequences per mouse ± SEM.

Similar to CSR, SHM requires B cell AID expression and has been long thought to be dependent on T cell help (*1*, *10*, *30*). After immunization of *Tg(Aicda-cre)Rosa26^fl-STOP-fl-Luc^* mice with NP-LPS, we readily detected AID expression in the spleen and lymph nodes, which harbor secondary lymphoid formations where B cells undergo SHM/CSR (Fig. 1D). In our previous studies involving NP-LPS–injected NSG/B mice, the anti-NP V186.2DJ_H_-Cγ2b response (V186.2, V1-72 according to the International ImMunoGeneTics (IMGT) nomenclature, encodes the V_H_ segment that binds NP) was extensively mutated (*27*), strongly suggesting that TLR-BCR linked coengagement induced B cell SHM in the absence of T cells. Accordingly, V1-72DJ_H_-Cγ3 and V1-72DJ_H_-Cγ2b transcripts from NP-LPS–injected *Tcr*β*^−/−^Tcr*δ*^−/−^* mice bore a high frequency of point mutations (4.8 ± 0.6 × 10^−3^ and 4.6 ± 0.5 × 10^−3^ change per base, means ± SEM, respectively) at levels at least comparable to those in V1-72DJ_H_-Cγ3 and V1-72DJ_H_-Cγ2b transcripts of control NP-LPS–injected C57BL/6 mice (3.9 ± 0.1 × 10^−3^ and 4.6 ± 0.1 × 10^−3^ change per base, respectively; Fig. 4) and slightly higher than those in V1-72DJ_H_-Cγ1 transcripts of NP-CGG–injected C57BL/6 mice (2.6 ± 0.3 × 10^−3^ change per base; fig. S8A). As in C57BL/6 mice, more than 75% of point mutations in *Tcr*β*^−/−^Tcr*δ*^−/−^* mice were replacement (R) mutations (overall R:S ratio of over 3), including codons encoding characteristic V1-72 NP-binding residues, such as the CDR1 5′-TGG-3′ to 5′-TTG-3′ transversion yielding Trp33 to Leu replacement (W33L), CDR1 5′-ATG-3′ to 5′-ATA-3′ transition yielding Met34 to Ile replacement (M34I), CDR2 5′-AAG-3′ to 5′-AGG-3′ transition yielding Lys59 to Arg replacement (K59R), and 5′-AGC-3′ to 5′-AAC-3′ transition yielding Ser66 to Asn replacement (S66N) (figs. S8B, S9, S10, and S11A).

To address the NP-specific B cell clonality induced by NP-LPS, B cells expressing V1-72DJ_H_-Cγ3 and V1-72DJ_H_-Cγ2b transcripts were segregated on the basis of the V1-72 gene segment, the same IgH CDR3 (IgH CDR3 sequences are highly diverse as they stem from somatic *V_H_DJ_H_* gene recombination events) and the same J_H_ gene sequence in three *Tcr*β*^−/−^Tcr*δ*^−/−^* and three C57BL/6 mice injected with NP-LPS. As a T-dependent control, V1-72DJ_H_-Cγ1 transcripts from three C57BL/6 mice injected with NP-CGG were segregated using the same criteria. In NP-LPS–injected *Tcr*β*^−/−^Tcr*δ*^−/−^* mice, V1-72DJ_H_-Cγ3 and V1-72DJ_H_-Cγ2b CDR3s consisted of 11 to 12 (11.00 ± 1.23, means ± SD) and 7 to 14 (11.44 ± 2.46) amino acids, respectively, lengths comparable to the 11 to 14 (12.44 ± 1.13) and 11 to 16 (13.00 ± 2.00) amino acids of V1-72DJ_H_-Cγ3 and V1-72DJ_H_-Cγ2b CDR3s in NP-LPS–injected C57BL/6 mice and the 10 to 16 (12.67 ± 1.66) amino acids of V1-72DJ_H_-Cγ1 CDR3s in NP-CGG–injected mice (table S1).

**Fig. 5.**
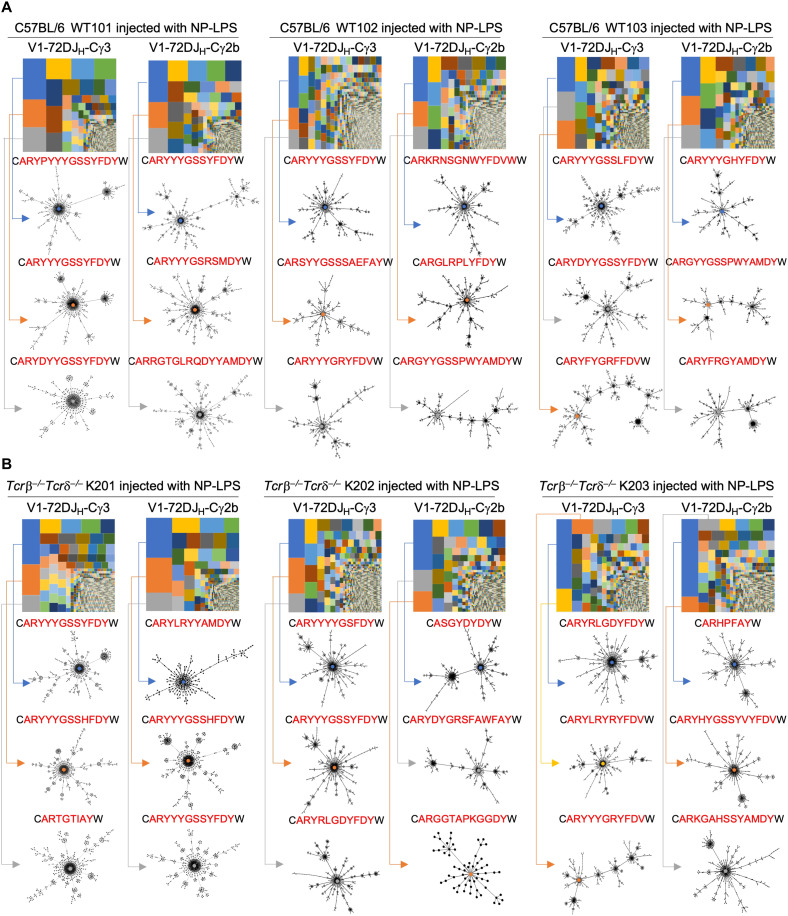
B cell clonality and intraclonal diversification of the class-switched anti-NP antibody response mediated by TLR4-BCR linked coengagement in *Tcr*β*^−/−^Tcr*δ*^−/−^* mice. C57BL/6 and *Tcr*β*^−/−^Tcr*δ*^−/−^* mice were injected intraperitoneally with NP-LPS on days 0 and 21 (*n* = 3 mice per group). Sequence analysis of V1-72DJ_H_-Cγ3 and V1-72DJ_H_-Cγ2b transcripts in (**A**) C57BL/6 mice and (**B**) *Tcr*β*^−/−^Tcr*δ*^−/−^* mice 14 days after the second injection (day 35) allowed for identification of B cell clones by segregation of V1-72DJ_H_-Cγ3 and V1-72DJ_H_-Cγ2b transcripts consisting of the V1-72 gene segment, the same and unique IgH CDR3 (depicted as translated amino acid sequence) together with the same J_H_ gene sequence. Each discrete clone is depicted as an individual rectangle or square of a unique color, whose area reflected the B cell clone size. Intraclonal diversification is depicted for each of the three “dominant” clones as a genealogical tree (phylogenic map) constructed based on shared and unique V1-72DJ_H_-Cγ3 and V1-72DJ_H_-Cγ2b point mutations. Trees reveal SHM-mediated complex intraconal diversification from unmutated progenitors (color-coded according to respective dominant clones).

In the same three NP-LPS–injected *Tcr*β*^−/−^Tcr*δ*^−/−^* mice and three NP-LPS–injected C57BL/6 mice, a total of 1597 to 5839 V1-72DJ_H_-Cγ3 and 1662 to 4938 V1-72DJ_H_-Cγ2b recombined and discrete V1-72DJ_H_ clones were identified. In K201 and K202 *Tcr*β*^−/−^Tcr*δ*^−/−^* mice and in each of the three C57BL/6 mice (WT101, WT102, and WT103), the overall B cell clones could be segregated into 3 dominant clones (both V1-72DJ_H_-Cγ3 and V1-72DJ_H_-Cγ2b), 10 to 16 (V1-72DJ_H_-Cγ3) and 9 to 16 (V1-72DJ_H_-Cγ2b) intermediate clones, 32 to 60 (V1-72DJ_H_-Cγ3) and 12 to 45 (V1-72DJ_H_-Cγ2b) small clones, and lastly, 2212 to 5764 (V1-72DJ_H_-Cγ3) and 1817 to 4878 microclones (V1-72DJ_H_-Cγ2b). The B cell clones in the third *Tcr*β*^−/−^Tcr*δ*^−/−^* mouse (K203) were segregated into two dominant clones (both V1-72DJ_H_-Cγ3 and V1-72DJ_H_-Cγ2b), 10 (V1-72DJ_H_-Cγ3) and 11 (V1-72DJ_H_-Cγ2b) intermediate clones, 17 (V1-72DJ_H_-Cγ3) and 13 (V1-72DJ_H_-Cγ2b) small clones, and 1568 (V1-72DJ_H_-Cγ3) and 1636 (V1-72DJ_H_-Cγ2b) microclones (TreeMap plots; Fig. 5, A and B, and tables S2 and S3). In the three NP-CGG–injected C57BL/6 mice, a total of 1760 to 5894 V1-72DJ_H_-Cγ1 recombined and discrete clones were identified. The total B cell clones could be segregated into 2 to 3 dominant clones, 8 to 17 intermediate clones, 86 to 110 small clones, and 1656 to 5764 microclones (table S4). The size and the number of dominant clones in *Tcr*β*^−/−^Tcr*δ*^−/−^* mice were comparable to those in NP-LPS–injected and NP-CGG–injected C57BL/6 mice.

The shared and unique point mutations in V1-72DJ_H_-Cγ3 and V1-72DJ_H_-Cγ2b transcripts within the three dominant clones in each of the *Tcr*β*^−/−^Tcr*δ*^−/−^* and C57BL/6 mice injected with NP-LPS, as well as the three C57BL/6 mice injected with NP-CGG, allowed for construction of genealogical trees. In these, multiple branches emanating from a common progenitor revealed complex SHM-mediated intraconal diversifications, comparable in *Tcr*β*^−/−^Tcr*δ*^−/−^* and C57BL/6 mice injected with NP-LPS or NP-CGG (C57BL/6 mice only) (Fig. 5, A and B, and figs. S11B and S12). Thus, B cell TLR-BCR coengagement by NP-LPS induces, in the absence of T cells, a class-switched and somatically hypermutated antibody response to NP, entailing expansion of select B cell clones with intraconal diversifications, at degrees comparable to those of T cell–competent mice responding to NP-LPS or NP-CGG.

### B cell TLR4-BCR linked coengagement induces specific memory B cells in *Tcr*β*^−/−^Tcr*δ*^−/−^* mice

**Fig. 6.**
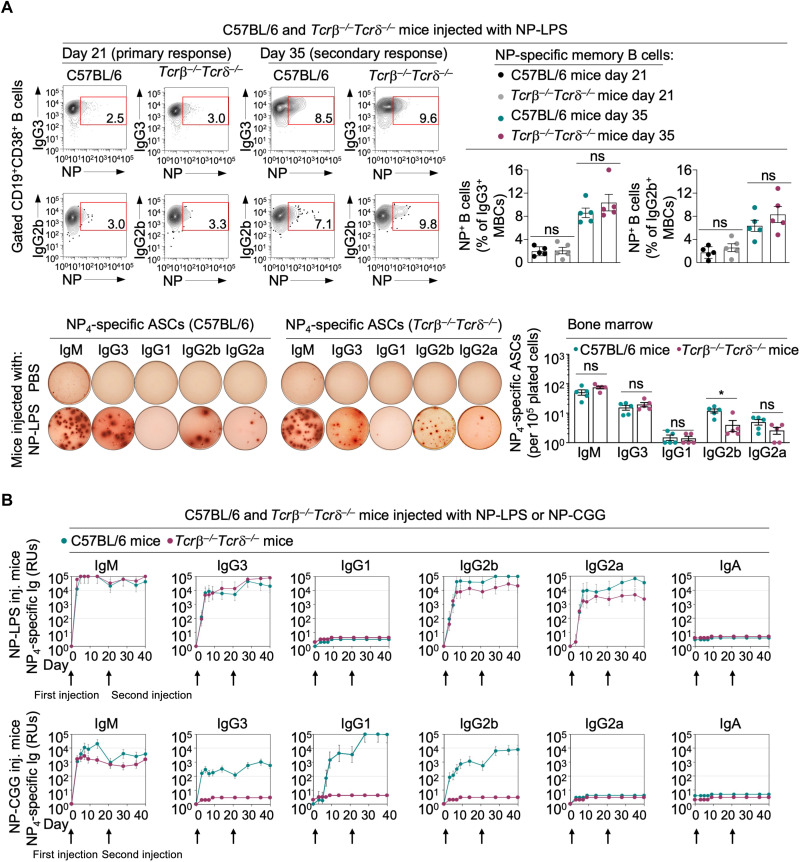
B cell TLR4-BCR linked coengagement induces generation of memory B cells and anamnestic response in *Tcr*β*^−/−^Tcr*δ*^−/−^* mice. (**A**) C57BL/6 and *Tcr*β*^−/−^Tcr*δ*^−/−^* mice were injected intraperitoneally with NP-LPS on days 0 and 21 (*n* = 5 mice per group) and euthanized on days 21 (primary response) or 35 (memory response) for analysis of the spleen and bone marrow cells. Class-switched NP-specific memory B cells (pregated CD19^+^CD38^+^IgG3^+^ and CD19^+^CD38^+^IgG2b^+^ cells) were identified in the spleens by binding of NP-PE, anti-CD19 and anti-CD38 mAbs, and anti-IgG3 and anti-IgG2b mAbs, as analyzed by flow cytometry (numbers are percentages of total mononuclear cells); negative control PE^+^ B cells were less than 0.5% (not shown). Histograms depict mean values of NP-specific CD19^+^CD38^+^IgG3^+^ and NP-specific CD19^+^CD38^+^IgG2b^+^ memory B cells ± SEM (% of IgG3^+^ or IgG2b^+^ memory B cells) in C57BL/6 and *Tcr*β*^−/−^Tcr*δ*^−/−^* mice on days 21 and 35. Each dot represents an individual mouse. NP-specific IgM, IgG3, IgG1, IgG2b, and IgG2a ASCs as detected by ELISPOT in the bone marrow of C57BL/6 and *Tcr*β*^−/−^Tcr*δ*^−/−^* mice, each dot representing an individual mouse; histograms depict mean numbers of ASCs per 10^5^ plated cells ± SEM. (**B**) Elicitation of a class-switched anamnestic antibody response in *Tcr*β*^−/−^Tcr*δ*^−/−^* mice. C57BL/6 and *Tcr*β*^−/−^Tcr*δ*^−/−^* mice were injected intraperitoneally with NP-LPS or NP-CGG on days 0 and 21 (*n* = 8 mice per group). Sera were collected on days 0, 3, 5, 7, 14, 21, 28, 35, and 40 and analyzed for NP_4_-specific IgM, IgG3, IgG1, IgG2b, IgG2a, and IgA by ELISA (titers expressed as RUs). Graphs depict NP_4_-specific antibody levels through day 40; data points are mean values ± SEM of eight mice at different times. *P < 0.05 (unpaired *t* test). MBCs, memory B cells.

Having shown that B cell–intrinsic TLR4-BCR coengagement by NP-LPS can induce a specific, class-switched and hypermutated antibody response in *Tcr*β*^−/−^Tcr*δ*^−/−^* mice, we wanted to determine whether such a response entailed the generation of memory B cells. To this end, we injected *Tcr*β*^−/−^Tcr*δ*^−/−^* and C57BL/6 mice with NP-LPS at day 0 (primary injection) and day 21 (recall). The primary response, as analyzed at day 21, yielded 3.0 and 3.3% NP-specific IgG3^+^ and IgG2b^+^ memory (CD19^+^CD38^+^) B cells out of total IgG3^+^ and IgG2b^+^ memory (CD19^+^CD38^+^) B cells in NP-LPS–injected *Tcr*β*^−/−^Tcr*δ*^−/−^* mice and yielded 2.5 and 3.0% NP-specific IgG3^+^ and IgG2b^+^ memory B cells in C57BL/6 mice. At day 35 (14 days after the second NP-LPS injection), the recall response yielded 9.6 and 9.8% NP-specific IgG3^+^ and IgG2b^+^ memory B cells out of total IgG3^+^ and IgG2b^+^ memory B cells in *Tcr*β*^−/−^Tcr*δ*^−/−^* mice versus 8.5 and 7.1% NP-specific IgG3^+^ and IgG2b^+^ memory B cells in C57BL/6 mice (Fig. 6A). In *Tcr*β*^−/−^Tcr*δ*^−/−^* mice, NP-specific memory B cells emerged together with increased NP-specific IgG3, IgG2b, and IgG2a ASCs in the bone marrow at levels comparable to those of C57BL/6 mice submitted to the same immunization schedule. To further analyze the T-independent memory B cell response, we boosted an additional cohort of *Tcr*β*^−/−^Tcr*δ*^−/−^* mice 21 days after primary injection with NP-LPS (100 μg in PBS per mouse for primary and boost injection) and measured NP-specific antibodies as late as 20 days after the boost injection (Fig. 6B). Boost-injected *Tcr*β*^−/−^Tcr*δ*^−/−^* mice increased NP-specific high-affinity antibodies to levels approximately 2-fold (IgG2b) to 10-fold (IgG3) greater than those of the primary response comparable to C57BL/6 mice with respect to IgG3 and borderline lower with respect to IgG2b and IgG2a. In C57BL/6 mice, IgG2b and IgG2a were increased possibly as a result of T cell–secreted TGF-β and IFN-γ, respectively (*2*, *37*). As expected, *Tcr*β*^−/−^Tcr*δ*^−/−^* mice did not mount a NP-specific IgG1 response, and neither *Tcr*β*^−/−^Tcr*δ*^−/−^* nor C57BL/6 mice made NP-specific IgA. Last, the T-independent, class-switched, anamnestic antibody response induced by NP-LPS–mediated TLR4-BCR linked coengagement was elicited as late as 90 days from primary injection and lasted unabated for at least 50 days in both young and old BALB/c mice (fig. S13). Thus, T-independent intrinsic B cell TLR-BCR linked coengagement induces generation of memory B cells supporting a specific, class-switched and long-lasting anamnestic antibody response.

### Intrinsic B cell TLR5-BCR linked coengagement induces a clonal, hypermutated, class-switched, and anamnestic antibody response in *Tcr*β*^−/−^Tcr*δ*^−/−^* mice

**Fig. 7.**
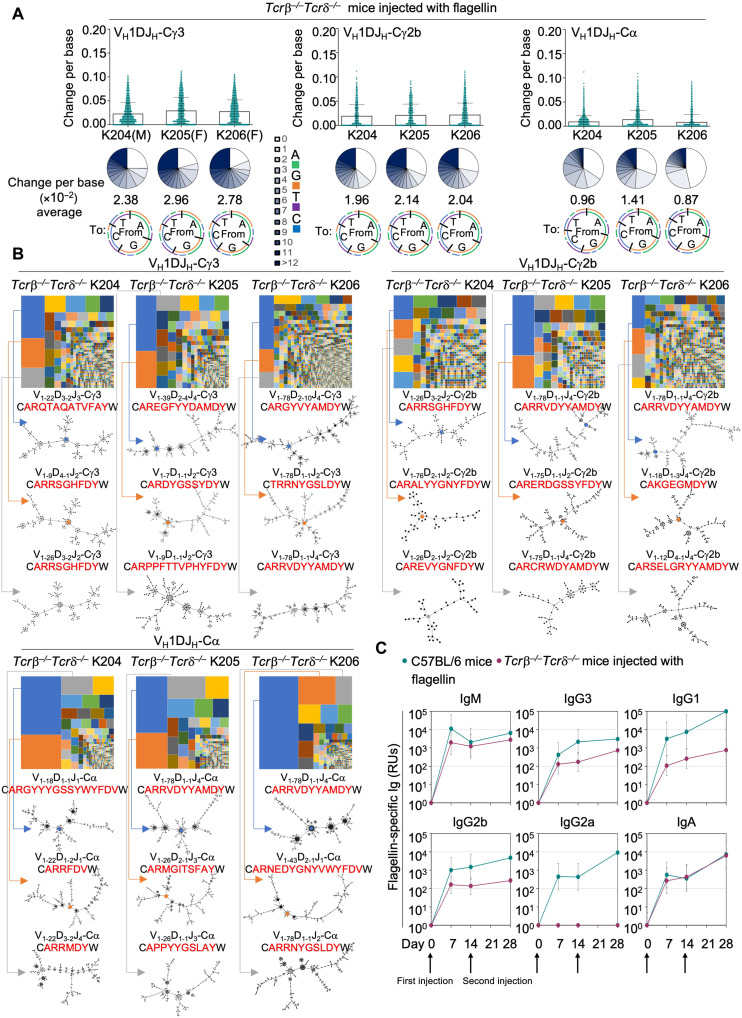
TLR5-BCR linked coengagement by *S.* Typhimurium flagellin induces SHM/CSR, clonal B cell expansion, and intraclonal diversification in *Tcr*β^−/−^*Tcr*δ^−/−^ mice. *Tcr*β*^−/−^Tcr*δ*^−/−^* mice were injected intraperitoneally with flagellin on days 0 and 14 (*n* = 3 mice, one male and two females) and euthanized on day 28. V_H_1DJ_H_-Cγ3, V_H_1DJ_H_-Cγ2b, and V_H_1DJ_H_-Cα transcripts from the *Tcr*β*^−/−^Tcr*δ*^−/−^* mice were analyzed by Illumina MiSeq amplicon sequencing, as described in Fig. 4. (**A**) Sequence analysis allowed for identification of point mutations in the V_H_1 region of V_H_1DJ_H_-Cγ3, V_H_1DJ_H_-Cγ2b, and V_H_1DJ_H_-Cα transcripts. Dots depict mutation frequencies in each sequence. Pie chart slices depict proportions of transcripts carrying given numbers of point mutations; slice gray gradients depict increasing numbers of point mutations (0 to more than 12) per transcript. Numbers below pie charts depict average overall point-mutation frequency (change per base) of all transcripts. Donut charts depict the nature of point mutations. (**B**) Each B cell clone was identified on the basis of segregation of V_H_1DJ_H_-Cγ3, V_H_1DJ_H_-Cγ2b, and V_H_1DJ_H_-Cα transcripts consisting of the V_H_1 gene segment, the same and unique IgH CDR3 (depicted as translated amino acid sequence) together with the same J_H_ sequence by MiSeq amplicon sequencing. Size and complex intraclonal SHM diversification (genealogical tree) of each discrete clone are depicted, as described in Fig. 5. (**C**) C57BL/6 mice and *Tcr*β*^−/−^Tcr*δ*^−/−^* mice were injected intraperitoneally with flagellin on days 0 and 14 (*n* = 3 mice per group). Sera were collected on days 0, 7, 14, and 28 and analyzed for flagellin-specific IgM, IgG3, IgG1, IgG2b, IgG2a, and IgA (titers expressed as RUs). Graphs depict flagellin-specific antibody levels through day 28; data points are mean values ± SEM of three mice at different times.

Members of the mouse V_H_1 gene family are known to be the major contributors to the specific class-switched IgG and IgA antibody response to *S.* Typhimurium (*41*). Therefore, to analyze the antibody response induced by TLR5-BCR linked coengagement, we sequenced V_H_1DJ_H_-Cμ and class-switched V_H_1DJ_H_-Cγ3, V_H_1DJ_H_-Cγ2b, and V_H_1DJ_H_-Cα transcripts in three flagellin-injected *Tcr*β*^−/−^Tcr*δ*^−/−^* mice. In these mice, B cell V_H_1DJ_H_-Cμ, V_H_1DJ_H_-Cγ3, V_H_1DJ_H_-Cγ2b, and V_H_1DJ_H_-Cα transcripts bore great loads of point mutations: 1.5 ± 0.2 × 10^−2^, 2.7 ± 0.3 × 10^−2^, 2.0 ± 0.1 × 10^−2^, and 1.1 ± 0.3 × 10^−2^ change per base (means ± SEM), respectively (Fig. 7A and fig. S14). The V_H_1DJ_H_-Cγ3, V_H_1DJ_H_-Cγ2b, and V_H_1DJ_H_-Cα transcripts point-mutation frequencies were virtually an order of magnitude greater than those induced by NP-LPS in recombined V1-72 gene of *Tcr*β*^−/−^Tcr*δ*^−/−^* (4.8 ± 0.6 × 10^−3^ and 4.6 ± 0.5 × 10^−3^ change per base; *P* < 0.001 and *P* < 0.001, respectively) and C57BL/6 mice (3.9 ± 0.1 × 10^−3^ and 4.6 ± 0.1 × 10^−3^ change per base; *P* < 0.001 and *P* < 0.001, respectively; Fig. 4) or those induced by NP-CGG in recombined V1-72 gene of C57BL/6 mice (flagellin in *Tcr*β*^−/−^Tcr*δ*^−/−^* mice V_H_1DJ_H_-Cγ3 and V_H_1-DJ_H_-Cγ2b mutations versus NP-CGG in C57BL/6 mice V1-72DJ_H_-Cγ1, 2.6 ± 0.03 × 10^−3^ change per base; *P* < 0.001 and *P* < 0.001; fig. S8A). Similarly, the V_H_1DJ_H_-Cα mutation frequency in three flagellin-injected *Tcr*β*^−/−^Tcr*δ*^−/−^* mice was twofold greater than those in V1-72DJ_H_-Cγ3 and V_H_1-72DJ_H_-Cγ2b transcripts of NP-LPS–injected *Tcr*β*^−/−^Tcr*δ*^−/−^* and C57BL/6 mice (*P* = 0.001 and *P* = 0.003, respectively) as well as that in V1-72DJ_H_-Cγ1 of NP-CGG–injected C57BL/6 mice (*P* = 0.008).

Segregation of V_H_1DJ_H_-C_X_ transcripts based on the same criteria (identical V_H_-CDR3-J_H_ sequence) used in NP-LPS–injected *Tcr*β*^−/−^Tcr*δ*^−/−^* mice together with inspection of rearranged V_H_1 somatic point mutations allowed us to define B cell clonality and analyze intraclonal diversification in three flagellin-injected *Tcr*β*^−/−^Tcr*δ*^−/−^* mice. In these mice, a total of 717, 883, and 3308 V_H_1DJ_H_-Cγ3; 350, 560, and 1146 V_H_1DJ_H_-Cγ2b; and 371, 441, and 502 V_H_1DJ_H_-Cα clones were identified (Fig. 7B), with the V_H_1DJ_H_-Cγ3, V_H_1DJ_H_-Cγ2b, and V_H_1DJ_H_-Cα CDR3s consisting of 9 to 14 (10.78 ± 1.64, means ± SD), 8 to 13 (10.78 ± 1.56), and 6 to 15 (10.44 ± 3.21) amino acids, respectively (table S5). The total B cell clones could be segregated into 2 to 4, 1 to 3, and 1 to 2 (V_H_1DJ_H_-Cγ3, V_H_1DJ_H_-Cγ2b and V_H_1DJ_H_-Cα) dominant clones; 13 to 72, 8 to 9, and 15 to 24 intermediate clones; 79 to 366, 27 to 63, and 20 to 41 small clones; and 576 to 2866, 303 to 1072, and 332 to 442 microclones (table S6). The large sizes of the dominant V_H_1DJ_H_-Cα clones possibly reflected the effectiveness of flagellin to induce IgA responses (*36*).

**Fig. 8.**
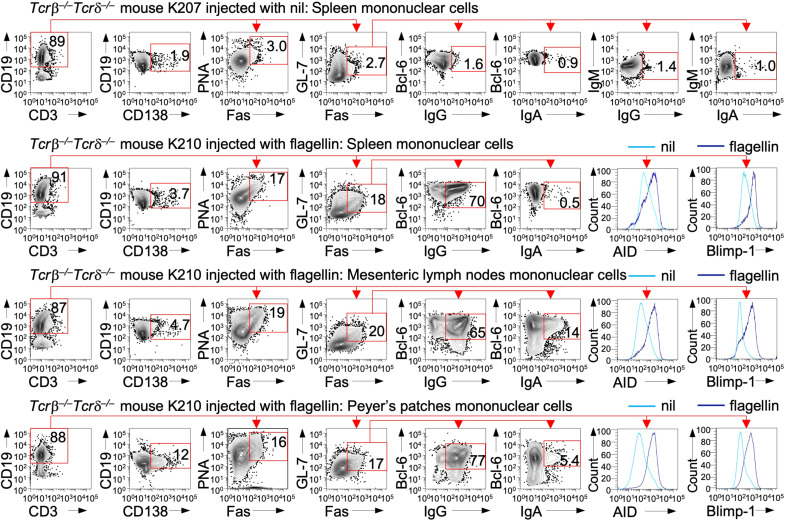
*S.* Typhimurium flagellin TLR5-BCR linked coengagement–mediated antibody response entails GC-like B cell and plasma cell differentiation in *Tcr*β^−/−^*Tcr*δ^−/−^ mice. *Tcr*β*^−/−^Tcr*δ*^−/−^* mice were injected intraperitoneally on day 0 with nil (alum, K207) or flagellin in alum (K210) and again at day 7 with PBS or flagellin in PBS, respectively. Mice were euthanized 7 days after the second injection (day 14). The spleen, mesenteric lymph nodes (MLNs), and Peyer’s patches mononuclear cells were analyzed by fluorescence-activated cell sorting for PNA^+^GL7^+^CD95^+^ GC-like B cells, as identified by staining with PNA and anti-CD19, anti-GL7, anti-CD95 (Fas), anti-Bcl6 (intracellular staining), anti-IgM, anti-IgG, and anti-IgA mAbs. Plasmablasts were identified by surface staining with anti-CD19 and anti-CD138 mAbs. AID and Blimp-1 expression were analyzed in B cells of the spleens, MLNs, and Peyer’s patches in nil-injected and flagellin-injected mice and were detected by intracellular staining with anti-AID and anti–Blimp-1 mAbs followed by flow cytometry analysis. Numbers are percentages of total mononuclear cells or total B cells. Data from additional nil-injected (*n* = 2 mice: K208 and K209) and flagellin-injected (*n* = 2 mice: K211 and K212) *Tcr*β*^−/−^Tcr*δ*^−/−^* mice are displayed in fig. S15.

To determine whether *S.* Typhimurium flagellin induced an anamnestic antibody response in the absence of T cells, we injected *Tcr*β*^−/−^Tcr*δ*^−/−^* and C57BL/6 mice with flagellin (50 μg in alum), followed by a boost (50 μg in PBS) 14 days later—*Salmonella*-specific anti-flagellin antibodies are induced shortly after flagellin vaccination and at early stages after *S.* Typhimurium infection (*32*, *41*). Boost-injected *Tcr*β*^−/−^Tcr*δ*^−/−^* and C57BL/6 mice responded with increased flagellin-specific IgG3, IgG1, IgG2b, and IgA antibodies at levels approximately 2-fold (IgG2b) to 15-fold (IgA) greater than those of the primary response (IgG2a was not made by *Tcr*β*^−/−^Tcr*δ*^−/−^* mice because of the lack of T cell–secreted IFN-γ) (Fig. 7C). The *Tcr*β*^−/−^Tcr*δ*^−/−^* mice IgG3 and IgG2b response was overall lower than in C57BL/6 mice, in which IgG3 and IgG2b production was likely boosted by TGF-β and IFN-γ (*2*, *30*), as secreted by T cells. The production of flagellin-specific antibodies in *Tcr*β*^−/−^Tcr*δ*^−/−^* mice stemmed from emergence of PNA^+^Fas^+^ and GL7^+^Fas^+^, Bcl6^+^IgG^+^ and Bcl6^+^IgA^+^ GC-like B cells, and CD19^+^CD138^+^ plasmablasts, with expression of AID and Blimp-1, throughout the spleen, mesenteric lymph nodes, and small intestine Peyer’s patches (Fig. 8 and fig. S15). The flagellin-induced specific IgG and IgA antibody response including *Aicda* expression in splanchnic lymphoid organs was reproduced in NSG/B mice (Fig. 3B). Thus, TLR5-BCR linked coengagement by flagellin can induce a clonal, class-switched, hypermutated and specific antibody response involving B cell differentiation to GC-like cells and plasmablasts without T cell help.

### Naïve B cells express TLR4 and TLR5, which are increased by TLR-BCR linked coengagement

**Fig. 9.**
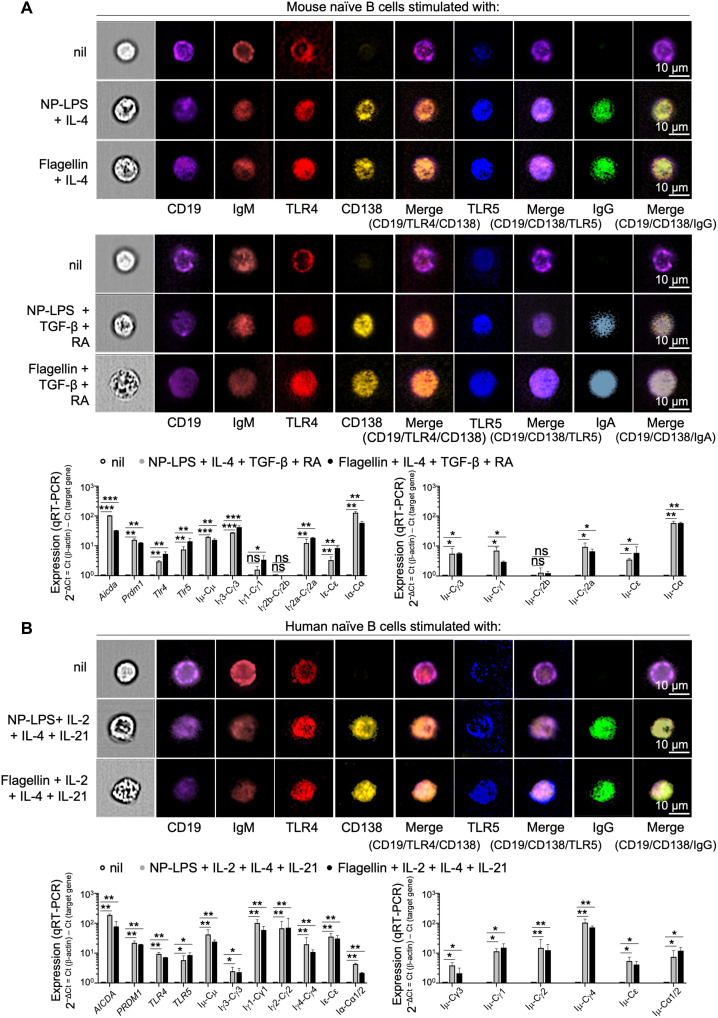
TLR-BCR linked coengagement induces CSR and plasma cell differentiation with increasing surface TLR4/TLR5 expression in mouse and human B cells. (**A**) Naïve IgM^+^IgD^+^ B cells from C57BL/6 mice were stimulated with nil (unstimulated), NP-LPS plus IL-4, NP-LPS plus TGF-β, and retinoic acid (RA); *S.* Typhimurium flagellin plus IL-4; or flagellin plus TGF-β and RA. At 96 hours, cultured cells were analyzed for surface CD19, TLR4, TLR5, and CD138; intracellular IgM, IgG, and IgA by single-cell flow imaging (scale bars, 10 μm); as well as for *Aicda, Prdm1*, *Tlr4, Tlr5,* germline I_H_-C_H_, and post-recombination Iμ-Cx (Cx being any switched isotype) transcripts, as measured by qRT-PCR and normalized to *Gapdh* expression (2^−ΔCt^ method). (**B**) Human naïve IgM^+^IgD^+^ B cells were stimulated with nil (unstimulated), NP-LPS plus huIL-2, huIL-4, and huIL-21 or *S.* Typhimurium flagellin plus huIL-2, huIL-4, and huIL-21. After 96 hours, cultured human B cells were analyzed for surface CD19, TLR4, TLR5, and CD138; intracellular IgM and IgG by single-cell flow imaging (scale bars, 10 μm); as well as for *AICDA, PRDM1*, *TLR4, TLR5,* germline I_H_-C_H_, and post-recombination Iμ-Cx (Cx being any switched isotype) transcripts, as measured by qRT-PCR and normalized to β*-ACTIN* expression (2^−ΔCt^ method). *P < 0.05, **P < 0.01, and ***P < 0.001 (unpaired *t* test).

We found TLR4 and TLR5 to be expressed, albeit at low levels, on the surface of mouse and human naïve IgM^+^IgD^+^ B cells. To address the dynamics of B cell TLR4 and TLR5 expression, we stimulated in vitro mouse (C57BL/6) naïve IgM^+^IgD^+^ B cells with NP-LPS (3 μg/ml) or *S.* Typhimurium flagellin (10 μg/ml) plus IL-4 (4 ng/ml) and NP-LPS or flagellin plus TGF-β (4 ng/ml) and retinoic acid (RA; 4 ng/ml). We also stimulated in vitro human naïve IgM^+^IgD^+^ B cells with NP-LPS or *S.* Typhimurium flagellin plus recombinant human IL-4 (huIL-4) (20 ng/ml), recombinant human IL-2 (huIL-2) (100 ng/ml), and recombinant human IL-21 (huIL-21) (50 ng/ml). These stimuli induced mouse and human B cells to undergo CSR to IgG and IgA and differentiation to CD19^+^CD138^+^ plasmablasts, which increased expression of both TLR4 and TLR5 with similar kinetics (Fig. 9, A and B; figs. S16, A and B, S17, A and B, and S18; and table S7). CSR, plasma cell differentiation, and TLR expression were underlined by expression of *Aicda*, *Prdm1*, *Tlr4*, and *Tlr5*; germline Iγ3-Cγ3, Iγ1-Cγ1, Iγ2b-Cγ2b, Iγ2a-Cγ2a, Iε-Cε, and Iα-Cα; and post-recombination Iμ-Cγ3, Iμ-Cγ1, Iμ-Cγ2b, Iμ-Cγ2a, Iμ-Cε, and Iμ-Cα transcripts in mouse B cells, and similarly, *AICDA*, *PRDM1*, *TLR4*, and *TLR5*; germline Iγ3-Cγ3, Iγ1-Cγ1, Iγ2-Cγ2, Iγ4-Cγ4, Iε-Cε, and Iα-Cα; and post-recombination Iμ-Cγ3, Iμ-Cγ1, Iμ-Cγ2, Iμ-Cγ4, Iμ-Cε, and Iμ-Cα transcripts in human B cells (Fig. 9, A and B). Thus, B cell TLR4-BCR or TLR5-BCR linked coengagement boosts TLR4 and TLR5 expression, concomitant with *Aicda/AICDA*, *Prdm1*/*PRDM1* expression, CSR, and plasma cell differentiation in mouse and human B cells.

### TLR4-BCR and TLR5-BCR linked coengagements by LPS and flagellin induce protective antibody responses to *E. coli* and *S.* Typhimurium in *Tcr*β*^−/−^Tcr*δ*^−/−^* mice

**Fig. 10.**
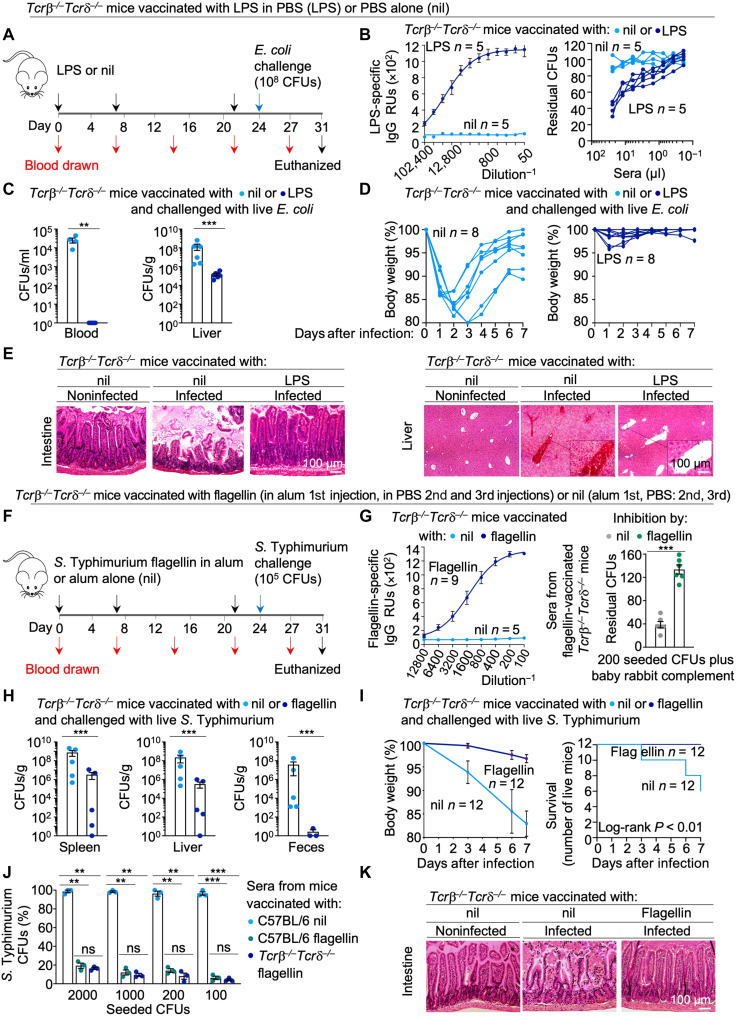
*E. coli* LPS and *S.* Typhimurium flagellin vaccination induces neutralizing antibodies against *E. coli* and *S.* Typhimurium in *Tcr*β*^−/−^Tcr*δ*^−/−^* mice. (**A**) *E. coli* challenge of nil (PBS)–injected and LPS-vaccinated *Tcr*β*^−/−^Tcr*δ*^−/−^* mice. (**B**) Left and right: Dose-dependent binding of serum LPS-specific IgGs and bactericidal activity (day 14, *n* = 5 mice per group). (**C**) Blood (day 27) and liver (day 31) bacterial load (*n* = 6 mice per group). (**D**) Body weight of infected mice (*n* = 8 per group). (**E**) Intestine and liver sections (H&E; scale bar, 100 μm) (representative of three mice per group). (**F**) *S.* Typhimurium challenge of nil (alum)- and flagellin (alum)-vaccinated *Tcr*β*^−/−^Tcr*δ*^−/−^* mice. (**G**) Left: Dose-dependent binding of serum flagellin-specific IgGs (day 14, *n* = 5 and *n* = 9 mice); right: Serum bactericidal activity, as inhibited by nil or free flagellin (*n* = 6 mice per group). (**H**) Bacterial load in the spleen, liver, and feces (day 31, *n* = 5 mice per group). (**I**) Left and right: Body weight and survival (Kaplan-Meier curves, *P* = 0.006, log-rank Mantel-Cox test) of infected mice vaccinated with nil or flagellin (*n* = 12 mice per group). (**J**) Wide-range bactericidal activity of sera from flagellin-vaccinated C57BL/6 and *Tcr*β*^−/−^Tcr*δ*^−/−^* mice (*n* = 3 per group). (**K**) Intestine sections (day 31, H&E; scale bar, 100 μm) (representative of three mice per group). Histograms and graphs depict mean values ± SEM. **P < 0.01 and ***P < 0.001 (unpaired *t* test).

The induction of a protective response to most infectious agents relies upon generation of specific, class-switched neutralizing antibodies. In the previous experiments, *E. coli* LPS conjugated to NP, i.e., NP-LPS, was used to exploit the function of its TLR4-engaging moiety, as all studies were aimed at analyzing the specific response to NP hapten. We exploited the intrinsic dual structure of *E. coli* LPS to coengage B cell TLR4 and BCR through its lipid A and O-antigen moieties (*42*), respectively. Thus, we vaccinated *Tcr*β*^−/−^Tcr*δ*^−/−^* mice with LPS to test the ability of this bacterial component to induce a protective response to *E. coli*. Upon infection with 1.2 × 10^8^ *E. coli* colony-forming units (CFUs), LPS-vaccinated *Tcr*β*^−/−^Tcr*δ*^−/−^* mice, but not nil (PBS)–injected controls, mounted an LPS-specific and bactericidal IgG response, displayed negligible body weight loss, showed reduced liver and blood bacterial loads, and suffered no spleen or liver tissue damage (Fig. 10, A to E).

To assess whether induction of a protective antibody response extended to TLR5-BCR linked coengagement, we vaccinated *Tcr*β*^−/−^Tcr*δ*^−/−^* mice with *S.* Typhimurium flagellin (in alum first injection, in PBS second and third injections) or nil (in alum first injection, in PBS second and third injections) and infected them with *S.* Typhimurium (10^5^ CFUs per os) (Fig. 10F). In contrast to their nil-injected controls, the flagellin-vaccinated *Tcr*β*^−/−^Tcr*δ*^−/−^* mice mounted a flagellin-specific and *S.* Typhimurium neutralizing IgG response, displayed negligible body weight loss, showed reduced bacterial loads in spleen, liver and feces, and suffered no intestinal tissue damage (Fig. 10, G to K). Last, while none of the 12 flagellin-vaccinated *Tcr*β*^−/−^Tcr*δ*^−/−^* mice showed symptoms of disease after 7 days, by the same time, 6 of the 12 nil-injected *Tcr*β*^−/−^Tcr*δ*^−/−^* mice succumbed to death by infection (Fig. 10I). Thus, TLR4-BCR and TLR5-BCR linked coengagement can induce class-switched, hypermutated (Figs. 4, 5, and 7, and figs. S8, S9, S10, S11, and S12) and neutralizing antibody responses with protective activity against pathogenic bacteria in the absence of T cells.

## DISCUSSION

It has long been held that the induction of a mature antibody response requires T cell help (*10*). CD40*^−/−^* mice reportedly failed to make class-switched, high-affinity antibodies, to form GCs, or generate memory B cells in response to T-dependent antigens (*43*). Similarly, CD154*^−/−^* mice failed to mount a mature antibody response to a conjugated hapten (*44*). They also failed to mount a specific, class-switched, neutralizing antibody response upon infection with *Citrobacter rodentium* or *S.* Typhimurium (*33*, *45*), resulting in high bacterial loads, pathology, and death. Mature antibody responses, however, have been suggested to be induced by putatively T-dependent antigens in the absence of T cells (*22*–*24*). We showed here that specific and mature antibody responses can be generated in the absence of T cells provided that the driving immunogen engages a B cell TLR in addition to the cognate BCR. Mice deficient in T cells (*Tcr*β*^−/−^Tcr*δ*^−/−^*) or deficient in T cells and virtually all other immune cells except B lymphocytes (NSG/B mice) mounted vigorous T-independent antibody responses to NP as induced by NP-LPS, to *E. coli* as induced by *E. coli* LPS, or to *S.* Typhimurium as induced by *S.* Typhimurium flagellin.

The NP-LPS–induced antibody response in T cell–deficient and NSG/B mice yielded IgGs and IgAs that bound efficiently to NP_4_, a function of high affinity. Such an antibody response depended on B cell TLR4, entailed formation of GC-like structures, leading to select B cell clonal expansion and intraclonal diversification, production of class-switched, somatically mutated antibodies, plasma cell differentiation, and generation of memory B cells supporting an anamnestic response. The NP-LPS–induced antibody response in T cell–deficient and NSG/B mice required coengagement of B cell TLR4 and BCR by physically linked TLR4 and BCR ligands—the critical role of TLR4 having been emphasized by the defective IgG3 response to NP-LPS in mixed bone marrow chimera μMT/*Tlr4^−/−^* mice. Only NP-LPS or NP–lipid A, but not NP-Ficoll admixed with LPS or LPS alone, induced NP-specific high-affinity IgG in vivo (*Tcr*β*^−/−^Tcr*δ*^−/−^* mice) and in vitro (B cells). Because of its polysaccharidic O-antigen moiety engaging the cognate BCR, LPS also inherently satisfied the physical TLR and BCR ligands’ linked requirement for TLR4-BCR coengagement, which induced both the canonical and noncanonical B cell NF-κB pathways. In NP-LPS, the polysaccharidic O-antigen did not interfere with the induction of the class-switched antibody response to NP, as NP-LPS triggered production of NP-specific IgG3 and IgG2b at levels and with kinetics comparable to those induced by NP–lipid A. A comparison of NP-specific B cells, as in B1-8 mice, with NP-specific B cells of a polyclonal response, as induced by NP-LPS in *Tcr*β*^−/−^Tcr*δ*^−/−^* mice, would further our understanding of TLR-BCR linked coengagement–mediated antibody responses.

Similar to NP-LPS, B cell TLR5-BCR linked coengagement by flagellin, a protein and critical constituent of the flagellum in nearly all flagellated bacteria, including all *Salmonellae* and most *Enterobacteriaceae*, activated the canonical and the noncanonical NF-κB pathways, both important for induction of *Aicda* and *Prdm1* expression. Flagellin, which has been thought to “absolutely” require T cell help for induction of a specific antibody response (*31*–*33*, *46*), in fact induced in our hands specific, class-switched, somatically mutated IgG3, IgG1, IgG2b, and IgA antibodies together with plasma cell differentiation and an anamnestic antibody response in *Tcr*β*^−/−^Tcr*δ*^−/−^* and NSG/B mice. The intrinsic B cell TLR5-BCR linked coengagement mechanism would not only effectively induce production of specific antibodies at early stages of *Salmonella* infection when T cell help is not yet available but would also potentiate the antibody response at later stages when T cell help becomes available, as exemplified by an accurate comparison of the anti-flagellin antibody response in C57BL/6 and *Tcr*β*^−/−^Tcr*δ*^−/−^* mice. A similar relevance to both early and late stages of the antibody response would also likely apply to TLR4-BCR linked coengagement. Thus, B cell TLR4-BCR and TLR5-BCR linked coengagement would function as early-stage drivers and late-stage potentiators in the maturation of antibody responses to bacterial and viral components.

Naïve B cells have been suggested to respond to engagement of TLR4 and other TLR ligands, as well as BCR cross-linking by up-regulating TLR expression (*47*–*50*). Building on our previous observations (*15*, *24*, *25*), we showed here at single-cell level that naïve mouse and human B cells express surface TLR4 and TLR5, albeit at a relatively low density. These B cells significantly increased both TLR4 and TLR5 expression in response to either NP-LPS or flagellin, reflecting the engagement of not only TLR4 or TLR5 but also, possibly, that of TLR4/TLR5 heterodimers. TLR4 and TLR5 receptors exist as individual TLR4 and TLR5 homodimers and TLR4/TLR5 heterodimers (*46*). In addition to TLR-BCR linked coengagement, the cytokines added to B cell cultures to direct CSR might have played a role in increasing B cell TLR expression. TLR4/TLR5 heterodimers (*36*, *51*) may extend TLR4 involvement in the induction of antibodies to microbial proteins, such as respiratory syncytial virus F protein, Dengue virus NS1 protein, *Chlamydia pneumoniae* HSP60, and *Flavobacterium meningosepticum* flavolipin (*52*–*55*). Increased B cell TLR expression paralleled plasma cell differentiation and would boost secretion of IgG and/or IgA upon further TLR stimulation (*56*, *57*). Thus, up-regulation of TLR4 and/or TLR5 on plasmablasts and plasma cells, as induced by bacterial components’ TLR4-BCR or TLR5-BCR linked coengagements, could lead to a further bacteria-induced boost of antibody production, as expression of an antibacterial self-amplifying loop of defense. This would be particularly relevant to body districts with abundant microbial commensals and pathogens, such as the gut. There, the putative B cell TLR-BCR linked coengagement–dependent antibacterial defense loop could synergize with parietal lumen TLR4 and TLR5, as induced by RA, a metabolite of vitamin A that is abundant in the intestine, leading to improved intestinal barrier function (*58*, *59*).

The T-independent class-switched, high-affinity IgG antibody response to NP induced by NP-LPS in *Tcr*β*^−/−^Tcr*δ*^−/−^* mice lent itself to an in-depth analysis, as the gene encoding the NP-binding V_H_ segment (V1-72) is well characterized (*60*, *61*). V1-72DJ_H_-Cγ3 and V1-72DJ_H_-Cγ2b transcripts with identical CDR3 and J_H_ sequences allowed for identification of a few dominant and intermediate NP-specific B cell clones together with several small clones and many B cell microclones in NP-LPS–immunized *Tcr*β*^−/−^Tcr*δ*^−/−^* mice. The long IgH CDR3s of these transcripts were evocative of the long IgH CDR3s of the antibodies elicited by naturally occurring antigens in humans (*62*–*65*). In *Tcr*β*^−/−^Tcr*δ*^−/−^* mice, the NP-specific B cell clonal and intraclonal diversification, which led to emergence of high-affinity antibodies, likely reflected a stepwise SHM and clonal selection process similar to that underpinning the maturation of the T-dependent anti-NP V1-72DJ_H_-Cγ1 response elicited by NP-CGG in C57BL/6 mice. In such mice responding to NP, V1-72 point mutations included, in addition to the critical W33L replacement, the M34I, K59R, and S66N replacements, which together with W33L increase antibody affinity for NP by up to 100-fold (*60*, *61*, *66*, *67*). These mutations, which generally occur in the secondary T-dependent antibody response to NP, as conjugated with a carrier protein, are important for V1-72 segment recognition of NP, particularly when V1-72 is paired with a λ1 light chain (*68*, *69*), and have also been associated with the specific memory B cell response to this hapten (*61*). To rule out that these critical point mutations, as introduced by AID in *Tcr*β*^−/−^Tcr*δ*^−/−^* B cells, might have been randomly conserved outside of a putative selection process would require a detailed analysis of the temporally sequential accumulation of affinity-gaining point mutations, an endeavor beyond the scope of this work. Similar to *Tcr*β*^−/−^Tcr*δ*^−/−^* mice responding to NP-LPS, *Tcr*β*^−/−^Tcr*δ*^−/−^* mice responded to flagellin with SHM/CSR, selective B cell clonal expansion, and intraclonal diversification. The V_H_1DJ_H_-Cγ3 and V_H_1DJ_H_-Cγ2b antibody mutational loads of the anti-flagellin response in *Tcr*β*^−/−^Tcr*δ*^−/−^* mice were significantly greater than those of the antibody responses induced by NP-LPS in similar mice and in C57BL/6 mice and the mutational load of the antibody response induced by NP-CGG in C57BL/6 mice, a contrast whose causal root was difficult to discern. However, flagellin-immunized *Tcr*β*^−/−^Tcr*δ*^−/−^* mice displayed a significantly higher frequency of point mutations in V_H_1DJ_H_-Cγ3 and V_H_1DJ_H_-Cγ2b transcripts (overall: 2.4 ± 0.17 × 10^−2^ change per base, means ± SEM) than that in V_H_1DJ_H_-Cγ (all subclasses) (overall: 1.4 ± 0.43 × 10^−2^ change per base, *P* = 0.035) of BALB/c mice infected with *S.* Typhimurium (*41*) and significantly higher than the mutation frequencies in V1-72DJ_H_-Cγ1 transcripts of C57BL/6 mice responding to NP-CGG (2.6 ± 0.03 × 10^−3^ change per base, *P* < 0.001), possibly as a result of the lack of T_reg_ B cells and their modulatory activity in *Tcr*β*^−/−^Tcr*δ*^−/−^* mice. In terms of the T-independent IgA response, flagellin-immunized *Tcr*β*^−/−^Tcr*δ*^−/−^* mice displayed a frequency of point mutations in V_H_1DJ_H_-Cα transcripts (1.1 ± 0.3 × 10^−2^ change per base), which was high yet lower (2.4 ± 0.15 × 10^−2^ change per base, *P* = 0.004) that that in mice responding to *S.* Typhimurium infection (*41*). This is consistent with the well-known efficient induction of IgAs by a replicating intestinal bacterium in an active phase of infection.

SHM/CSR, intraclonal diversification and selection have long been thought to be dependent on T cell help and GC formation (*11*, *12*). Formation of GC-like structures, however, has been suggested to occur in C3H/HeN mice in response to T-independent 2,4-dinitrophenyl (DNP)–LPS and DNP-Ficoll (*70*), in quasi-monoclonal mice in response to T-independent NP-Ficoll (*71*), and in *Tcr*β*^−/−^Tcr*δ*^−/−^* mice in response to polyoma virus (*19*), Aβ liposomes (*20*), and bacteriophage Qβ-VLPs (*21*) but not in *Tcr*β*^−/−^Tcr*δ*^−/−^* mice injected with NP-LPS (*72*). Although GCs have long been regarded as the sites of SHM/CSR, SHM/CSR could occur extrafollicularly in mice infected with *Salmonella*, *Ehrlichia muris*, or other bacteria and viruses, as well as in humans with systemic lupus erythematosus and lupic mice (*73*–*76*). While we did not investigate the formation of extrafollicular structures, we readily identified distinct GC-like structures involving B220^+^PNA^+^ and GL7^+^Fas^+^ B cells (high avidity for PNA and high surface expression of Fas are characteristic of GC B cells) in *Tcr*β*^−/−^Tcr*δ*^−/−^* mice responding to NP-LPS and *S.* Typhimurium flagellin. These T-independent GC-like structures resembled those induced by T-dependent NP-CGG in C57BL/6 mice. Their generation and maintenance deserve further characterization, which will be the subject of future investigations. In *Tcr*β*^−/−^Tcr*δ*^−/−^* mice, GC-like structures were likely instrumental in B cell SHM/CSR, clonal expansion, selection, and intraclonal diversification, leading to the generation of high-affinity NP-specific IgG3 and IgG2b ASCs as well as flagellin-specific IgG3, IgG1, IgG2b, and IgA ASCs. Isolating single GC-like B cells and sequencing their BCR genes for analysis of SHM/CSR would provide an additional dimension to our studies.

It has long been held that T-independent responses, such as those induced by bacterial capsular polysaccharides, hardly lead to generation of memory B cells. Rather, B cells engaged by T-independent antigens would rapidly undergo differentiation to plasma cells, which would persist for some time as secretors of antibodies (*77*). Nevertheless, in NP-LPS–injected *Tcr*β*^−/−^Tcr*δ*^−/−^* mice, IgG^+^ memory B cells emerged at a high frequency, with a greater proportion of IgG3 than IgG2b, and supported an anamnestic high-affinity IgG response to NP. These memory B cells differed from Ficoll-induced “memory” B cells, which are B1 derived and only make IgM (*17*). They also differed from the seemingly T-independent memory B cells generated in response to *Borrelia hermsii*, *Streptococcus pneumoniae*, and *Francisella tularensis* (*78*, *79*), which also display a naïve B1 cell–like phenotype and mediate only borderline recall responses (*18*). Thus, the NP-LPS–induced memory response in *Tcr*β*^−/−^Tcr*δ*^−/−^* mice further questions the long-held notion that the generation of memory B cells critically requires T cell help (*8*, *80*, *81*), as further emphasized by the anamnestic antibody response, particularly IgA, to flagellin elicited in *Tcr*β*^−/−^Tcr*δ*^−/−^* mice and the long-lasting class-switched anamnestic response to NP induced by NP-LPS in BALB/c mice, be they young or old.

Gram-positive bacteria (T-independent) capsular polysaccharides, as in pneumococcal polysaccharide vaccines, induce mainly IgM and a poor or limited antibody memory response upon revaccination or following infection (*82*–*84*), possibly because of these polysaccharides’ inability to induce a “second signal” in B cells (*85*). Our experiments showed that a B cell–intrinsic second signal can be efficiently provided by engagement of a B cell TLR, the “first signal” being provided by cognate BCR cross-linking. As we showed in *E. coli*, a second signal was provided by TLR4-engaging LPS lipid A moiety to complement the first signal, as provided by BCR-engaging LPS O-antigen moiety. LPS inherently satisfied the physical requirement for TLR4-BCR linked coengagement and induced a neutralizing antibody response with protective activity against *E. coli* in *Tcr*β*^−/−^Tcr*δ*^−/−^* mice; in similar mice injected with NP-LPS, the first signal was provided by NP, leading to production of high-affinity class-switched anti-NP antibodies. The critical role of TLR4 engagement as a second signal in induction of a mature antibody response was shown by lipid A moiety of LPS as conjugated to NP (NP–lipid A). NP–lipid A induced a NP-specific high-affinity IgG3 and IgG2b antibody response comparable to that induced by NP-LPS. To the best of our knowledge, bacterial polysaccharides, such as the Pneumococcus capsular polysaccharide, do not engage any known TLRs, thereby limiting their inherent ability to induce class-switched, mature, and fully effective antibody responses.

Our vaccinations of *Tcr*β*^−/−^Tcr*δ*^−/−^* mice with *E. coli* LPS and *S.* Typhimurium flagellin led, to the best of our knowledge, the first demonstration of induction of class-switched, neutralizing, and protective antibodies against live bacteria in the absence of T cells. Similar to *E. coli* LPS, *S.* Typhimurium flagellin induced high levels of bacteria neutralizing antibodies, reduced systemic and tissue bacterial burden, tempered pathology, prevented body weight loss, and promoted survival in *Tcr*β*^−/−^Tcr*δ*^−/−^* mice. The T-independent, B cell–intrinsic, protective responses to *E. coli* and *S.* Typhimurium outlined here are relevant to the modalities of human antibody responses to other flagellated and non-flagellated microbial pathogens and, possibly, certain viruses, particularly enveloped viruses, such as influenza, measles, human respiratory syncytial virus, or severe acute respiratory syndrome coronavirus 2 (SARS-CoV-2). The requirement of T cells to support the generation of an effective anti-viral antibody has been questioned by the finding that follicular T helper (T_FH_) cell–deficient mice made neutralizing antibodies in response to SARS-CoV-2 infection, SARS-CoV-2 vaccination, and influenza A virus infection (*86*). The limited frequency of mutations in these antibodies, however, contrasted with the heavy mutational load and extensive intraclonal diversification of our flagellin- and LPS-driven class-switched antibody responses.

Intrinsic B cell TLR-BCR linked coengagement would be important in induction of mature antibody responses to enteric bacteria, such as *E. coli* and *Salmonella*, and non-enteric bacteria, such as the ubiquitous *Staphylococcus aureus* or respiratory infection bacteria, such as *S. pneumoniae* and *Haemophilus influenzae*. It may provide a source of protective antibodies in settings of T cell impairment, as in certain patients with coronavirus disease 2019 (COVID-19) (*86*) or subjects with HIV-acquired T cell immunodeficiency (*87*). It would also account for the ability of T cell–incompetent children and young adults to cope with many bacterial infections, as in subjects with partial DiGeorge syndrome (thymic hypoplasia), in whom no correlation occurs between serum IgG levels and numbers of circulating T_FH_ cells (*88*). CD154- and CD40-deficient patients display mainly defects in T cell–mediated immunity to viruses and fungi, particularly *Pneumocystis carinii* and *Cryptosporidium parvum* (*89*). These patients’ B cells have no obvious functional defect and can mount class-switched antibody responses given appropriate stimuli (*89*). B cell TLR deficiencies, such as TLR4, however, lead to reduced antibody responses to bacterial and viral components, including those that elicit T-dependent antibody responses, possibly emphasizing the contribution of intrinsic B cell TLR-BCR linked coengagement to antimicrobial defense (*90*–*92*).

TLR ligands have been used in subunit vaccines to enhance antibody responses to T-dependent antigens (*35*). Our findings that TLR4-BCR and TLR5-BCR linked coengagement induce *Aicda/AICDA* and *Prdm1/PRDM1* for SHM/CSR as well as plasma cell and memory B cell differentiation redefine these TLR ligands as critical stimuli for induction of mature antibody responses, rather than mere “adjuvants,” as widely assumed (*36*, *93*). TLR-BCR linked coengagement can substitute for CD154:CD40 engagement in the induction of a mature and protective antibody response when T cell help is not available. As we have shown here and elsewhere (*24*, *25*), both TLR-BCR linked coengagement and CD154:CD40 engagement activate the B cell canonical (mediated through TLR-MyD88 in TLR signaling) and noncanonical (mediated through BCR–phosphatidylinositol 3-kinase signaling in TLR-BCR linked coengagement) NF-κB pathways, indicating a signaling commonality in induction of *Aicda* and *Prdm1*, and, therefore, T-independent and T-dependent AID and Blimp-1 expression (*94*–*96*). The role of TLR-BCR linked coengagement as a driver of mature antibody responses possibly extends beyond TLR4 and TLR5 to other B cell TLRs, such as TLR2, TLR6, TLR7, TLR9, and TLR11, as suggested by the data by us and others (*24*, *25*, *36*, *47*, *97*). Intrinsic B cell TLR-BCR linked coengagement likely plays an important role in inducing specific, mature, and neutralizing antibody responses in the absence of T cells. It would also play a role in early stages of T-dependent antimicrobial responses when T cell help is not yet available and potentiate the late-stage response when T cell help is fully available. Last, it may inform the development of B cell TLR-based vaccines to microbial pathogens, particularly for subjects with an immature T cell compartment, such as the infant, or declining T cell function and restricted clonal composition, such as the elderly.

## MATERIALS AND METHODS

### Mice

C57BL/6 (stock #000664), *Tcr*β*^−/−^Tcr*δ*^−/−^* (B6.129P–*Tcr*β*^−/−^ Tcr*δ*^−/−^;* stock #002121), B6.129S2-*Ighm^tm1Cgn^*/J (stock #002288), B6.B10ScN-*Tlr4^lps-del^*/JthJ (stock #007227), and NSG (NOD.Cg *Prkdc^scid^Il2rg^tm1Wjl^*/SzJ; stock #005557) mice were purchased from the Jackson Laboratory (Bar Harbor, Maine). BALB/c mice (strain code #028) were purchased from Charles River Laboratories. Male and female mice used in all experiments were 8 to 12 weeks of age. All mice were free of infection or apparent disease and were housed in the University of Texas (UT) Health San Antonio pathogen-free animal vivarium. All protocols were in accordance with the regulations of the UT Health San Antonio Institutional Animal Care and Use Committee.

NSG mice have no B cells, T cells and NK cells and have low numbers of functionally defective DCs and monocytes. NSG/B mice were constructed by grafting NSG mice with B cells purified from the spleen of 8- to 12-week-old C57BL/6 mice by negative selection using the EasySep Mouse B Cell Isolation Kit (19854, STEMCELL Technologies) following the manufacturer’s instructions. Purified B cells were injected intravenously (2.0 × 10^7^ cells per mouse in 250 μl of PBS in lateral tail veins) into 8- to 12-week-old NSG mice. After B cell engraftment, mice were injected intraperitoneally with NP-LPS (100 μg in 100 μl of PBS) or purified *S.* Typhimurium flagellin at days 0 (50 μg in 100 μl of alum, Imject Alum, Thermo Fisher Scientific), 2, and 4 (50 μg in 100 μl of PBS) and euthanized at day 14.

*Tg(Aicda-cre)Rosa26^fl-STOP-fl-Luc^* mice and their wild-type (WT) littermate controls were generated by crossing B6.129P2-*Aicda^tm1(cre)Mnz^*/J (stock #007770, the Jackson Laboratory) with *Rosa26^fl-STOP-fl-Luc^* mice (stock #034320, the Jackson Laboratory) as generated by a customized TurboKnockout approach (Cyagen Biosciences). Briefly, *Tg(Aicda-cre)* mice express Cre recombinase from a bacterial artificial chromosome transgene harboring an *Aicda* locus complete of its promoter. Crosses of this strain with Cre-reporter mice (e.g., *Rosa26^fl-STOP-fl-Luc^* mice) lead to recombinase activity, which results in the deletion of the loxP site-flanked “STOP” cassette in the *Rosa26* locus of activated B cells. This enables luciferase expression of this subset of B cells, which can be detected in vivo and in vitro via bioluminescent imaging.

NSG *Luc B-Tg* mice were constructed by injecting purified *Tg(Aicda-cre)Rosa26^fl-STOP-fl-Luc^*, *Rosa26^fl-STOP-fl-Luc^*, and *Tg(Aicda-cre)Rosa26^+/+^* B cells into NSG mice. Naïve IgM^+^IgD^+^ B cells were purified from the spleen of 8- to 12-week-old *Tg(Aicda-cre)* *Rosa26^fl-STOP-fl-Luc^*, *Rosa26^fl-STOP-fl-Luc^*, and *Tg(Aicda-cre)Rosa26^+/+^* mice by negative selection using the EasySep Mouse B Cell Isolation Kit (19854, STEMCELL Technologies) following the manufacturer’s instructions and supplemented with additional anti-CD3 monoclonal antibody (mAb) (clone 17A2, BioLegend), resulting in more than 99% IgM^+^IgD^+^ B cells. B cells were injected intravenously (2.0 × 10^7^ cells per mouse in 250 μl of PBS in lateral tail veins) into 8- to 12-week-old NSG mice. After B cell engraftment, mice were injected intraperitoneally with NP-LPS (100 μg in 100 μl of PBS) at days 0, 2, and 4 or purified *S.* Typhimurium flagellin at days 0 (50 μg in 100 μl of alum), 2, and 4 (50 μg in 100 μl of PBS) and euthanized at day 14.

Mixed bone marrow chimeric μMT*/Tlr4^+/+^* and μMT*/Tlr4^−/−^* mice were constructed using age- and sex-matched recipient C57BL/6 mice from the same breeding batch by the Jackson Laboratory. They were treated with neomycin sulfate in drinking water (2 mg/ml) for 1 week before γ-irradiation (1000 cGy within 10 min) using a cesium source. After 24 hours, the mice were randomized into two groups of six mice each and received, through tail vein injection, 2.5 × 10^6^ mixed bone marrow cells containing 2 × 10^6^ (i.e., 80%) cells from a single μMT mouse and 0.5 × 10^6^ (20%) cells from a C57BL/6 or *Tlr4*^−/−^ mouse. Before mixing, bone marrow cells isolated from each donor mouse were depleted of T cells by incubation with biotinylated anti-CD3 mAb and Magnisort Streptavidin Negative Selection Beads (eBioscience). μMT*/Tlr4^+/+^* and μMT*/Tlr4^−/−^* chimeric mice were maintained for at least 8 weeks to allow for reconstitution of their peripheral lymphoid system, as confirmed by flow cytometry for CD19^+^, CD3^+^, CD11b^+^, CD11c^+^, and/or Gr1^+^ cells (by specific fluorophore mAbs; table S8) and the presence or absence of TLR4^+^ B cells isolated from peripheral blood (100 μl).

### Immunization of mice with NP-LPS, LPS, NP–lipid A, NP-CGG, NP-Ficoll, or flagellin

C57BL/6, BALB/c, *Tcr*β*^−/−^Tcr*δ*^−/−^*, NSG/B, and/or mixed bone marrow chimeric μMT*/Tlr4^+/+^* and μMT*/Tlr4^−/−^* mice were injected intraperitoneally with PBS (100 μl), NP-LPS [NP conjugated to *E. coli* LPS; average: 0.4 NP molecule conjugated with one LPS molecule; Biosearch Technologies; 25 μg in 100 μl of PBS (Fig. 2, A and B) or 100 μg of NP-LPS in 100 μl of PBS for all other experiments], LPS deproteinized by chloroform extraction (from *E. coli* serotype 055:B5, Sigma-Aldrich; 25 μg in 100 μl of PBS), NP–lipid A (1 NP-chloride molecule covalently conjugated with one free *E. coli* LPS lipid A molecule; 25 μg in 100 μl of PBS), NP-CGG (average: 16 NP molecules conjugated with one CGG molecule; Biosearch; 100 μg in 100 μl of PBS), NP_26_-Ficoll (average: 26 NP molecules conjugated with one Ficoll molecule; Biosearch; 25 μg in 100 μl of PBS), alum (100 μl; Imject Alum Adjuvant, Thermo Fisher Scientific) alone as “nil” control, *S.* Typhimurium flagellin (CVD1925 FliC), 50 μg in 100 μl of alum for the first injection, or 50 μg in 100 μl of PBS for any following injection. Sera were collected before injection and at specified time points after injection.

### Bioluminescence imaging of *Tg(Aicda-cre)Rosa26^fl-STOP-fl-luc^* and NSG *Luc B-Tg* mice

To characterize the *Tg(Aicda-cre)Rosa26^fl-STOP-fl-Luc^* mouse model, a preliminary study was performed with four *Rosa26^fl-STOP-fl-Luc^* and four *Tg(Aicda-cre)Rosa26^fl-STOP-fl-Luc^* mice to determine the optimal imaging parameters using the IVIS 100 Imaging System (Caliper Life Sciences Inc. Hopkinton, MA, USA). Briefly, mice were anesthetized with isoflurane (1.5 to 3.0%) and injected intraperitoneally in the lower left abdominal quadrant with luciferin (150 mg/kg; Caliper Life Sciences Inc.) suspended in Dulbeccos’s PBS (15 mg/ml). The mouse abdomen was shaven to minimize light scattering. To determine the time frame for optimal luciferase activity, mice were imaged for 2 to 3 min at 5, 10, 15, and 20 min after luciferin injection. The optimal signal intensity was detected approximately 10 min after luciferin injection. Following imaging optimization, *Tg(Aicda-cre)Rosa26^fl-STOP-fl-Luc^* mice and NSG *Luc B-Tg* mice were injected intraperitoneally with NP-LPS (100 μg in 100 μl of PBS) or *S.* Typhimurium flagellin (50 μg in 100 μl of alum) to induce AID expression, followed by luciferase expression in B cells. Fourteen days after injection with NP-LPS or flagellin, these mice were imaged ventral side up, and organs were harvested and imaged using the optimized parameters determined from the preliminary study. All images were analyzed using Living Image 2.50 software (Caliper Life Sciences Inc., Hopkinton, MA, USA).

### Specific ELISAs

To determine titers of total IgM, IgG3, IgG1, IgG2a, IgG2b, and IgA, sera from injected mice were first diluted 1000-fold with PBS (pH 7.4) containing 0.05% Tween 20 (v/v) (PBS–Tween 20). Twofold serially diluted samples and standards for each Ig isotype were incubated in 96-well plates coated with pre-adsorbed goat anti-mouse IgM, goat anti-mouse IgA, or goat anti-mouse IgG (IgG3, IgG1, IgG2a, and IgG2b) antibodies (Abs) (1 μg/ml in 0.1 M sodium carbonate/bicarbonate buffer pH 9.6). After washing with PBS-Tween 20, captured Igs were detected with biotinylated goat anti-mouse IgM, IgG3, IgG1, IgG2a, IgG2b, or IgA mAbs followed by horseradish peroxidase (HRP)–labeled streptavidin (Sigma-Aldrich), *o*-phenylenediamine (OPD) substrate, and measurement of converted substrate absorbance at 492 nm. Optical density (O.D.) values were processed using GraphPad Prism 9.4.1 software or Excel (Microsoft) software to plotted curves and histograms. To analyze titers of specific antibodies (high-affinity anti-NP_4_ Abs, anti-flagellin Abs, and anti-LPS Abs), sera were diluted 100-fold in PBS–Tween 20. Twofold serially diluted samples were incubated in a 96-well plate coated with NP_4_–bovine serum albumin (BSA) (average four NP molecules per one BSA molecule, 1 μg/ml; Biosearch) or BSA (1 μg/ml; Biosearch) as a control, *S.* Typhimurium flagellin (1 μg/ml), or *E. coli* LPS (1 μg/ml). At such low coating concentration, only 1 to 3% of NP_4_-BSA remains bound to plastic wells. This results in a functional monovalency of NP_4_-binding antibodies, one that requires an IgG with a Fab of high-intrinsic affinity to bind to NP_4_ (*28*, *29*). Captured Igs were detected with biotinylated goat anti-mouse IgM, IgG3, IgG1, IgG2a, IgG2b, or IgA mAbs, followed by HRP-labeled streptavidin and OPD substrate. Hapten and antigen-specific antibody titers were expressed as relative units (RUs), defined as the dilution factor needed to reach 50% saturation binding, as calculated using GraphPad Prism software.

### B cells, in vitro CSR, and plasma cell differentiation

Mouse B cells were isolated from single-cell suspensions prepared from the spleen, lymph nodes (pooled from inguinal, axillary, brachial, and cervical lymph nodes), bone marrow (from tibia and femur), or peripheral blood (collected by submandibular bleeding) of 8- to 12-week-old C57BL/6 mice and *Tcr*β*^−/−^Tcr*δ*^−/−^* mice. Erythrocytes were lysed by ammonium-chloride-potassium lysis buffer (Lonza). Erythrocyte-free cells were resuspended in fetal calf serum (FCS)–RPMI [Invitrogen RPMI-1640 medium supplemented with 10% (v/v) Hyclone FCS], penicillin-streptomycin [1% (v/v), Invitrogen], and amphotericin B [1% (v/v), Invitrogen]. Naïve B cells were isolated by negative selection (anti-CD43, CD4, CD8, CD11b, CD49b, CD90.2, Gr-1, or Ter119 mAbs) using the EasySep Mouse B cell Isolation Kit (19854, STEMCELL Technologies) following the manufacturer’s instructions, yielding approximately 99% naïve IgM^+^IgD^+^ B cells. After pelleting, these B cells were resuspended in FCS-RPMI before analysis or in vitro stimulation and culture. For CSR induction and plasma cell differentiation, mouse naïve IgM^+^IgD^+^ B cells (seeded at 10^6^ cells per well in triplicate or quadruplicate) were stimulated with CD154 (3 U/ml), LPS (3 μg/ml), NP-LPS (3 μg/ml), NP-Ficoll (3 μg/ml), or *S.* Typhimurium flagellin (10 μg/ml) plus recombinant IL-4 (3 ng/ml; R&D Systems) for CSR to IgG1 or TGF-β (4 ng/ml, R&D Systems) plus RA (4 ng/ml) for CSR to IgA and cultured in FCS-RPMI for 48 hours for mRNA transcript analysis and 96 hours for fluorescence-activated cell sorting (FACS) analysis (including flow imaging) (*2*–*4*).

Human naïve IgM^+^IgD^+^ B cells (~99%) were purified by negative selection from peripheral blood mononuclear cells obtained from healthy subject buffy coat (South Texas Blood & Tissue Center, San Antonio) using the EasySep Human Naïve B Cell Enrichment Kit (19254, STEMCELL Technologies) following the manufacturer’s protocol. For CSR induction and plasma cell differentiation, human naïve IgM^+^IgD^+^ B cells (seeded at 10^6^ cells per well in triplicate or quadruplicate) were stimulated with NP-LPS (3 μg/ml) or *S.* Typhimurium flagellin (10 μg/ml) plus huIL-2 (100 ng/ml), huIL-4 (20 ng/ml), and huIL-21 (50 ng/ml) for CSR to IgG and cultured in FCS-RPMI for 72 hours for mRNA transcript analysis and 96 hours for FACS analysis (including flow imaging) (*2*–*4*).

### Flow cytometry

For FACS analysis of surface markers, in vitro stimulated B cells were stained with fluorochrome-conjugated mAbs in Hank’s balanced salt solution plus 0.1% BSA (BSA-HBSS) for 20 min. Ex vivo B cells and other immune cells from the spleen, lymph node, Peyer’s patches, or circulating blood (of C57BL/6 and *Tcr*β*^−/−^Tcr*δ*^−/−^* mice injected with NP-LPS, NP-CGG, or *S.* Typhimurium flagellin) were stained with fluorochrome-conjugated mAbs in the presence of mAb clone 2.4G2 (BD Biosciences), which blocked FcγIIIR and FcγIIR, for 20 min. After washing, cells were resuspended in BSA-HBSS. In vitro stimulated and ex vivo cells were stained with Pacific Blue–anti-CD19 mAb (115523, BioLegend), phycoerythrin (PE)–Cy7–anti-CD138 mAb (142513, BioLegend), PE-Cy7–anti-CD38 mAb (102718, BioLegend), allophycocyanin (APC)–anti-IgD mAb (405714, BioLegend), peridinin-chlorophyll-protein (PerCP)/Cyanine5.5 anti-IgD mAb (405709, BioLegend), fluorescein isothiocyanate (FITC)–anti-IgM mAb (406506, BioLegend), PE–anti-IgM mAb (406507, BioLegend), APC–anti-IgM mAb (406509, BioLegend), BV605–anti-IgM mAb (406523, BioLegend), Alexa Fluor 647 –anti-IgG polyclonal Ab (pAb) (405322, BioLegend), APC–anti-IgG1 mAb (406609, BioLegend), BV421–anti-IgG3 mAb (565808, BD Biosciences), FITC–anti-IgG2b mAb (406705, BioLegend), APC–anti-IgG2a mAb (407110, BioLegend), PE–anti-IgA mAb (12-4204-81, eBiosciences), APC–anti-IgA mAb (17-4204-82, eBiosciences), APC–anti-CD284 (TLR4) mAb (145405, BioLegend), PE–anti-CD285 (TLR5) mAb (148107, BioLegend), PerCP-Cy5.5-GL7 mAb (144609, BioLegend), Alexa Fluor 647–anti-GL7 mAb (144605, BioLegend), and/or BV510–anti-CD95 (Fas) (563646, BD Biosciences) and 7-amino-actinomycin D (A9400, Sigma-Aldrich). NP-specific B cells were stained with NP_23_-PE (1 mg/ml; Biosearch) or PE alone (52412-1MG-F, Sigma-Aldrich) as a negative control; PE^+^ B cells were at most 0.5%. Monocytes/macrophages were profiled with APC–anti-CD11b mAb (101212, BioLegend), DCs with APC-Cy7–anti-CD11c mAb (117323, BioLegend), and T cells with Pacific Blue–anti-CD3 mAb (100214, BioLegend) (table S8). Human B cells were stained with PE-Cy7–anti-human-CD19 mAb (60-0199, Tonbo Biosciences), BV510–anti-human-CD138 mAb (356518, BioLegend), PE–anti-human-CD284 (TLR4) mAb (312805, BioLegend), APC–anti-human-CD285 (TLR5) mAb (394507, BioLegend), PE–anti-human-CD27 mAb (356405, BioLegend), BV421–anti-human-IgM mAb (314516, BioLegend), PerCP–anti-human IgD mAb (348233, BioLegend), FITC–anti-human IgG mAb (555786, BD Pharmingen), APC–anti-human IgA mAb (130-116-879, Miltenyi Biotec), and fixable viability dye eFluor 780 (65-0865-14, eBiosciences) (table S9).

For FACS analysis of Bcl6-, AID-, and Blimp-1–expressing mouse B cells and IgG- and IgA-secreting mouse B cells, plasmablasts, and plasma cells, 5 × 10^6^ cells were first surface stained in the presence of 2.4G2 mAb FcγIIIR and FcγIIR blocker and fixable viability dye eFluor 506 (65-0866-14, eBiosciences) (table S8). After washing, cells were fixed by resuspension in BD Cytofix/Cytoperm buffer (250 μl; 554655, BD Biosciences) and incubation at 4°C for 20 min. After washing twice with BD Perm/Wash buffer (554723, BD Biosciences) for permeabilization, cells were stained with PE–anti–Bcl-6 mAb (358503, BioLegend), Alexa Fluor 647–anti-AID pAb (bs-7855R, Bioss Antibodies), PE–anti–Blimp-1 mAb (150005, BioLegend), FITC–anti-IgG pAb (406001, BioLegend), BV421–anti-IgG3 mAb, APC–anti-IgG1 mAb, FITC–anti-IgG2b mAb, APC–anti-IgG2a mAb, or FITC–anti-IgA mAb (11–4204-82, eBiosciences) in BD Perm/Wash buffer for 30 min at 4°C. After washing again twice with BD Perm/Wash buffer, cells were then resuspended in BSA-HBSS for flow cytometry. For FACS analysis of IgG-secreting human B cells, plasmablasts, and plasma cells, 5 × 10^6^ cells were first surface stained as described and with fixable viability dye eFluor 780 (65-0865-14, eBiosciences). After washing, cells were fixed/permeabilized as described and stained with FITC–anti-human IgG mAb (table S9) in BD Perm/Wash buffer for 30 min at 4°C. Cells were washed again twice with BD Perm/Wash buffer and then resuspended in BSA-HBSS for analysis.

In all experiments, FACS analysis was performed on single-cell suspensions. Cells were appropriately gated on forward and side scattering to exclude dead cells and debris. Samples were run in a BD LSRII or BD FACSCelesta flow cytometer (BD Biosciences) with FACSDiva software (BD Biosciences), and data were acquired and analyzed using FlowJo 10.3 software (TreeStar Inc.).

### Flow imaging

For CD19, IgM, TLR4, CD138, TLR5, IgG and IgA imaging of single cells, freshly isolated naïve C57BL/6 mouse and human B cells were stimulated in vitro with NP_0.4_-LPS or flagellin and cultured for 96 hours – nil denoted unstimulated and noncultured B cells. After culture, cells were stained as described and fixed in BD Cytofix/Cytoperm buffer for 20 min at 4°C. After washing, fixed cells were resuspended in 0.1% BSA-HBSS. Cells were imaged (80x magnification) using an Amnis ImageStream X-MKII multispectral imaging flow cytometer (Luminex Corp.); compensation of fluorescence signals was performed using single color cell samples. For each group of experimental sample, 200 to 800 cells of interest were acquired according to Luminex Amnis protocol. Data were elaborated using IDEAS 6.2 image analysis software (Luminex Amnis).

Surface TLR4 and TLR5 expression on mouse and human B cells and plasmablasts was quantified using ImageJ (U.S. National Institutes of Health) (table S7). A region of interest was drawn over images of naïve B cells and plasmablasts and analyzed to measure pixel density. Surface TLR4 and TLR5 fold expression was calculated in mouse and human plasmablasts as the pixel density of these cells divided by the pixel density of naïve B cells.

### Hematoxylin and eosin staining and immunofluorescence microscopy

To analyze formation of GC-like structures, C57BL/6 and *Tcr*β*^−/−^Tcr*δ*^−/−^* mice spleens were collected at 10 days after injection with NP-CGG and NP-LPS. To analyze pathology, livers and intestines were collected from vaccinated, nonvaccinated, infected, and noninfected *Tcr*β*^−/−^Tcr*δ*^−/−^* mice, and intestines were folded into “Swiss rolls.” Spleens, intestines, and livers were fixed with paraformaldehyde (4%), embedded in paraffin, sectioned, and stained with hematoxylin and eosin. Images were captured using a Zeiss Imager V.1 (5× or 10× objective). For immunofluorescence microscopy of GC-like structures, C57BL/6 and *Tcr*β*^−/−^Tcr*δ*^−/−^* mice spleens were snap frozen in Tissue-Tek O.C.T. Compound (4583, Sakura). Spleen sections (6 μm) were prepared by cryostat and loaded onto positively charged slides, fixed in cold acetone, and stained with Alexa Fluor 647–anti-GL7 mAb (144605, BioLegend) and FITC–anti-B220 mAb (103206, BioLegend) or Alexa Fluor 488 Lectin PNA (L21409, Thermo Fisher Scientific) and PE–anti-B220 mAb (50–0452, TONBO Bioscience), respectively, for 1 hour at 25°C in a moist chamber. Cover slips were then mounted using ProLong Gold Antifade Mountant with 4′,6-diamidino-2-phenylindole (DAPI) for fluorescence microscopy. Fluorescence images were captured using a Zeiss Axio Imager Z1 fluorescence microscope (20× objective).

### Antibody-secreting cells

To identify and enumerate ASCs (plasmablasts and plasma cells), the spleen and bone marrow cells isolated from C57BL/6, *Tcr*β*^−/−^Tcr*δ*^−/−^*, and NSG/B mice or 96 hour-cultured B cells were first suspended in FCS-RPMI supplemented with 50 μM β-mercaptoethanol and then plated on 96-well polyvinylidene difluoride multi-screen filter plates (100 μl per well; MAIPS4510, Millipore). These plates had been previously activated with 35% ethanol, washed with PBS, and coated with goat anti-mouse IgM, goat anti-mouse IgG, goat anti-mouse IgA, NP_4_-BSA, or *S.* Typhimurium flagellin (all 5 μg/ml). A total of 1.25 × 10^5^ and 2.5 × 10^5^ spleen mononuclear cells and bone marrow cells were plated and cultured at 37°C overnight to analyze the total and specific ASCs, respectively. A total of 1 × 10^4^ and 2 × 10^4^ cultured B cells were plated and cultured under the same conditions to analyze the total and specific ASCs, respectively. After supernatants were removed, plates were incubated with biotinylated goat anti-mouse IgM, IgG3, IgG1, IgG2b, IgG2a, or IgA mAbs, as indicated, for 2 hours and, after washing, incubated with HRP-conjugated streptavidin followed by VECTASTAIN AEC (3-amino-9-ethylcarbazole) peroxidase substrate (SK-4200, Vector Laboratories). Individual ASC spots were enumerated using the CTL ImmunoSpot software (Cellular Technology). ASCs emerging from cultured IgM^+^IgD^+^ B cells that underwent CSR and plasma cell differentiation were identified and enumerated as above.

### Quantitative reverse transcription polymerase chain reaction

To quantify *Aicda*, *Prdm1*, *Tlr4*, *Tlr5*, *AICDA*, *PRDM1*, *TLR4*, *TLR5*, germline I_H-_C_H_, and post-recombination Iμ-C_H_ transcripts, RNA was extracted from 5 × 10^6^ mouse or human B cells using the Direct-zol RNA MicroPrep Kit (R2060, Zymo Research). First-strand complementary DNA (cDNA) was synthesized from equal amounts of total RNA (4 μg) with the SuperScript III System (18080051, Invitrogen) using oligo(dT) primer. Transcript expression was analyzed using SYBR Green dye (115010139, IQ SYBR Green Supermix, Bio-Rad) incorporation in PCR reactions involving specific primers (tables S10 and S11). Reactions were performed using a MyIQ Single Color Real-Time qPCR Detection System (Bio-Rad) under the following amplification cycles: 95°C for 15 s, 40 cycles at 94°C for 10 s, 60°C for 30 s, and 72°C for 30 s; data acquisition was performed during this 72°C extension step. Melting curve analysis was performed from 72° to 95°C. The 2^−ΔCt^ method {2^−ΔCt^ = 2^−[Ct(β*-ACTIN*)–Ct(target gene)]^} was used to determine levels of transcripts, and data were normalized to levels of *Gapdh* or β*-ACTIN*.

### B cell clonality, SHM, and intraconal diversification

To analyze Ig SHM, spleen B cells were isolated from C57BL/6 or *Tcr*β^−/−^*Tcr*δ^−/−^ mice that were injected with NP-LPS, NP-CGG, or *S.* Typhimurium flagellin and then subjected to RNA extraction using the Direct-zol RNA MicroPrep Kit (R2060, Zymo Research). cDNA was synthesized from 1–2 μg total RNA with the SuperScript III First-Strand Synthesis System (18080051, Invitrogen) using oligo(dT) primer. Rearranged V1-72DJ_H_-Cγ3, V1-72DJ_H_-Cγ1 and V1-72DJ_H_-Cγ2b cDNA encoding the anti-NP VDJ-IgH chain were amplified using a V1-72 leader-specific forward primer together with nested reverse Cγ3-, Cγ1-, and Cγ2b-specific primers (table S10) tagged with Illumina clustering adapters and Phusion high-fidelity DNA polymerase (M0530S, New England BioLabs). Rearranged V_H_1DJ_H_-Cμ, V_H_1DJ_H_-Cγ3, V_H_1DJ_H_-Cγ2b, and V_H_1DJ_H_-Cα cDNA from flagellin-injected *Tcr*β^−/−^*Tcr*δ^−/−^ mice were amplified using a mixture of leader primers specific for different C57BL/6 V_H_1 family genes together with nested reverse Cμ-, Cγ3-, Cγ2b-, and Cα-specific primers (table S10) tagged with Illumina clustering adapters and Phusion high-fidelity DNA polymerase. PCR amplification conditions were 98°C for 10 s, 60°C for 45 s, and 72°C for 1 min for 30 cycles. The amplified library was tagged with barcodes for sample multiplexing, PCR enriched, and annealed to the required Illumina clustering adapters. High-throughput 300–base pair pair-ended sequencing was performed using the Illumina MiSeq system. Somatic point mutations in V1-72 (V186.2) and V_H_1 segments were analyzed using *IMGT/*HighV-*QUEST* (the IMGT information system; www.imgt.org).

To identify and analyze B cell clones making antibodies to NP and flagellin, B cell V1-72DJ_H_-Cγ3, V1-72DJ_H_-Cγ1, and V1-72DJ_H_-Cγ2b transcripts (approximately 100,000 sequences each per mouse) as well as V_H_1DJ_H_-Cγ3, V_H_1DJ_H_-Cγ2b, and V_H_1DJ_H_-Cα transcripts (approximately 10,000 sequences each per mouse), respectively, were segregated on the basis of the V1-72 (in NP-LPS–injected mice) or the V_H_1 gene segment (in flagellin-injected mice), the same and unique IgH CDR3 (depicted as translated amino acid sequence) together with the same J_H_ sequence by MiSeq amplicon sequencing. Each discrete clone was depicted as an individual rectangle or square of a unique color, whose area reflected the B cell clone size (TreeMap, Microsoft Excel). The shared and unique point mutations in V1-72DJ_H_-Cγ3, V1-72DJ_H_-Cγ1, V1-72DJ_H_-Cγ2b, V_H_1DJ_H_-Cγ3, V_H_1DJ_H_-Cγ2b, and V_H_1DJ_H_-Cα transcripts within each clone from *Tcr*β*^−/−^Tcr*δ*^−/−^* and C57BL/6 mice allowed for construction of genealogical trees (phylogenic maps), each with multiple branches emanating from a common progenitor and revealing complex intraconal diversification. Clones from NP-LPS–injected, NP-CGG–injected, and flagellin-injected mice were classified as dominant clones (those comprising a mean of 963 to 1869 V1-72DJ_H_-Cγ3 or V1-72DJ_H_-Cγ2b transcripts in NP-LPS–injected *Tcr*β*^−/−^Tcr*δ*^−/−^* and C57BL/6 mice, 847 to 3090 V1-72DJ_H_-Cγ1 transcripts in NP-CGG–injected C57BL/6 mice, or 116 to 1239 V_H_1DJ_H_-Cγ3, V_H_1DJ_H_-Cγ2b, or V_H_1DJ_H_-Cα transcripts in flagellin-injected *Tcr*β*^−/−^Tcr*δ*^−/−^* mice, respectively, all identical but differing only by unique mutations), intermediate clones (mean of 201 to 826, 202 to 336, or 28 to 117 transcripts, respectively), small clones (mean of 127 to 194, 23 to 30, or 9 to 17 transcripts, respectively), and microclones (mean of 2 to 5, 1 to 2, or 1 to 2 transcripts, respectively). Genealogical trees and scatter tree plots were constructed by uploading FASTA files of all segregated V1-72DJ_H_-Cγ3, V1-72DJ_H_-Cγ1, V1-72DJ_H_-Cγ2b, V_H_1DJ_H_-Cγ3, V_H_1DJ_H_-Cγ2b, and V_H_1DJ_H_-Cα transcripts onto PHYLOViZ Online (www.phyloviz.net), which uses a goeBURST algorithm for visualization of multiple phylogenetic inference trees. Scatter tree plots shown (fig. S11B) depict a partial window of the multitude of clonal genealogical trees for V1-72DJ_H_-Cγ3 and V1-72DJ_H_-Cγ2b transcripts in each C57BL/6 and *Tcr*β*^−/−^Tcr*δ*^−/−^* mouse.

### NF-κB activation

*Tcr*β*^−/−^Tcr*δ*^−/−^* spleen B cells (10^7^) were resuspended in FCS-RPMI and stimulated with mCD154 (3 U/ml) plus IL-4 (4 ng/ml), NP-LPS (3 μg/ml), or *S.* Typhimurium flagellin (10 μg/ml) for indicated times. Cell lysates were separated by SDS–polyacrylamide gel electrophoresis and transferred onto nitrocellulose membranes. Membranes were subjected to sequential immunoblotting with anti–phospho-IκBα or anti–phospho-p65 mAb (Cell Signaling Technology; canonical NF-κB pathway) or anti-p52 pAb, also detecting p100 (Cell Signaling Technology; noncanonical NF-κB pathway). For analysis of the canonical NF-κB pathway, membranes were stripped using Restore PLUS Western blot Stripping Buffer (46430, Thermo Fisher Scientific) at 25°C for 15 min and subjected to immunoblotting involving anti-IκBα or anti-p65 mAb and stripped again for immunoblotting involving anti–β-actin mAb (Sigma-Aldrich). For analysis of the noncanonical NF-κB pathway, membranes were stripped and subjected to immunoblotting involving anti–β-actin mAb.

### Infection of vaccinated C57BL/6 and *Tcr*β*^−/−^Tcr*δ*^−/−^* mice with *E. coli* and *S.* Typhimurium

In all experiments, *Tcr*β*^−/−^Tcr*δ*^−/−^* mice were vaccinated by intraperitoneal injection with LPS (25 μg in 100 μl of PBS) on days 0, 7, and 21 or purified flagellin on days 0 (50 μg in 100 μl of alum), 7, and 21 (50 μg in 100 μl of PBS). Vaccinated mice and their nonvaccinated counterparts were challenged with live bacteria 3 days after the last vaccination. *E. coli* O55:B5 was purchased from the American Type Culture Collection (ATCC 12014). *S.* Typhimurium IR715, a fully virulent nalidixic acid–resistant derivative of WT isolate ATCC 14028, was provided by M. Raffatellu (University of California, San Diego). *E. coli* and *S.* Typhimurium were grown in Luria-Bertoni (LB) broth for 14 to 16 hours overnight at 37°C. Log-phase cultures were prepared by diluting overnight cultures to an optical density at 600 nm (OD_600_) of 0.05 in fresh LB medium and incubating them at 37°C, with shaking at 250 rpm until an OD_600_ of 0.7 or 0.8 was attained. Infectious stock cultures were prepared by diluting 500 μl of log-phase cultures with 500 μl of 50% glycerol (sterile filtered) after estimating bacterial concentration using a spectrophotometer. *Tcr*β*^−/−^Tcr*δ*^−/−^* mice were infected intravenously with *E. coli* (1.2 × 10^8^ CFUs) by lateral tail-vein injection or orally with *S.* Typhimurium (10^5^ CFUs) by gavage. The effective dose of bacteria given to the mice was verified by plating dilutions of *E. coli* or *S.* Typhimurium on LB agar or LB agar plates supplemented with nalidixic acid, respectively.

### Mouse bacterial load in the blood, spleen, liver, and feces; weight loss; and survival

Bacterial burden was measured as follows: Blood, tissues, and feces were harvested and homogenized at 1 g/ml in PBS. Homogenates were serially diluted from 1:100 to 1:10,000,000 in PBS and then struck (100 μl) on LB agar plates or LB agar plates supplemented with nalidixic acid. After 14 to 16 hours, colonies were counted. Body weight loss and survival were monitored daily, and weight loss (expressed as percentage of mouse body weight immediately before infection) and Kaplan-Meier survival graphs were generated (Prism GraphPad Software). Body weight reduction of 20% or more is defined as “severe suffering” (as established by guidelines set forth in the *Guide for the Care and Use of Laboratory Animals* from the U.S. National Research Council Institute for Laboratory Animal Resources) and was set as the humane end point (*98*).

### Antibodies to *E. coli* and *S.* Typhimurium

IgG antibodies to *E. coli* 055:B5 LPS (Sigma-Aldrich) were titrated and analyzed by specific enzyme-linked immunosorbent assay (ELISA). Sera from LPS-vaccinated and PBS-injected *Tcr*β*^−/−^Tcr*δ*^−/−^* mice were diluted 50-fold (i.e., 1:50) in PBS–Tween 20 (v/v) and pretreated with 0.05 M β-mercaptoethanol to avoid masking by IgM. Serum samples were then twofold serially diluted (1:50 to 1:102,400) and then incubated in a 96-well plate coated with LPS (1 μg/ml) for 1.5 hours. LPS-bound IgG antibodies were detected with biotinylated goat anti-mouse IgG (SouthernBiotech), followed by HRP-labeled streptavidin and OPD substrate. LPS-specific IgG binding curves, expressed as RUs, were generated using GraphPad Prism software (antibody titers depicted as nonlinear regression curve fit). For neutralization assays, *E. coli* stock cultures were diluted in PBS to a concentration of 10^4^ CFUs/ml. Serum antibody neutralization power was measured by antibody-mediated killing of *E. coli* in the presence of baby rabbit complement. Pooled sera from LPS-vaccinated or PBS-injected *Tcr*β*^−/−^Tcr*δ*^−/−^* mice were twofold serially diluted (1:1 to 1:128) in PBS in round-bottom 96-well plates. Serially diluted sera (50 μl) were mixed with 25 μl of baby rabbit complement (25% final concentration; CL3441, CEDARLANE) and incubated with 25 μl of diluted *E. coli* (150 CFUs). Each mixture sample was shaken (115 rpm) at 37°C for 1 hour and then struck onto LB agar plates. These were incubated at 37°C overnight, after which viable CFUs were enumerated. Negative controls consisted of PBS, baby rabbit complement, and bacteria. Serum neutralizing (bactericidal) activity was measured by the reduction of *E. coli* CFUs.

IgG antibodies to *S.* Typhimurium flagellin were titrated and analyzed by specific ELISA. Sera from *Tcr*β*^−/−^Tcr*δ*^−/−^* mice vaccinated with flagellin (in alum) or control *Tcr*β*^−/−^Tcr*δ*^−/−^* mice (alum alone) were first diluted 100-fold in PBS–Tween 20 and twofold serially diluted (1:100 to 1:12800) in PBS–Tween 20 (v/v) and then incubated in a 96-well plate coated with purified flagellin (1 μg/ml) for 1.5 hours. Flagellin-bound IgG antibodies were detected with biotinylated goat anti-mouse IgG (SouthernBiotech), followed by HRP-labeled streptavidin and OPD substrate. Flagellin-specific IgG binding curves, expressed as RUs, were generated using GraphPad Prism software (antibody titers depicted as nonlinear regression curve fit). *S.* Typhimurium serum antibody neutralization power was measured by antibody-mediated killing of *S.* Typhimurium in the presence of baby rabbit complement. *S.* Typhimurium stock cultures were diluted in PBS to a concentration of 10^4^ CFUs/ml. Pooled sera from *Tcr*β*^−/−^Tcr*δ*^−/−^* mice vaccinated with flagellin in alum, C57BL/6 mice injected with alum only, and C57BL/6 mice vaccinated with flagellin in alum were diluted twofold in PBS in round-bottom 96-well plates. Serially diluted sera (50 μl) were mixed with 25 μl of baby rabbit complement (25% final concentration) and incubated with 25 μl of diluted *S.* Typhimurium (250 to 2000 CFUs). Each mixture sample was shaken (115 rpm) at 37°C for 1 hour and then struck onto LB agar plates. These were incubated at 37°C overnight, after which CFUs were enumerated. Negative controls consisted of PBS, baby rabbit complement, or bacteria only in the absence of mouse serum. Serum neutralizing (bactericidal) activity was measured by reduction of *S.* Typhimurium CFUs in samples incubated with sera as compared to seeded CFUs (negative control). The high affinity of anti-flagellin antibodies was determined by the ability of free flagellin to inhibit these antibodies from neutralizing *S.* Typhimurium in the fluid phase. Pooled sera (50 μl) from flagellin-vaccinated *Tcr*β*^−/−^Tcr*δ*^−/−^* mice were mixed with free purified flagellin (10 μg) in PBS or PBS alone and incubated at room temperature for 2 hours. The sera incubated with or without flagellin were then transferred to round-bottom 96-well plates and mixed with baby rabbit complement (25 μl) and 200 CFUs (25 μl) of *S.* Typhimurium. The resulting mixtures were struck onto LB agar plates, which were then incubated at 37°C overnight, after which viable CFUs were enumerated.

### Statistical analyses

Statistical analyses were performed by GraphPad (Prism) or Excel (Microsoft) software. *P* values were determined by unpaired Student’s *t* test. Differences in mouse survival were calculated using the log-rank (Mantel-Cox) test.

## Notes

View/request a protocol for this paper from *Bio-protocol*.
