# Supplementary material for: Intrinsic B cell TLR-BCR linked coengagement induces class-switched, hypermutated, neutralizing antibody responses in absence of T cells
Source: Sci Adv. 2023 Apr 28;9(17):eade8928. doi: 10.1126/sciadv.ade8928 (PMC10146914; doi:10.1126/sciadv.ade8928)
Supplement: Supplementary file 1 — Figs. S1 to S18 Table S1 to S11 [file sciadv.ade8928_sm.pdf]

Science Advances
AAAS

Supplementary Materials for

Intrinsic B cell TLR-BCR linked coengagement induces class-switched, hypermutated, neutralizing antibody responses in absence of T cells

Carlos E. Rivera *et al.*

Corresponding author: Paolo Casali, pcasali@uthscsa.edu

Sci. Adv. **9**, eade8928 (2023)
DOI: 10.1126/sciadv.ade8928

This PDF file includes:

Figs. S1 to S18
Table S1 to S11

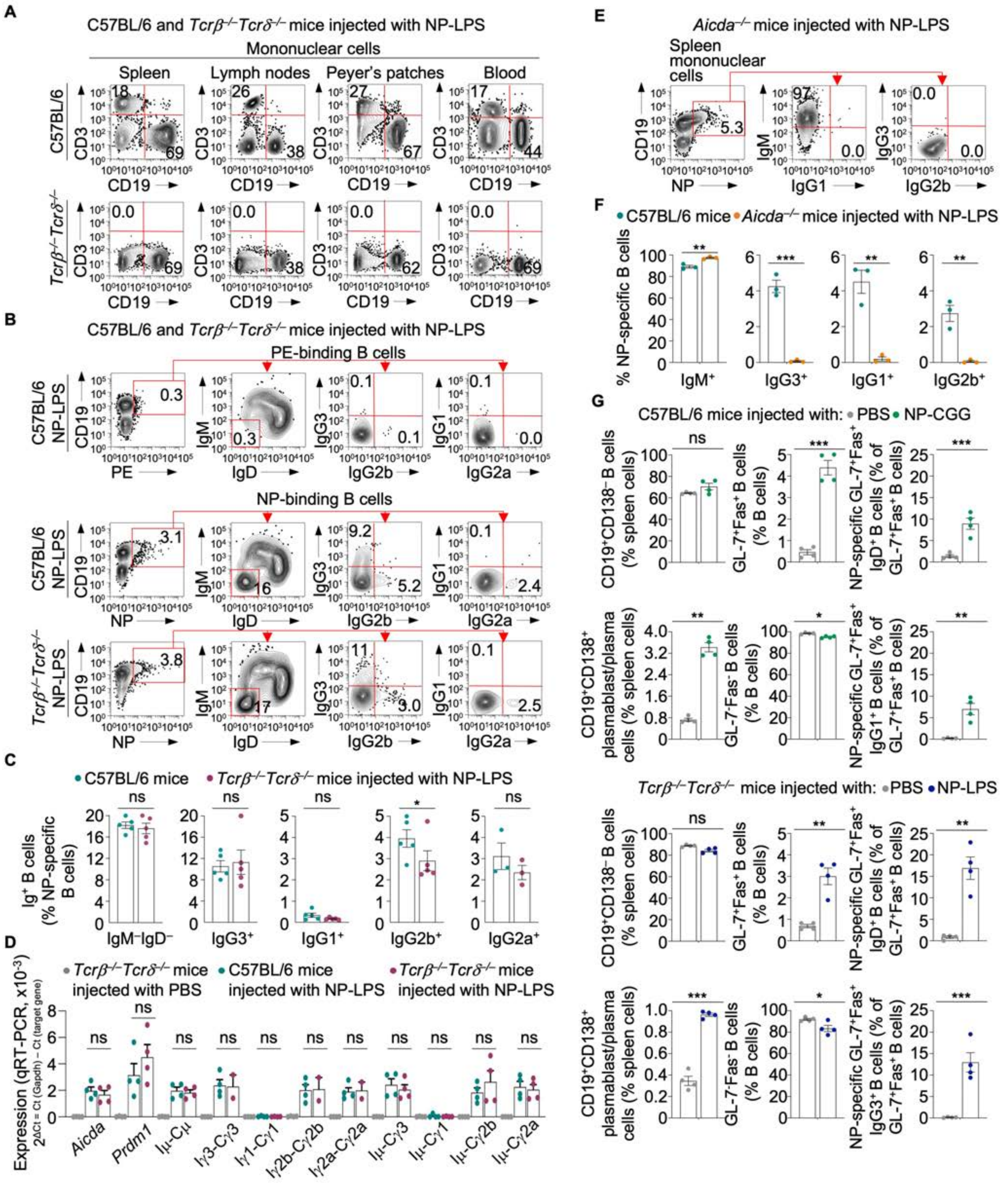

Fig. S1. TLR4-BCR coengagement by NP-LPS induces a NP-specific and class-switched antibody response in *Tcrβ*$^{-/-}$*Tcrδ*$^{-/-}$ mice and C57BL/6 control mice. (**A**) Lack of T cells in *Tcrβ*$^{-/-}$*Tcrδ*$^{-/-}$ mice. C57BL/6 and *Tcrβ*$^{-/-}$*Tcrδ*$^{-/-}$ mice were injected i.p. with NP-LPS (100 μg in 100 μl PBS) and euthanized 14 days after injection. Mononuclear cells from spleens, lymph nodes, Peyer's patches and peripheral blood were analyzed by FACS for CD3^{+} T cells and CD19^{+} B cells, as identified by surface staining with anti-CD3

and anti-CD19 mAbs. Numbers are percentages of total mononuclear cells (data are representative of three C57BL/6 and three *Tcrβ*$^{-/-}$*Tcrδ*$^{-/-}$ mice). (**B**) *Tcrβ*$^{-/-}$*Tcrδ*$^{-/-}$ mice make NP-specific class-switched B cells in response to T-independent NP-LPS. C57BL/6 and *Tcrβ*$^{-/-}$*Tcrδ*$^{-/-}$ mice were injected i.p. with NP-LPS (100 μg in 100 μl PBS) on days 0 and 21 (n = 5 mice per group). Mice were euthanized 14 days after the second injection (day 35). Spleen mononuclear cells were analyzed by FACS for CD19$^+$ NP-specific B cells, as identified by surface staining with anti-CD19 mAb and binding of NP-PE or PE (negative control). NP-specific B cells were characterized for surface expression of IgM, IgD, IgG3, IgG2b, IgG1 and IgG2a by specific mAbs (numbers are percentages of mononuclear cells analyzed). Data are one representative of 5 mice. (**C**) Histograms depict mean values of NP-specific IgM$^-$IgD$^-$, IgG3$^+$, IgG1$^+$, IgG2b$^+$ and IgG2a$^+$ B cells ± SEM (% NP-specific B cells) in C57BL/6 and *Tcrβ*$^{-/-}$*Tcrδ*$^{-/-}$ mice (n = 5 mice per group, same mice as in B). Each dot represents an individual mouse. (**D**) *Aicda, Prdm1,* germline I_H-C_H and post-recombination Iμ-Cx (Cx being any switched isotype) transcripts in B cells from *Tcrβ*$^{-/-}$*Tcrδ*$^{-/-}$ mice injected with PBS and C57BL/6 and *Tcrβ*$^{-/-}$*Tcrδ*$^{-/-}$ mice injected with NP-LPS (n = 4 mice per group), as measured by qRT-PCR and normalized to *Gapdh* expression ($2^{-\Delta Ct}$ method). (**E, F**) *Aicda*$^{-/-}$ and C57BL/6 mice were injected i.p. with NP-LPS (100 μg in 100 μl PBS) and euthanized 14 days later. Spleen mononuclear cells were analyzed by FACS for CD19$^+$ NP-specific B cells as in (B). NP-specific B cells were characterized for surface expression of IgM, IgG3, IgG1 and IgG2b by specific mAbs (n = 3 mice per group, data are from one experiment representative of three mice) (E). Histograms depict mean values of NP-specific IgM$^+$, IgG3$^+$, IgG1$^+$ and IgG2b$^+$ B cells ± SEM (% NP-specific B cells) in NP-LPS-immunized *Aicda*$^{-/-}$ and C57BL/6 mice. Each dot represents an individual mouse (F). (**G**) Histograms depict mean values of B cells, NP-specific IgD$^+$, IgG3$^+$ and IgG1$^+$ GL7$^+$Fas$^+$ GC-like B cells and CD19$^+$CD138$^+$ plasmablasts/plasma cells ± SEM in PBS-injected and NP-CGG-immunized C57BL/6 mice and PBS-injected and NP-LPS-immunized *Tcrβ*$^{-/-}$*Tcrδ*$^{-/-}$ mice. Each dot represents an individual mouse (n = 4 mice per group, same mice as in Fig. 1C). *p <0.05, **p <0.01, ***p <0.001, not significant (ns) (unpaired *t*-test).

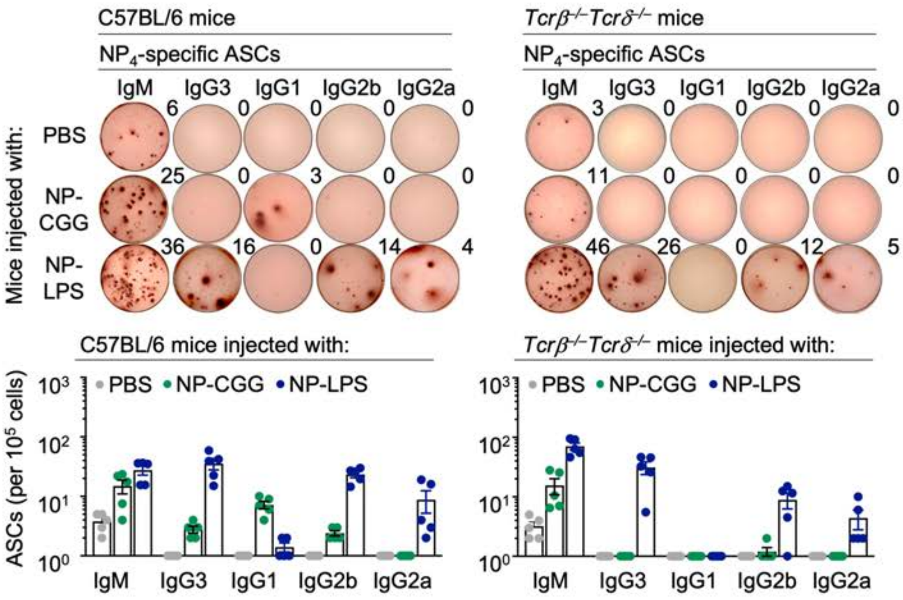

Fig. S2. TLR4-BCR coengagement by NP-LPS elicits NP-specific and class-switched ASCs in *Tcrβ*$^{-/-}$ *Tcrδ*$^{-/-}$ mice at levels comparable to C57BL/6 mice. C57BL/6 and *Tcrβ*$^{-/-}$*Tcrδ*$^{-/-}$ mice were injected i.p. with PBS (100 µl), NP-CGG (100 µg in 100 µl PBS) or NP-LPS (100 µg in 100 µl PBS) on days 0 and 21 (n = 5 mice per group, same mice as in Fig. S1C). Mice were euthanized 14 days after the second injection (day 35) and spleen cells were analyzed for NP$_4$-specific IgM, IgG3, IgG1, IgG2b and IgG2a ASCs by ELISPOT. Data depicted are from one representative of 5 C57BL/6 and 5 *Tcrβ*$^{-/-}$*Tcrδ*$^{-/-}$ mice. Histograms depict mean numbers of ASCs per 10^5 plated cells $\pm$ SEM. Each dot represents an individual mouse. The large IgG3, IgG2b and IgG2a spots among plated cells from mice immunized with NP-LPS reflected NP-specific antibody-producing plasmablasts/plasma cells induced by LPS. The large IgG1 spots among plated cells from C57BL/6 mice immunized with NP-CGG reflected plasmablasts and plasma cells induced by this T-dependent conjugated hapten.

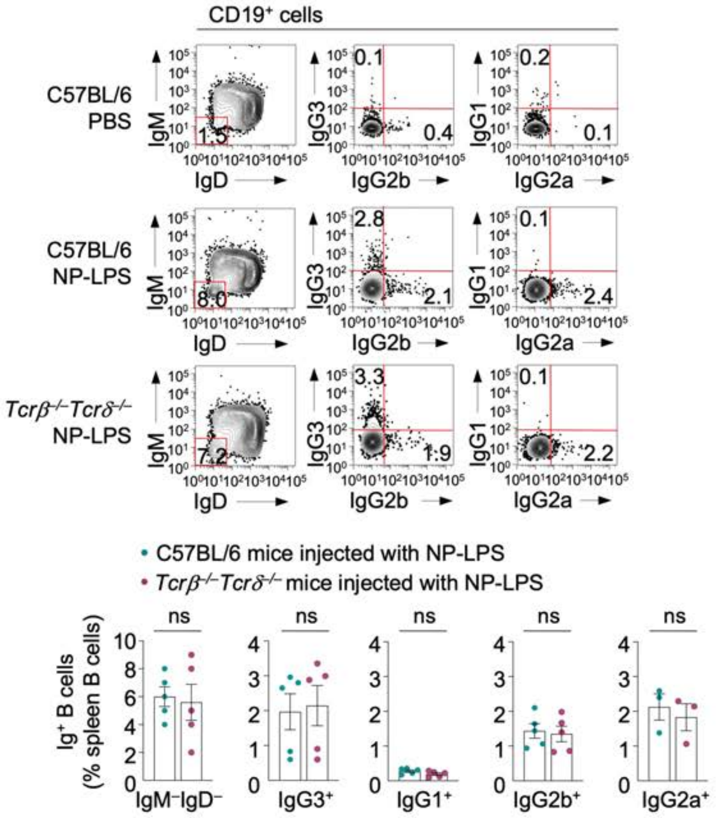

Fig. S3. TLR4-BCR coengagement elicits class-switched B cells in *Tcrβ*$^{-/-}$*Tcrδ*$^{-/-}$ mice at levels comparable to C57BL/6 mouse controls. C57BL/6 and *Tcrβ*$^{-/-}$*Tcrδ*$^{-/-}$ mice were injected i.p. with PBS (100 μl) or NP-LPS (100 μg in 100 μl PBS) on days 0 and 21 (n = 5 mice per group, same mice as in Fig. S1C). Mice were euthanized 14 days after the second injection (day 35). Spleen mononuclear cells were analyzed by FACS for B cells, as identified by surface staining with anti-CD19 mAb. Such B cells were characterized for surface expression of IgM, IgD, IgG3, IgG2b and IgG2a by specific mAbs (numbers are percentages of total B cells). Histograms depict mean values of IgM^{-}IgD^{-}, IgG3^{+}, IgG1^{+}, IgG2b^{+} and IgG2a^{+} B cells ± SEM (% spleen B cells) in C57BL/6 and *Tcrβ*$^{-/-}$*Tcrδ*$^{-/-}$ mice. Each dot represents an individual mouse. Not significant (ns) (unpaired *t*-test).

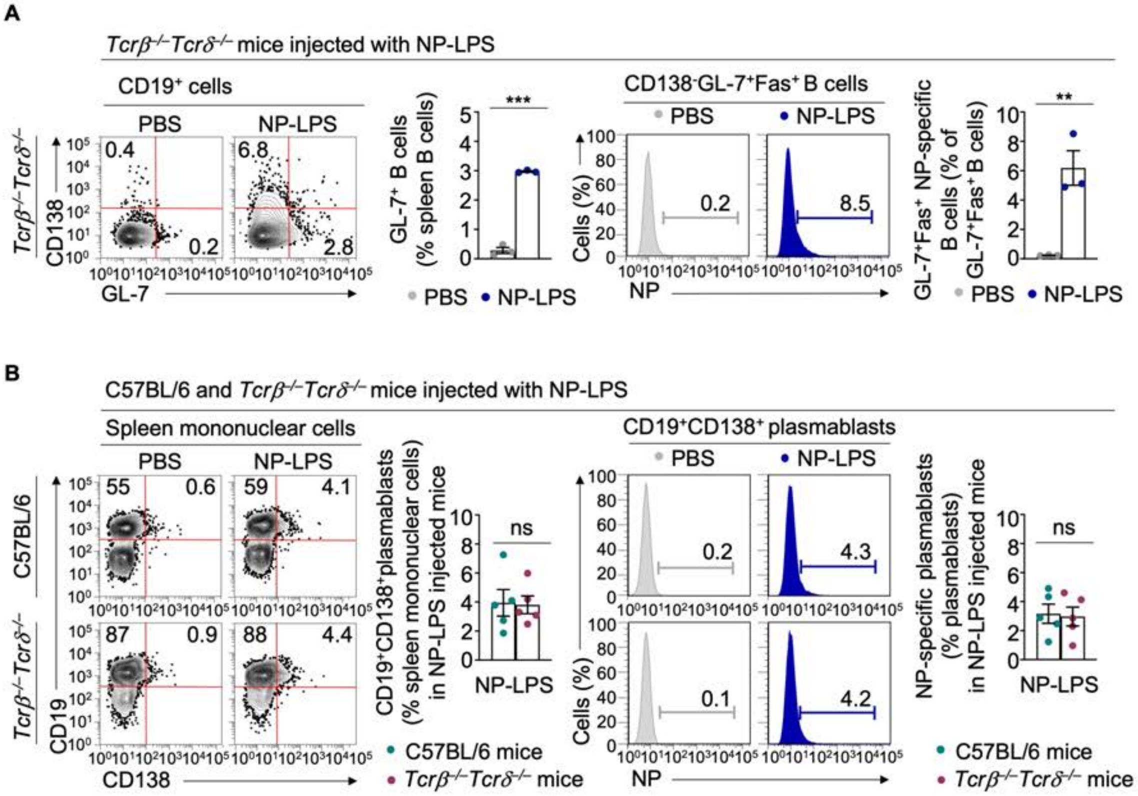

Fig. S4. TLR4-BCR linked coengagement by NP-LPS induces NP-specific GC-like B cells and plasmablasts in *Tcrβ*$^{-/-}$*Tcrδ*$^{-/-}$ mice. (**A**) *Tcrβ*$^{-/-}$*Tcrδ*$^{-/-}$ mice were injected i.p. with PBS (100 µl) or NP-LPS (100 µg in 100 µl PBS) (n = 3 mice per group) and euthanized 10 days after injection. Spleen mononuclear cells were analyzed by FACS for B cells, as identified by surface staining with anti-CD19 mAb. Such B cells were characterized for surface expression of CD138, GL-7 and CD95 (Fas) by anti-CD138, anti-GL-7 and anti-CD95 mAbs (numbers are percentages of total B cells). NP-specific CD138^{-}GL-7^{+}Fas^{+} B cells were then identified by the binding of NP-PE. Histograms depict mean values of GL-7^{+} (% of total spleen B cells) and GL-7^{+}Fas^{+} NP-specific B cells $\pm$ SEM (% of GL-7^{+}Fas^{+} B cells). Each dot represents an individual mouse. (**B**) C57BL/6 and *Tcrβ*$^{-/-}$*Tcrδ*$^{-/-}$ mice were injected with PBS (100 µl) or NP-LPS (100 µg in 100 µl PBS) on days 0 and 21 (n = 5 mice per group, same mice as in Fig. S1C). Mice were euthanized 14 days after the second injection (day 35). Spleen mononuclear cells were analyzed by FACS for total and NP-specific plasmablasts, as identified by surface staining with anti-CD19 and anti-CD138 mAbs and the binding of NP-PE. Histograms depict mean values of CD19^{+}CD138^{+} cells (% spleen mononuclear cells) and NP-specific CD19^{+}CD138^{+} cells $\pm$ SEM (% plasmablasts). Each dot represents an individual mouse. ***p* <0.01, ****p* <0.001, not significant (ns) (unpaired *t*-test).

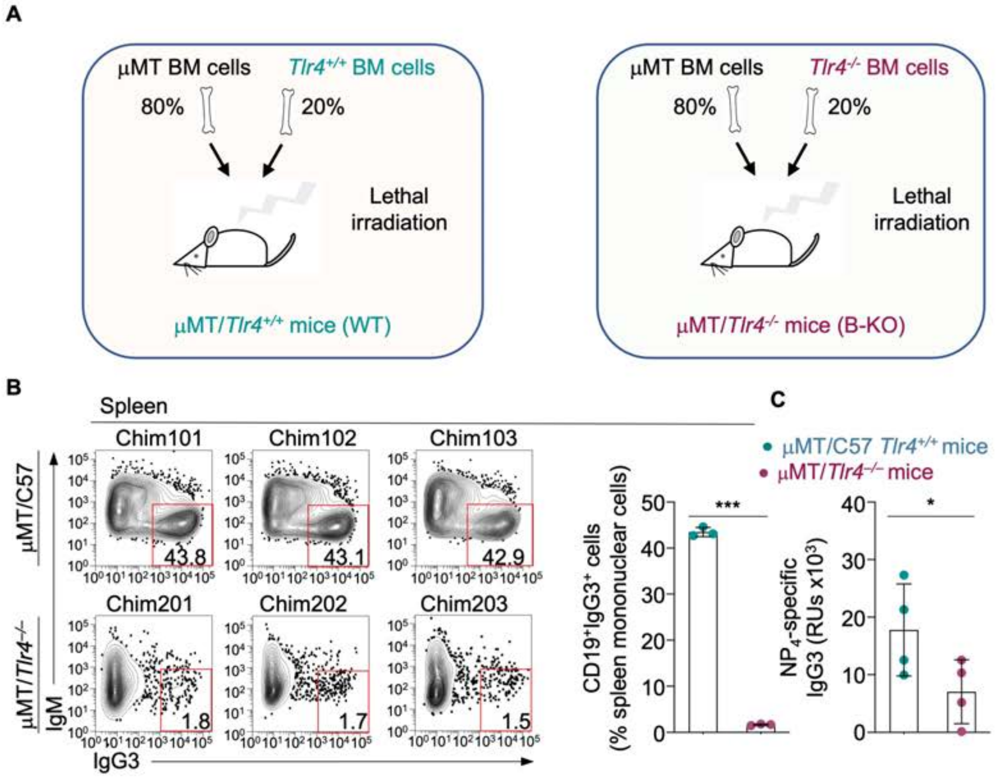

Fig. S5. B cell TLR4 supports T-independent CSR leading to NP-specific class-switched antibodies. (**A**) Bone marrow chimera μMT/*Tlr4*$^{+/+}$ mice and μMT/*Tlr4*$^{–/–}$ mice were constructed by mixing bone marrow cells from μMT mice (4.0 x 10^6) with bone marrow cells from *Tlr4*$^{+/+}$ (C57BL/6) or *Tlr4* $^{–/–}$ mice (1.0 x 10^6) and injecting the cell mixture i.v. into irradiated recipient C57BL/6 mice. (**B and C**) After 6-8 weeks to allow for reconstitution of the immune system, chimeric μMT/*Tlr4*$^{+/+}$ and μMT/*Tlr4*$^{–/–}$ mice were injected i.p. with NP-LPS (100 μg in 100 μl PBS) and bled 21 days later (n = 3 mice per group in B and n = 4 mice per group in C). (**B**) Spleen mononuclear cells were analyzed by FACS for B cells, as identified by surface staining with anti-CD19 mAb. Such B cells were characterized for surface expression of IgM and IgG3 by specific mAbs (numbers are percentages of spleen mononuclear cells). Histogram depicts mean values of IgG3$^+$ B cells $\pm$ SEM (% spleen B cells). Each dot represents an individual mouse. The residual IgG3$^+$ B cells in NP-LPS-injected μMT/*Tlr4*$^{–/–}$ mice were possibly made by "leaked" B cells from μMT donor bone marrow. (**C**) Circulating NP$_4$-specific IgG3, as analyzed by ELISA (antibody titers expressed as RUs). Histogram depicts mean titers $\pm$ SEM. Each dot represents an individual mouse. *p <0.05, ***p <0.001 (unpaired t-test).

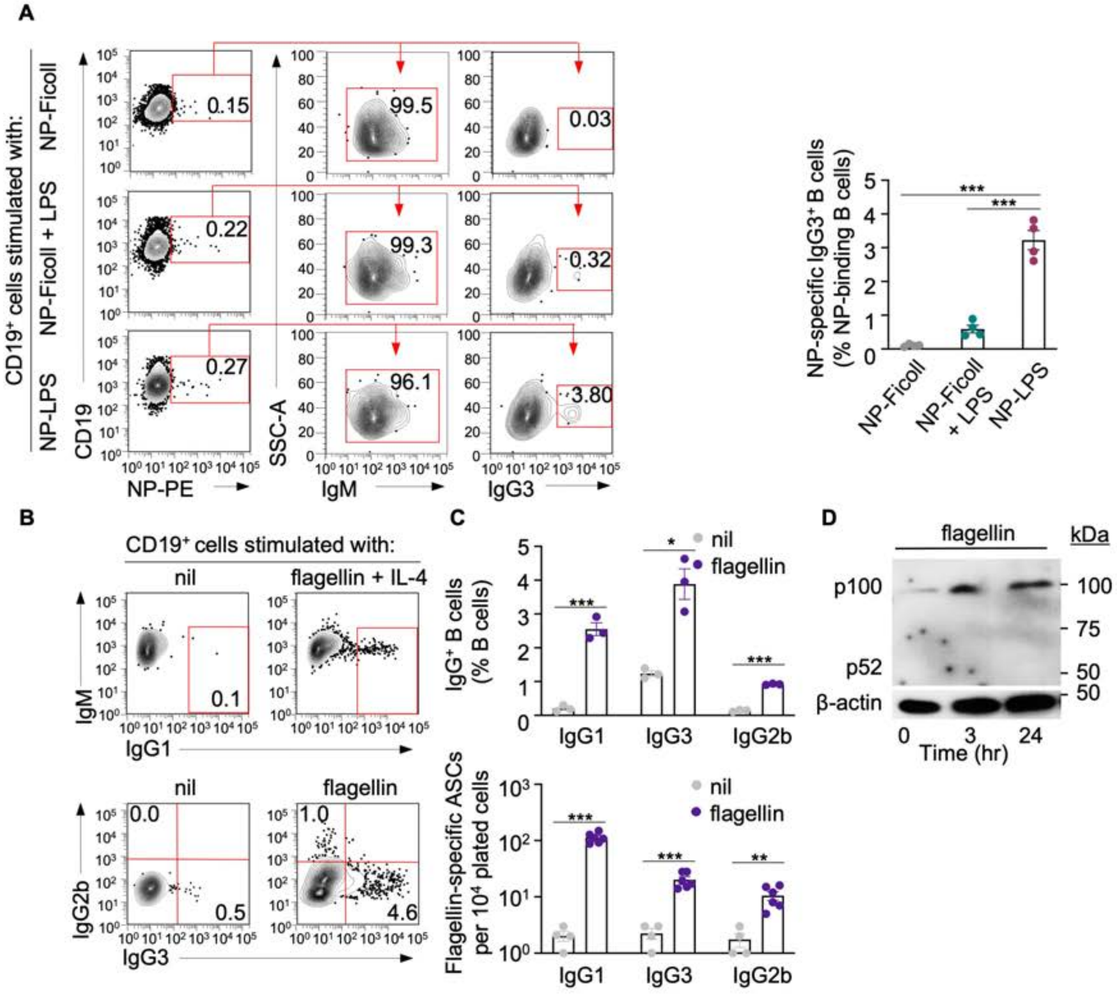

Fig. S6. TLR4-BCR and TLR5-BCR linked coengagement induces specific class-switched B cells *in vitro*. (**A**) Naïve IgM$^+$IgD$^+$ B cells from C57BL/6 mice spleens were stimulated with NP-Ficoll (3.0 µg/ml), NP-Ficoll plus LPS (3.0 µg/ml each) or NP-LPS (3.0 µg/ml) *in vitro*. After 96 hours, NP-specific B cells were identified by anti-CD19 mAb and binding of NP-PE (B cells stained with PE alone as negative controls were less than 0.1%, not shown) and characterized for surface IgM and IgG3 by specific mAbs, as analyzed by FACS (numbers are percentages). Histograms depict mean values of NP-specific IgG3$^+$ B cells $\pm$ SEM (% NP-specific B cells). Data points represent cell culture replicates. (**B**) Naïve IgM$^+$IgD$^+$ B cells from C57BL/6 mice spleens were stimulated with nil (unstimulated) or *S.* Typhimurium flagellin (10 µg/ml) in the presence or absence of IL-4 (3 ng/mL) *in vitro*. After 96 hours, IgM$^+$, IgG1$^+$, IgG3$^+$ and IgG2b$^+$ B cells were identified using specific mAbs, as analyzed by FACS (numbers are percentages of total B cells). (**C**) Histograms depict mean values of IgG$^+$ B cells $\pm$ SEM (% B cells). Data points represent cell culture replicates. Cultured B cells (same as in B) were analyzed for flagellin-specific IgG1, IgG3 and IgG2b ASCs by ELISPOT. Histograms depict mean numbers of flagellin-specific IgG1, IgG3 and IgG2b ASCs per 10^4 plated cells $\pm$ SEM. Data points represent six replicates. (**D**) Induction of the non-canonical NF-κB pathway (p100 processed to p52) in $Tcr\beta^{-/-}Tcr\delta^{-/-}$ B cells stimulated *in vitro* with *S.* Typhimurium flagellin (10 µg/ml) for 0-, 3- and 24 hours. Flagellin induced B cell NF-κB p100 and p100 conversion to p52 within 5 to 60 minutes (**Fig. 2D**), followed by exhaustion of such conversion and reversion to p100 expression only by 3 hours. *p <0.05, **p <0.01, ***p <0.001 (unpaired t-test).

A

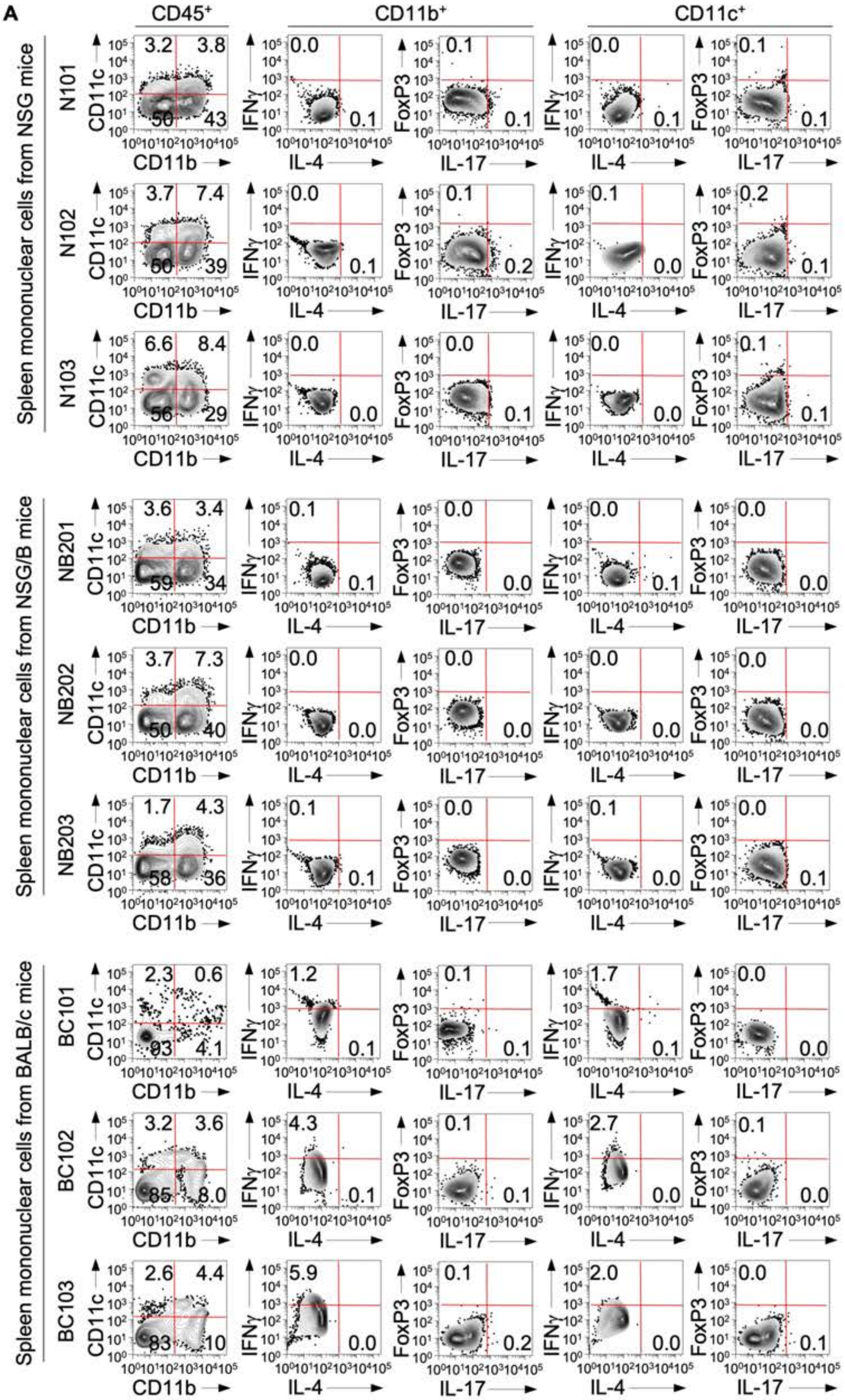
CD45+
CD11b+
CD11c+
Spleen mononuclear cells from NSG mice
N101
N102
N103
Spleen mononuclear cells from NSG/B mice
NB201
NB202
NB203
Spleen mononuclear cells from BALB/c mice
BC101
BC102
BC103
CD11c
CD11b
IFNγ
IL-4
FoxP3
IL-17

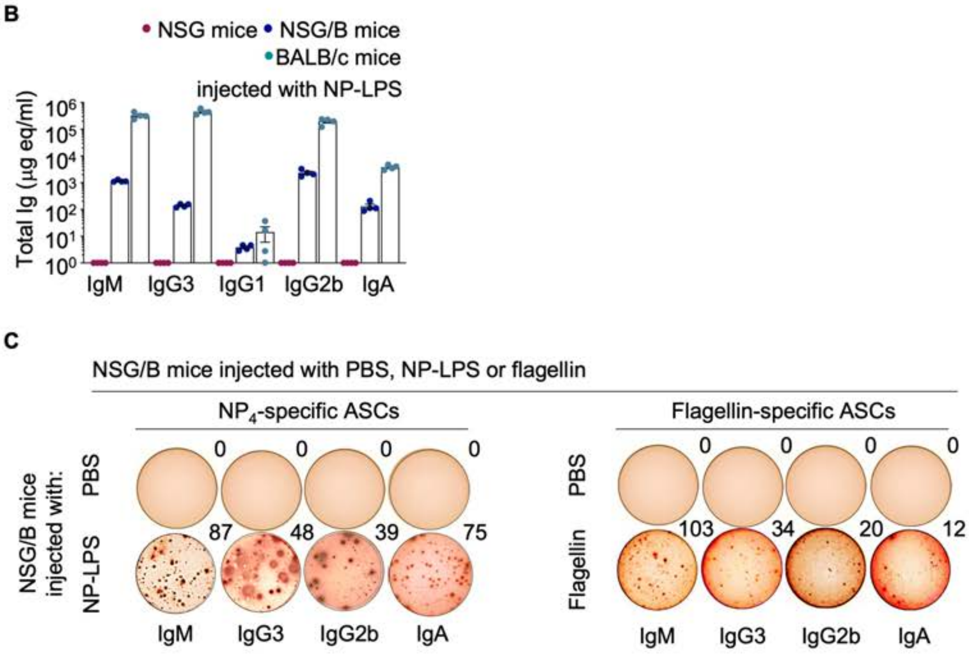

Fig. S7. TLR4-BCR and TLR5-BCR linked coengagement by NP-LPS and flagellin in NSG/B mice induce generation of specific and class-switched ASCs to NP and flagellin. NSG, NSG/B and BALB/c mice were injected i.p. with NP-LPS (100 µg in 100 µl PBS) on days 0, 2 and 4 (n = 3 mice per group, same mice as in Fig. 3B). (**A**) Mice were euthanized 10 days after the third injection (day 14). Spleen mononuclear cells were analyzed by FACS by gating for $CD45^+$ cells, and $CD11b^+$ and $CD11c^+$ cells, as identified by surface staining with specific mAbs. These cells were analyzed for expression of IL-4, IFNγ, IL-17 and FoxP3 by intracellular staining with specific mAbs. Numbers are percentages of total mononuclear cells or total $CD11b^+$ or $CD11c^+$ cells. (**B**) NSG/B mice were injected i.p. with PBS (100 µl) or NP-LPS (100 µg in 100 µl PBS) on days 0, 2 and 4. Similar NSG/B mice were injected with *S.* Typhimurium flagellin (50 µg in 100 µl alum) on day 0, day 2 (50 µg in 100 µl PBS) and day 4 (50 µg in 100 µl PBS) (same mice as in Fig. 3E). The mice were euthanized 10 days after the third injection (day 14) and spleens were analyzed for NP-specific and flagellin-specific IgM, IgG3, IgG2b and IgA ASCs by ELISPOT. Numbers depict ASCs per 10^5 plated cells. Data are from one mouse representative of three PBS-injected, NP-LPS-injected and flagellin-injected NSG/B mice. The large IgG3 and IgG2b spots among plated cells from NP-LPS-immunized mice likely reflected a greater plasma cell differentiation-inducing power of LPS over flagellin. NP$_4$-specific IgA antibodies were elicited in NSG/B mice after three injections with NP-LPS. This contrasted with the non-detectable levels of NP$_4$-specific IgA in $Tcr\beta^{-/-}Tcr\delta^{-/-}$ mice injected only once with NP-LPS, as in other experiments.

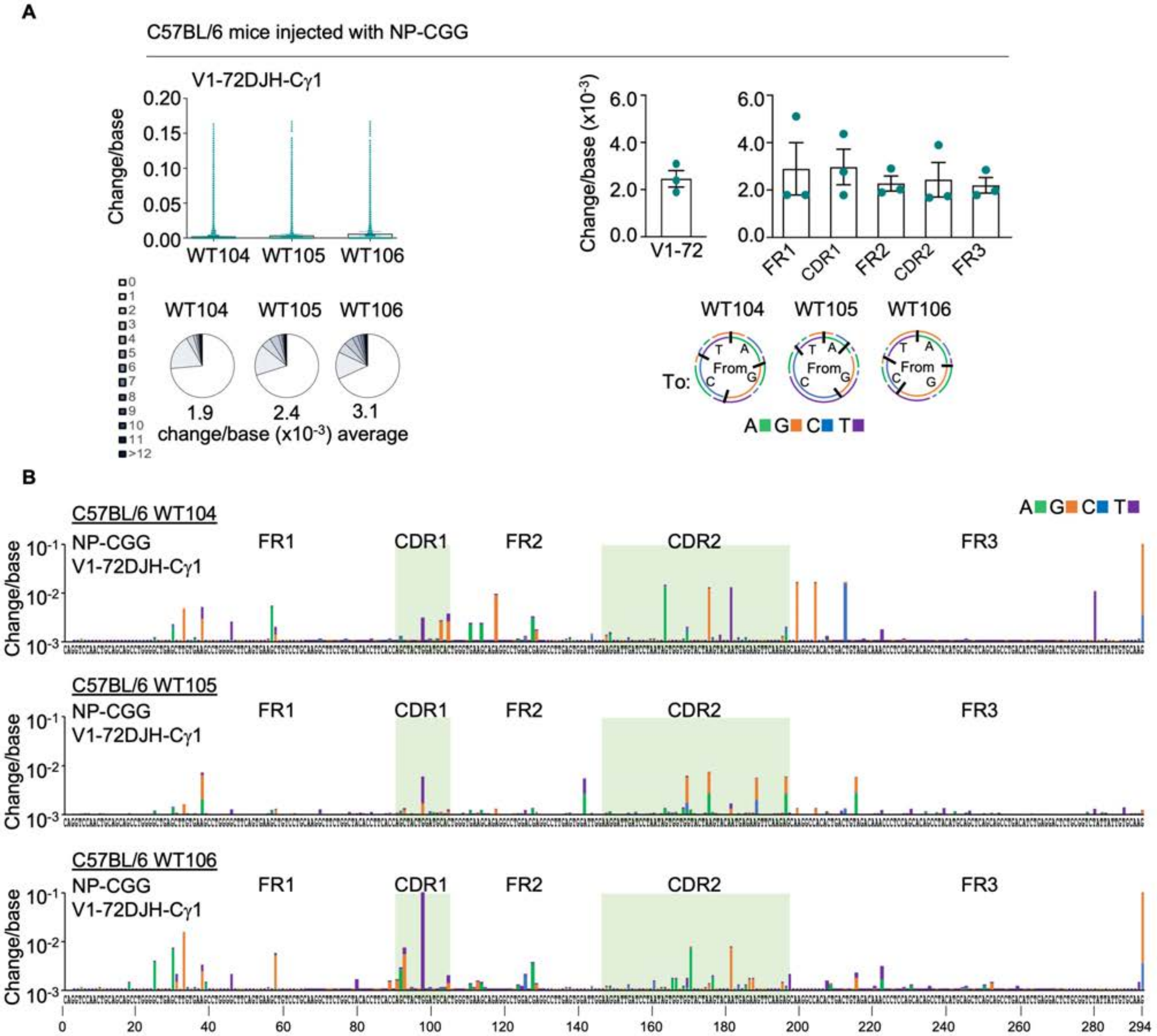

Fig. S8. Somatic point-mutations in the class-switched anti-NP antibody response induced by NP-CGG in C57BL/6 mice. C57BL/6 mice were injected i.p. with NP-CGG (100 µg in 100 µl PBS) on days 0 and 21 (n = 3 mice, WT104, WT105 and WT106). Mice were euthanized 14 days after the second injection (day 35). V1-72DJH-Cγ1 transcripts were analyzed by MiSeq™ amplicon sequencing as described in Fig. 4. (**A**) Dots depict mutation frequencies in each sequence. Pie chart slices depict the proportions of transcripts carrying given numbers of point-mutations; slice gray gradients depict increasing numbers of point-mutations (from 0 to more than 12) per transcript. Numbers below pie charts depict average overall point-mutation frequency (change/base) of all transcripts. Donut charts depict the nature of point-mutations. Histograms depict the overall mutation frequency (change/base) and frequency of point-mutations in V1-72 FRs and CDRs within V1-72DJH-Cγ1 transcripts (mean ± SEM). Each dot represents the mean of approximately 100,000 sequences per mouse. (**B**) Sequences depicting nature and distribution of point-mutations in the V1-72 region of V1-72DJH-Cγ1 transcripts (point-mutations to A, G, C and T are highlighted in green, orange, blue and purple, respectively). CDR1 and CDR2 regions are highlighted in green. Over the 294 bp V1-72 region of V1-72DJH-Cγ1 transcripts, point-mutations were clustered mainly within AID deamination hotspots consisting of the 5'-WRC-3' motif, its reverse complement 5'-GYW-3' (W=A or T, R=A or G and Y=T or C) and the composite 5'-WGCW-3' motif on either DNA strand (identified with nucleotides 38-40, 46-48, 56-59, 91-94, 97-99, 197-199, 221-223 and 289-291 in V1-72DJH-Cγ1). The characteristic CDR1 Trp33Leu (W33L) NP affinity-enhancing replacement, along with the CDR1 Met34Ile (M34I), CDR2 Lys59Arg (K59R) and Ser66Asn (S66N) replacements are identified with nucleotides 97-99, 100-102, 175-177 and 196-198, respectively.

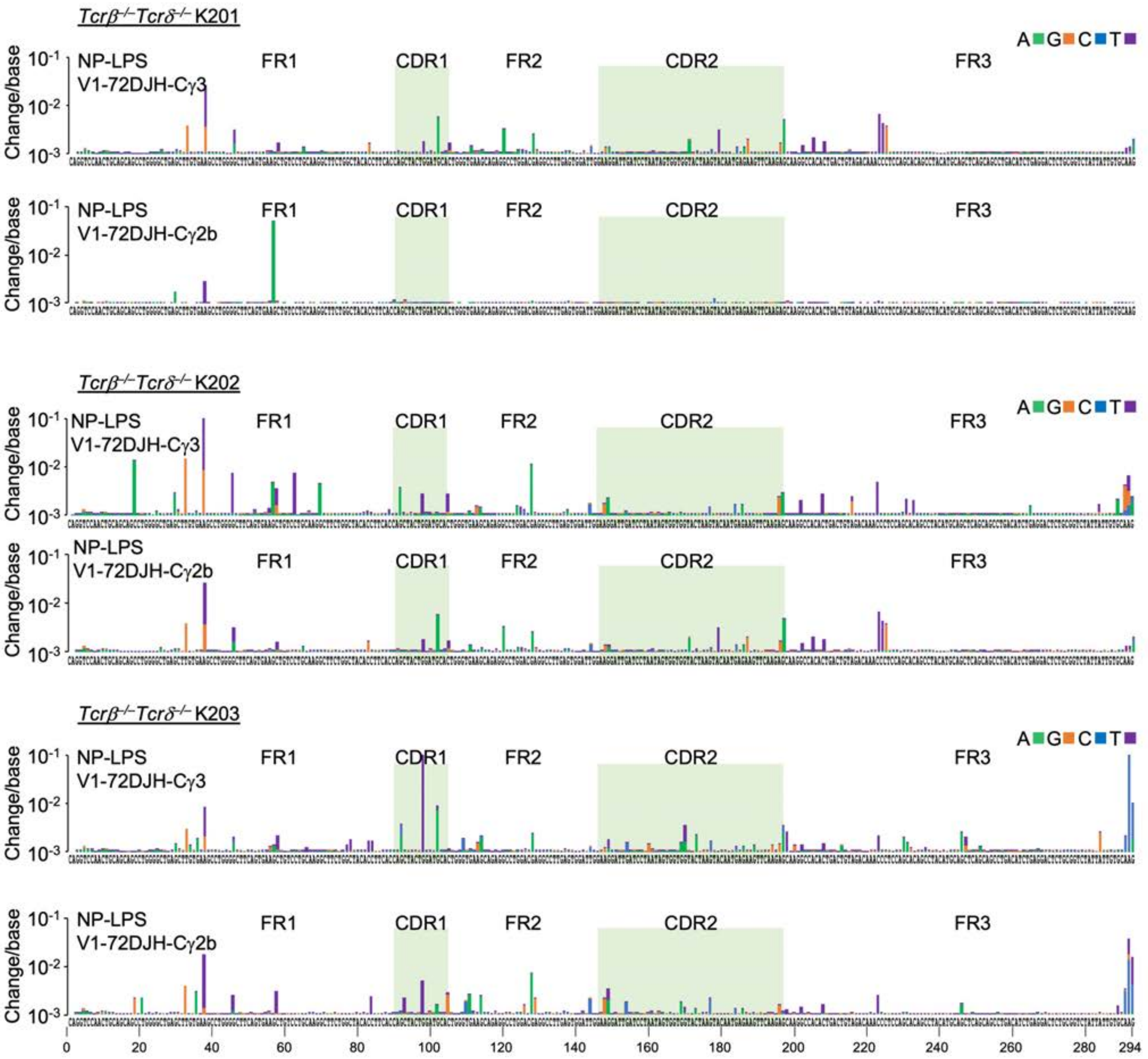

Fig. S9. Distribution and nature of point-mutations in IgH chain V1-72DJ$_H$-Cγ3 and V1-72DJ$_H$-Cγ2b transcripts of NP-specific antibodies induced by B cell TLR4-BCR linked coengagement in *Tcrβ*$^{–/–}$*Tcrδ*$^{–/–}$ mice injected with NP-LPS. Sequences from NP-LPS-injected *Tcrβ*$^{-/-}$*Tcrδ*$^{-/-}$ mice (n = 3 mice, same mice as in Fig. 4: K201, K202 and K203) depict the nature and distribution of point-mutations in the V1-72 region of V1-72DJ$_H$-Cγ3 and V1-72DJ$_H$-Cγ2b transcripts (point-mutations to A, G, C and T are highlighted in green, orange, blue and purple, respectively). CDR1 and CDR2 regions are highlighted in green. Point-mutations were clustered mainly within AID deamination hotspots over the 294 bp V1-72 region of V1-72DJ$_H$-Cγ3 and V1-72DJ$_H$-Cγ2b transcripts. Hotspots consist of the 5'-WRC-3' motif, its reverse complement 5'-GYW-3' (W=A or T, R=A or G and Y=T or C) and the composite 5'-WGCW-3' motif on either DNA strand (identified with nucleotides 38-40, 46-48, 56-59, 91-94, 97-99, 197-199, 221-223 and 289-291 in V1-72DJ$_H$-Cγ3 and V1-72DJ$_H$-Cγ2b). The characteristic CDR1 Trp33Leu (W33L) NP affinity-enhancing replacement, along with the CDR1 Met34Ile (M34I), CDR2 Lys59Arg (K59R) and Ser66Asn (S66N) replacements are identified with nucleotides 97-99, 100-102, 175-177 and 196-198, respectively.

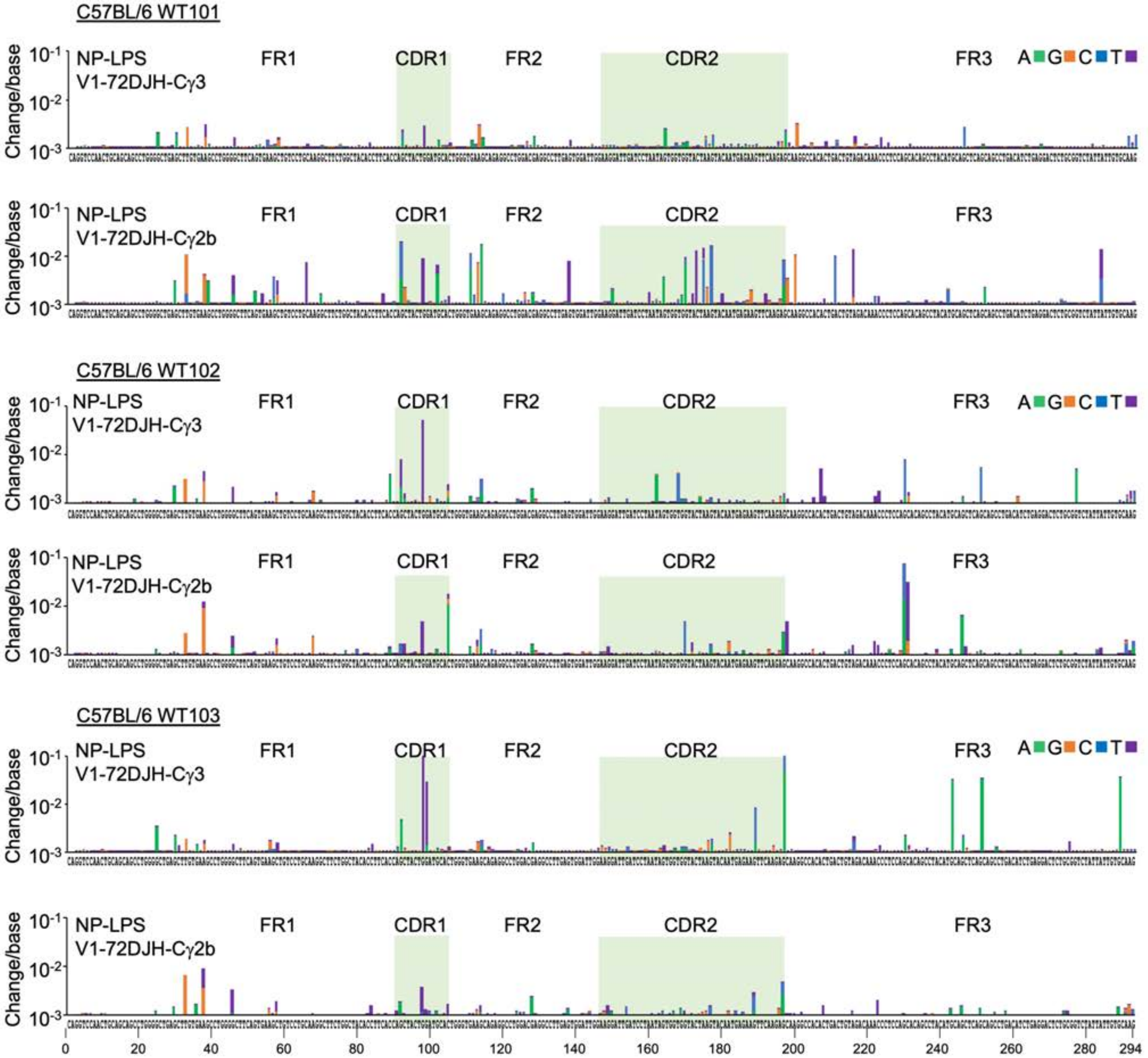

Fig. S10. Distribution and nature of point-mutations in IgH chain V1-72DJ$_H$-Cγ3 and V1-72DJ$_H$-Cγ2b transcripts of NP-specific antibodies induced by B cell TLR4-BCR linked coengagement in C57BL/6 mice injected with NP-LPS. Sequences from NP-LPS-injected C57BL/6 mice (n = 3 mice, same mice as in Fig. 4: WT101, WT102 and WT103) depict the nature and distribution of point-mutations in the V1-72 region of V1-72DJ$_H$-Cγ3 and V1-72DJ$_H$-Cγ2b transcripts (point-mutations to A, G, C and T are highlighted in green, orange, blue and purple, respectively). CDR1 and CDR2 regions are highlighted in green. AID deamination hotspots and NP affinity-enhancing replacements were identified as described in Fig. S9.

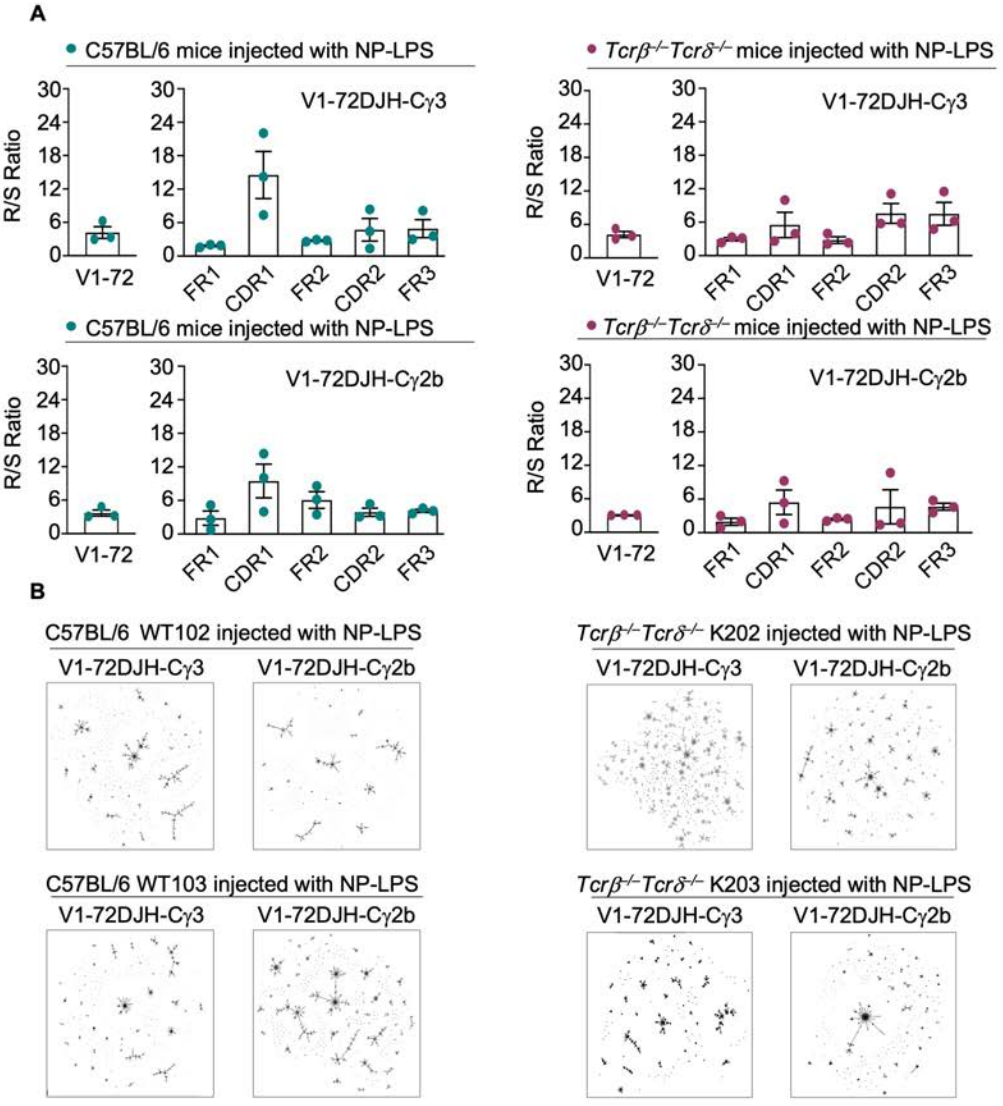

Fig. S11. B cell clonality and R:S point-mutation ratios in IgH V1-72DJ$_H$-Cγ3 and V1-72DJ$_H$-Cγ2b transcripts of NP-specific antibodies induced by B cell TLR4-BCR linked coengagement in C57BL/6 and *Tcrβ*$^{-/-}$*Tcrδ*$^{-/-}$ mice injected with NP-LPS. (**A**) Ratio of replacement (R) to silent (S) point-mutations in the V1-72 region of V1-72DJ$_H$-Cγ3 and V1-72DJ$_H$-Cγ2b transcripts in NP-LPS-immunized C57BL/6 and *Tcrβ*$^{-/-}$*Tcrδ*$^{-/-}$ mice (n = 3 mice per group, same mice as in Fig. 4). Histograms depict mean R:S ratios ± SEM in V1-72 FRs and CDRs. Each dot represents an individual mouse. (**B**) Scatter tree plots depict a partial window of the multitude of clonal genealogical trees for V1-72DJ$_H$-Cγ3 and V1-72DJ$_H$-Cγ2b transcripts in C57BL/6 and *Tcrβ*$^{-/-}$*Tcrδ*$^{-/-}$ mice (same mice as in Fig. 5). The scatter tree plots depicted here may or may not include the trees from the three dominant clones in Fig. 5.

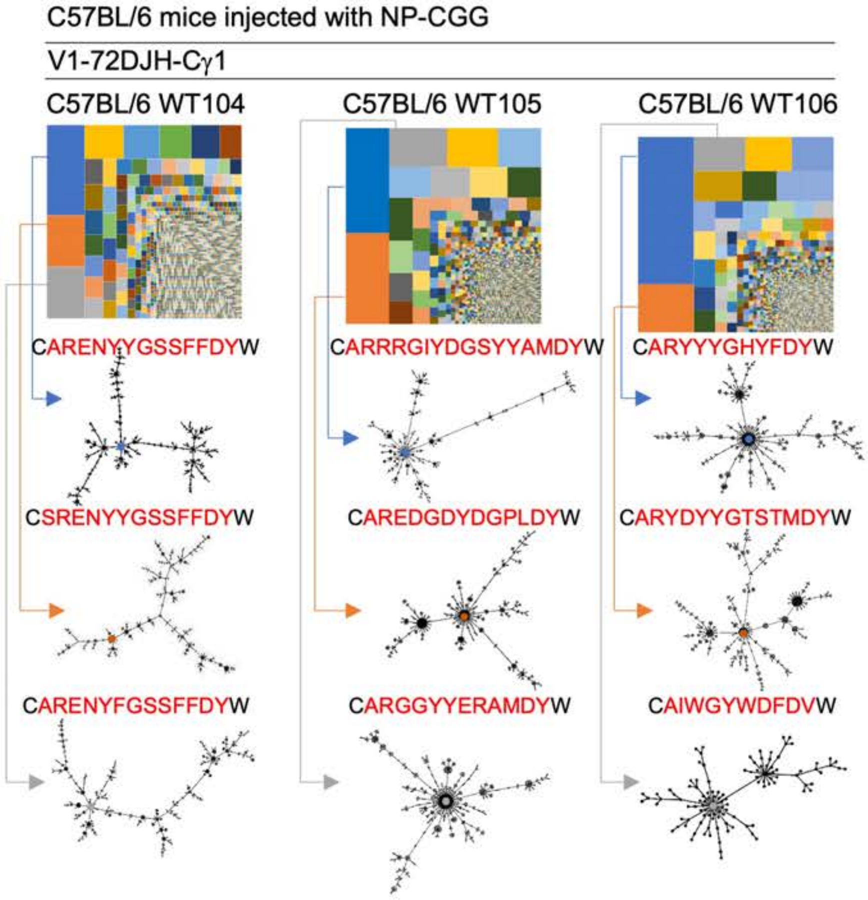

Fig. S12. Clonal B cell expansion and intraclonal diversification in the class-switched anti-NP antibody response induced by NP-CGG in C57BL/6 mice. B cell clonality of the anti-NP IgG1 antibody response in C57BL/6 mice (n = 3 mice, same mice as in Fig. S8: WT104, WT105 and WT106) injected i.p. with NP-CGG on days 0 and 21, as analyzed 14 days after the second injection (day 35). Each B cell clone was identified based on segregation of V1-72DJ$_H$-Cγ1 transcripts consisting of the V1-72 gene segment, the same and unique IgH CDR3 (depicted as translated AA sequence) together with the same J$_H$ sequence. Each discrete clone was depicted as an individual rectangle or square of a unique color, whose area reflected the B cell clone size. Intraclonal diversification is depicted for each of the three dominant clones as a genealogical tree (phylogenic map) constructed based on shared and unique V1-72DJ$_H$-Cγ1 point-mutations. Trees reveal SHM-mediated complex intraconal diversification from unmutated progenitors (color-coded according to respective dominant clones).

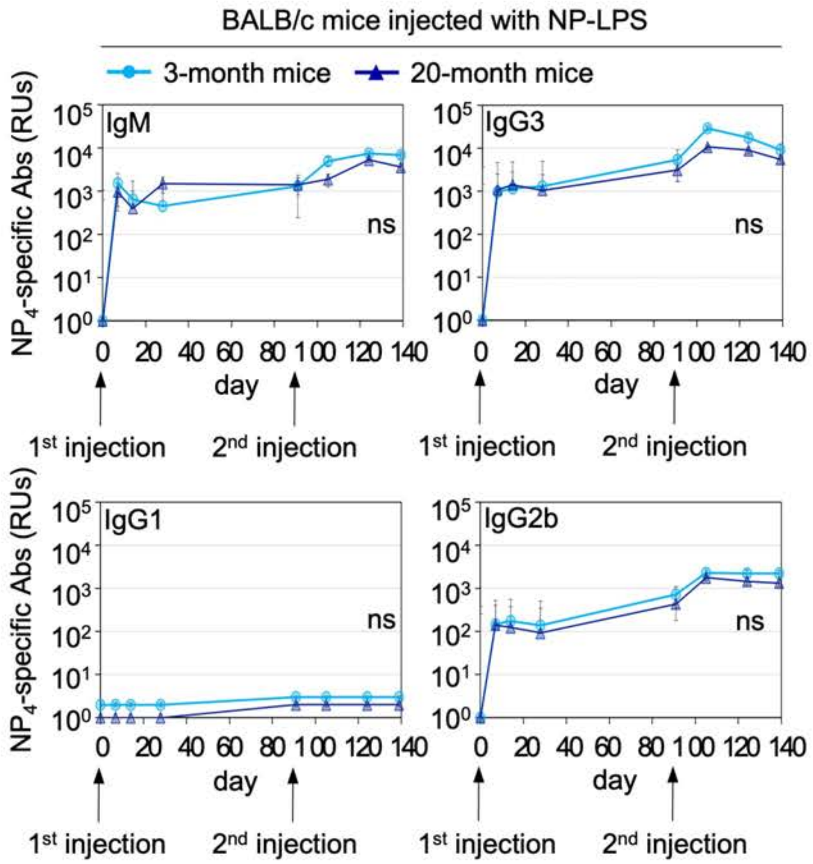

Fig. S13. NP-LPS-induced B cell TLR4-BCR linked coengagement generates a prompt and long-lasting class-switched anamnestic antibody response to NP in young adult and old mice. Three-month-old and 20-month-old BALB/c mice were injected i.p. with NP-LPS on days 0 (25 µg in 100 µl PBS) and 90 (10 µg in 100 µl PBS) (n = 3 mice per group). Sera were collected on days 0, 7, 14, 28, 91, 105, 126 and 140 and analyzed for NP_4-specific IgM, IgG3, IgG1 and IgG2b by ELISA (titers expressed as RUs). The NP-LPS-induced and TLR4-BCR linked coengagement-mediated T-independent class-switched anamnestic antibody response was promptly elicited as late as 90 days after the primary injection and lasted unabated for at least 50 days after the recall injection in both young and old BALB/c mice. Data points represent the mean ± SEM of 3 mice at each time point. Not significant (ns) (unpaired *t*-test).

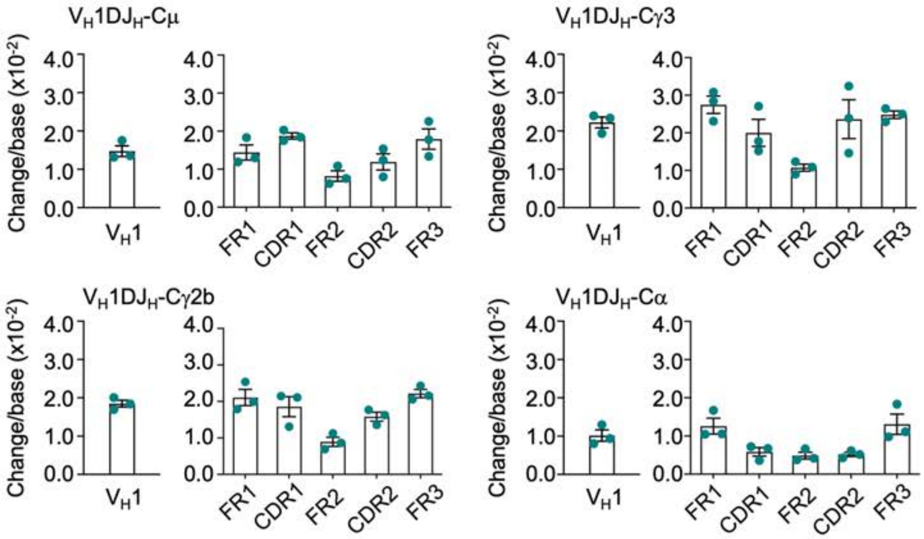

Fig. S14. Frequency of point-mutations in FR1, CDR1, FR2, CDR2 and FR3 of IgH V_H1DJ_H-Cμ, V_H1DJ_H-Cγ3, V_H1DJ_H-Cγ2b and V_H1DJ_H-Cα transcripts induced by B cell TLR5-BCR linked coengagement in *Tcrβ*$^{-/-}$*Tcrδ*$^{-/-}$ mice injected with *S*. Typhimurium flagellin. Sequence analysis of V_H1DJ_H-Cμ, V_H1DJ_H-Cγ3, V_H1DJ_H-Cγ2b and V_H1DJ_H-Cα transcripts in flagellin-injected *Tcrβ*$^{-/-}$*Tcrδ*$^{-/-}$ mice allowed for identification of point-mutations (n = 3 mice, same mice as in Fig. 7: K204, K205 and K206). Histograms depict the overall mutation frequency (change/base) and frequency of point-mutations in V_H1 FRs and CDRs within all transcripts (mean ± SEM). Each dot represents the mean of approximately 10,000 sequences per mouse.

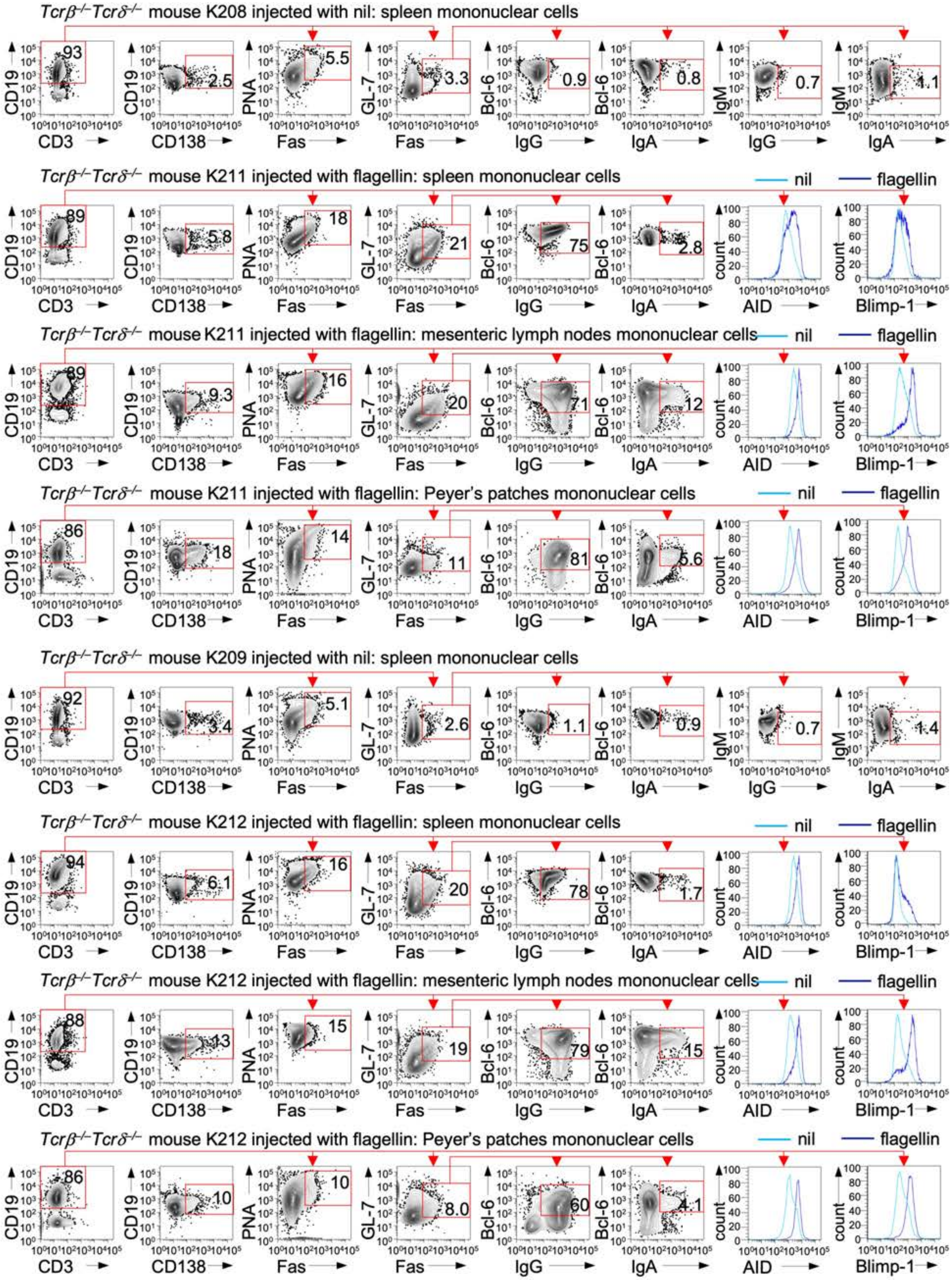
Tcrβ-/-Tcrδ-/- mouse K208 injected with nil: spleen mononuclear cells
93
2.5
5.5
3.3
0.9
0.8
0.7
1.1
Tcrβ-/-Tcrδ-/- mouse K211 injected with flagellin: spleen mononuclear cells
89
5.8
18
21
75
2.8
Tcrβ-/-Tcrδ-/- mouse K211 injected with flagellin: mesenteric lymph nodes mononuclear cells
89
9.3
16
20
71
12
Tcrβ-/-Tcrδ-/- mouse K211 injected with flagellin: Peyer's patches mononuclear cells
86
18
14
11
81
5.6
Tcrβ-/-Tcrδ-/- mouse K209 injected with nil: spleen mononuclear cells
92
3.4
5.1
2.6
1.1
0.9
0.7
1.4
Tcrβ-/-Tcrδ-/- mouse K212 injected with flagellin: spleen mononuclear cells
94
6.1
16
20
78
1.7
Tcrβ-/-Tcrδ-/- mouse K212 injected with flagellin: mesenteric lymph nodes mononuclear cells
88
13
15
19
79
15
Tcrβ-/-Tcrδ-/- mouse K212 injected with flagellin: Peyer's patches mononuclear cells
86
10
10
8.0
60
4.1
nil
flagellin
CD19
CD3
CD138
PNA
Fas
GL-7
Bcl-6
IgG
IgA
IgM
count
AID
Blimp-1

Fig. S15. *S*. Typhimurium TLR5-BCR linked coengagement flagellin-mediated antibody response entails GC-like B cell and plasma cell differentiation in *Tcrβ*$^{-/-}$*Tcrδ*$^{-/-}$ mice. *Tcrβ*$^{-/-}$*Tcrδ*$^{-/-}$ mice were injected i.p. with nil (100 μl alum) (n = 2 mice: K208 and K209) or flagellin (50 μg in 100 μl alum) (n = 2 mice: K211 and K212) on day 0 and again at day 7 with PBS or flagellin in PBS, respectively. Mice were euthanized 7 days after the second injection (day 14). Spleen, mesenteric lymph nodes and Peyer's patches mononuclear cells were analyzed by FACS for PNA$^+$ Fas$^+$, GL7$^+$Fas$^+$ GC-like B cells, as identified by staining with PNA and anti-CD19, anti-GL7, anti-CD95 (Fas), anti-Bcl6 (intracellular staining), anti-IgM, anti-IgG and anti-IgA mAbs. Plasmablasts were identified by surface staining with anti-CD19 and anti-CD138 mAbs. AID and Blimp1 expression were analyzed in B cells of spleens, MLNs and Peyer's patches in flagellin-injected and nil-injected mice by intracellular staining with anti-AID and anti-Blimp1 mAbs followed by flow cytometry analysis. Numbers are percentages of mononuclear cells, as indicated.

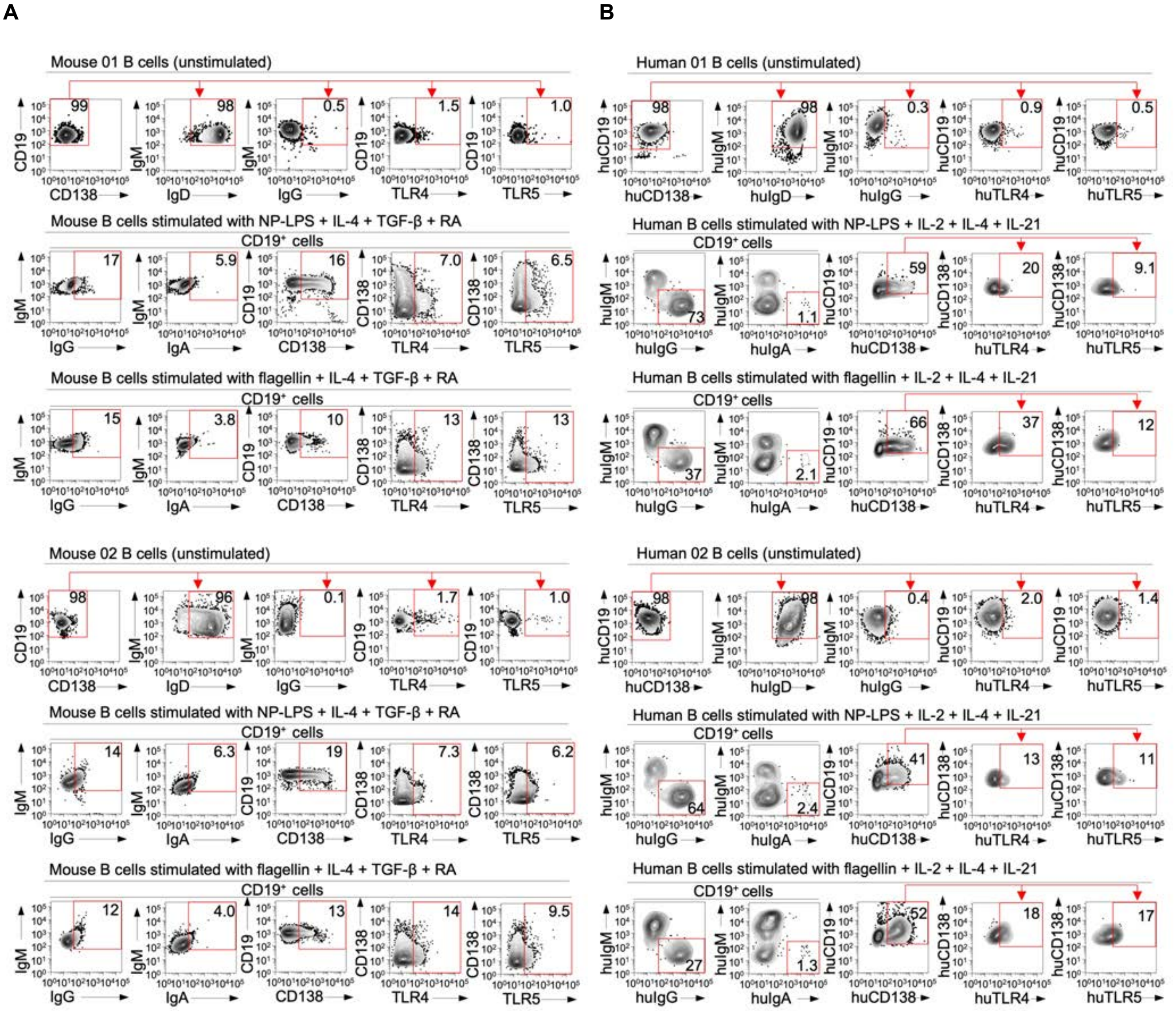

Fig. S16. TLR4-BCR/TLR5-BCR linked coengagement induces TLR4 and TLR5 expression together with CSR and plasma cell differentiation in mouse and human B cells. (**A**) Freshly isolated spleen naïve IgM^+IgD^+ B cells from 2 C57BL/6 mice (01 and 02) were stimulated with nil (unstimulated), NP-LPS (3 μg/ml) plus IL-4 (4 ng/ml), TGF-β (4 ng/ml) and retinoic acid (RA) (4 ng/ml) or *S.* Typhimurium flagellin (10 μg/ml) plus IL-4, TGF-β and RA. After 96 hours, $CD19^+$ B cells were characterized for surface IgM, IgD, IgG, IgA, TLR4 and TLR5 by specific anti-mouse mAbs and plasma cell differentiation by anti-mouse CD138 mAb. Freshly isolated naïve B cells from the same donor mouse were used as control (nil). (**B**) Freshly isolated naïve IgM^+IgD^+ B cells from 2 healthy humans (01 and 02) were stimulated with nil (unstimulated), NP-LPS (3 μg/ml) plus huIL-2 (100 ng/ml), huIL-4 (20 ng/ml) and huIL-21 (50 ng/ml) or *S.* Typhimurium flagellin (10 μg/ml) plus huIL-2, huIL-4 and huIL-21. After 96 hours, $CD19^+$ B cells were characterized for surface IgM, IgD, IgG, IgA, TLR4 and TLR5 by specific anti-human mAbs and plasma cell differentiation by anti-human CD138 mAb. Freshly isolated naïve B cells from the same human donor were used as control (nil). Numbers are percentages of total FACS-analyzed mononuclear cells. Data are of cells from 2 C57BL/6 mice and 2 healthy human subjects.

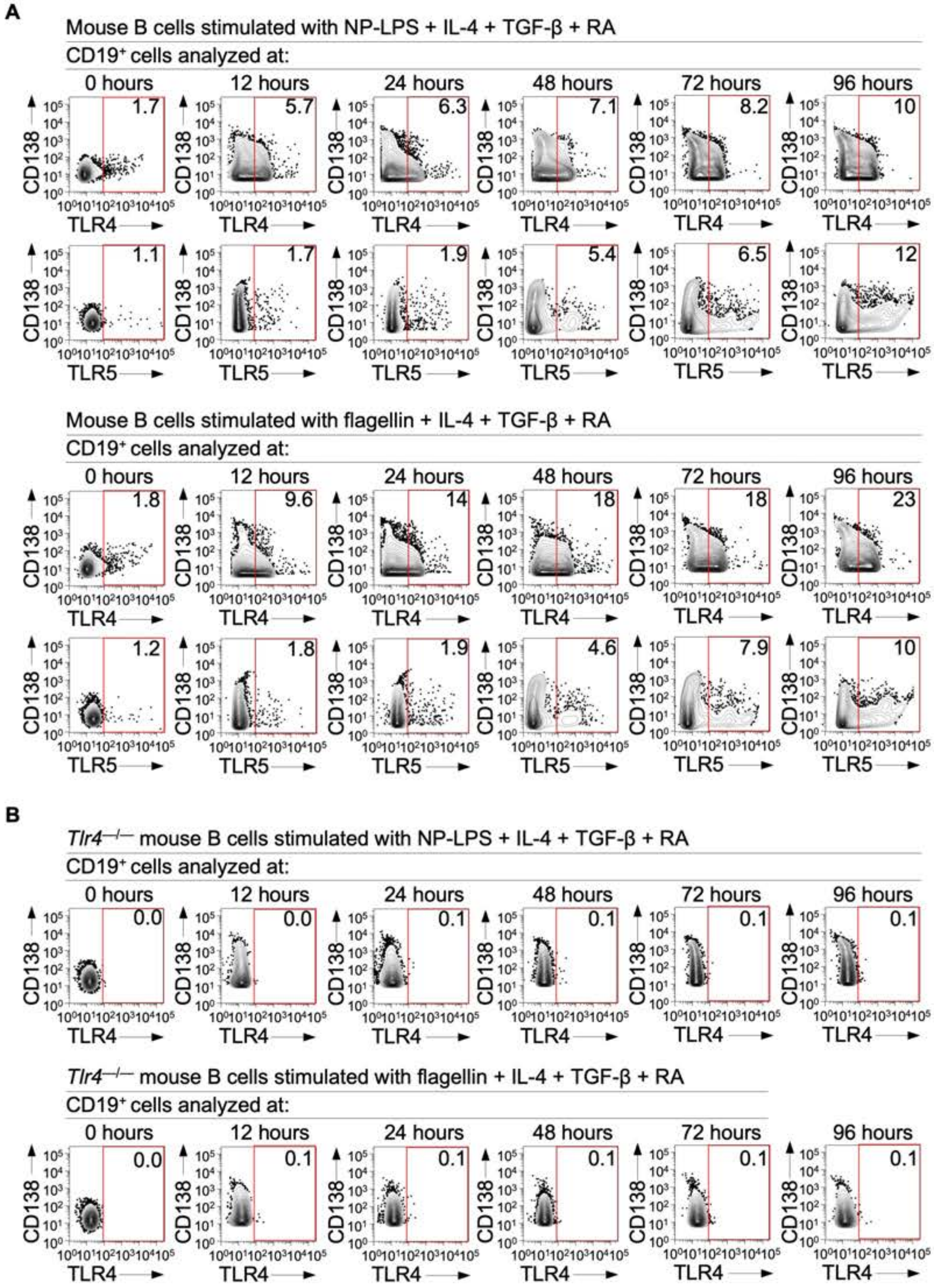

Fig. S17. Kinetics of mouse B cell TLR4 and TLR5 expression concomitant with plasmablast/plasma cell differentiation, as induced by NP-LPS and flagellin. (**A**) Freshly isolated naïve IgM$^+$IgD$^+$ B cells from C57BL/6 mice were stimulated with NP-LPS (3 μg/ml) plus IL-4 (4 ng/ml), TGF-β (4 ng/ml) and retinoic acid (RA) (4 ng/ml) or *S.* Typhimurium flagellin (10 μg/ml) plus IL-4, TGF-β and RA. At 0-, 12-, 24-, 48-, 72- and 96 hours, cultured B cells were analyzed for surface CD19, TLR4, TLR5 and CD138 by FACS using specific anti-mouse mAbs (numbers are percentages). (**B**) Freshly isolated naïve IgM$^+$IgD$^+$ B cells from *Tlr4*$^{-/-}$ mice were stimulated with NP-LPS (3 μg/ml) plus IL-4 (4 ng/ml), TGF-β (4 ng/ml) and retinoic acid (RA) (4 ng/ml) or *S.* Typhimurium flagellin (10 μg/ml) plus IL-4, TGF-β and RA. At 0-, 12-, 24-, 48-, 72- and 96 hours, cultured B cells were analyzed for surface CD19, TLR4 and CD138 by FACS using specific anti-mouse mAbs (numbers are percentages).

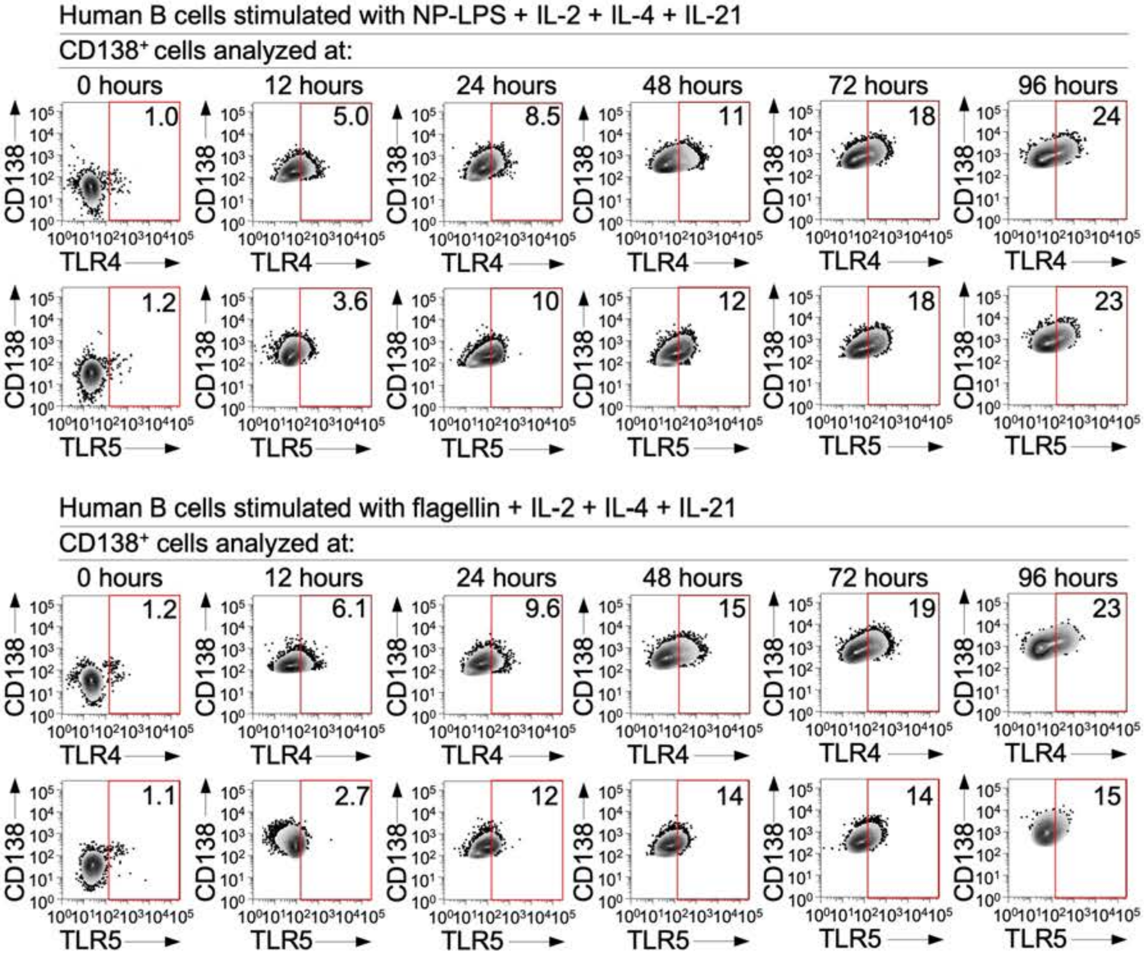

Fig. S18. Kinetics of human B cell TLR4 and TLR5 expression concomitant with plasmablast/plasma cell differentiation, as induced by NP-LPS and flagellin. Freshly isolated naïve IgM⁺IgD⁺ B cells from healthy human PBMCs were stimulated with NP-LPS (3 μg/ml) plus huIL-2 (100 ng/ml), huIL-4 (20 ng/ml) and huIL-21 (50 ng/ml) or *S.* Typhimurium flagellin (10 μg/ml) plus huIL-2, huIL-4 and huIL-21. At 0-, 12-, 24-, 48-, 72- and 96 hours, cultured cells were analyzed for surface CD19, TLR4, TLR5 and CD138 by FACS using specific anti-human mAbs (numbers are percentages). CD138⁺ cells (plasmablasts/plasma cells) were pre-gated and analyzed for TLR4 and TLR5 expression at sequential time-points.

Table S1. CDR3 lengths of V1-72DJ$_H$-Cγ3, V1-72DJ$_H$-Cγ2b and V1-72DJ$_H$-Cγ1 transcripts in C57BL/6 and *Tcrβ*$^{-/-}$*Tcrδ*$^{-/-}$ mice injected with NP-LPS or NP-CGG

	V1-72DJ$_H$-Cγ3 CDR3 length (AA)			V1-72DJ$_H$-Cγ2b CDR3 length (AA)		
C57BL/6 mice	Clone 1	Clone 2	Clone 3	Clone 1	Clone 2	Clone 3
WT101	14	12	13	12	12	16
WT102	12	14	11	14	11	15
WT103	12	13	11	11	15	11
CDR3 AA length	11 to 14			11 to 16		
CDR3 AA length, mean±SD	12.44 ± 1.13			13.00 ± 2.00		

	V1-72DJ$_H$-Cγ3 CDR3 length (AA)			V1-72DJ$_H$-Cγ2b CDR3 length (AA)		
Tcrβ$^{-/-}$*Tcrδ*$^{-/-}$ mice	Clone 1	Clone 2	Clone 3	Clone 1	Clone 2	Clone 3
K201	12	12	8	11	12	12
K202	11	12	11	8	14	12
K203	11	11	11	7	14	13
CDR3 AA length	11 to 12			7 to 14		
CDR3 AA length, mean±SD	11.00 ± 1.23			11.44 ± 2.46		

	V1-72DJ$_H$-Cγ1 CDR3 length (AA)		
C57BL/6 mice	Clone 1	Clone 2	Clone 3
WT104	13	13	13
WT105	16	13	12
WT106	11	13	10
CDR3 AA length	10 to 16		
CDR3 AA length, mean±SD	12.67 ± 1.66		

Table S2. B cell clonality induced by NP-LPS as mediated through TLR4-BCR linked coengagement in C57BL/6 mice.

C57BL/6 WT101				
	V1-72DJ$_H$-Cγ3		V1-72DJ$_H$-Cγ2b	
Clones by size	Number of clones	Number of identical transcripts per clone, mean$\pm$SEM	Number of clones	Number of identical transcripts per clone, mean$\pm$SEM
Dominant clones	3	4721 ± 1311	3	963.7 ± 183.2
Intermediate clones	11	697 ± 155.8	16	522.4 ± 45.06
Small clones	37	182.6 ± 9.592	41	160.5 ± 7.209
Microclones	3422	3.025 ± 0.1378	4878	3.148 ± 0.124
C57BL/6 WT102				
	V1-72DJ$_H$-Cγ3		V1-72DJ$_H$-Cγ2b	
Clones by size	Number of clones	Number of identical transcripts per clone, mean$\pm$SEM	Number of clones	Number of identical transcripts per clone, mean$\pm$SEM
Dominant clones	3	1869 ± 124.4	3	1520 ± 228.9
Intermediate clones	16	539.6 ± 43.41	13	577.2 ± 76.84
Small clones	56	189.1 ± 10.11	45	169.8 ± 8.473
Microclones	5764	3.383 ± 0.118	4789	3.144 ± 0.126
C57BL/6 WT103				
	V1-72DJ$_H$-Cγ3		V1-72DJ$_H$-Cγ2b	
Clones by size	Number of clones	Number of identical transcripts per clone, mean$\pm$SEM	Number of clones	Number of identical transcripts per clone, mean$\pm$SEM
Dominant clones	3	1433 ± 475.7	3	1651 ± 379.2
Intermediate clones	15	481.5 ± 40.85	9	825.7 ± 87.75
Small clones	60	178.2 ± 6.616	40	193.9 ± 13.27
Microclones	4891	3.316 ± 0.121	4029	2.609 ± 0.110

A total of 3473, 5839 and 4969 V1-72DJ$_H$-Cγ3, and 4938, 4850 and 4081 V1-72DJ$_H$-Cγ2b total recombined and discrete V1-72DJ$_H$ clones was identified in the 3 C57BL/6 mice injected with NP-LPS. Clones were categorized as dominant clones (those comprising a mean of 963 to 1869 V1-72DJ$_H$-Cγ3 or V1-72DJ$_H$-Cγ2b transcripts, all identical but differing only by unique mutations), intermediate clones (mean of 201 to 826 transcripts), small clones (mean of 127-194 transcripts) and microclones (mean of 2-5 transcripts). In each of the 3 C57BL/6 mice (WT101, WT102, WT103), the total B cell clones could be segregated into 3 dominant clones (both V1-72DJ$_H$-Cγ3 and V1-72DJ$_H$-Cγ2b), 11 to 16 (V1-72DJ$_H$-Cγ3) and 9 to 16 (V1-72DJ$_H$-Cγ2b) intermediate clones, 37 to 60 (V1-72DJ$_H$-Cγ3) and 40 to 45 (V1-72DJ$_H$-Cγ2b) small clones, and, finally, 3422 to 5764 (V1-72DJ$_H$-Cγ3) and 4029 to 4878 (V1-72DJ$_H$-Cγ2b) microclones.

Table S3. B cell clonality induced by NP-LPS as mediated through TLR4-BCR linked coengagement in *Tcrβ*$^{-/-}$*Tcrδ*$^{-/-}$ mice.

Tcrβ$^{-/-}$*Tcrδ*$^{-/-}$ K201				
	V1-72DJ$_H$-Cγ3		V1-72DJ$_H$-Cγ2b	
Clones by size	Number of clones	Number of identical transcripts per clone, mean±SEM	Number of clones	Number of identical transcripts per clone, mean±SEM
Dominant clones	3	1214 ± 321.1	3	1148 ± 210.0
Intermediate clones	10	431.8 ± 46.26	13	370.9 ± 26.44
Small clones	34	177.2 ± 5.633	26	193.3 ± 13.83
Microclones	2212	2.174 ± 0.117	3874	3.031 ± 0.127
Tcrβ$^{-/-}$*Tcrδ*$^{-/-}$ K202				
	V1-72DJ$_H$-Cγ3		V1-72DJ$_H$-Cγ2b	
Clones by size	Number of clones	Number of identical transcripts per clone, mean±SEM	Number of clones	Number of identical transcripts per clone, mean±SEM
Dominant clones	3	1284 ± 252.5	3	989.3 ± 315.9
Intermediate clones	11	440.8 ± 48.95	10	353.4 ± 22.81
Small clones	32	143.9 ± 7.616	12	170.3 ± 8.179
Microclones	3057	2.803 ± 0.166	1817	3.124 ± 0.216
Tcrβ$^{-/-}$*Tcrδ*$^{-/-}$ K203				
	V1-72DJ$_H$-Cγ3		V1-72DJ$_H$-Cγ2b	
Clones by size	Number of clones	Number of identical transcripts per clone, mean±SEM	Number of clones	Number of identical transcripts per clone, mean±SEM
Dominant clones	2	1142 ± 574.5	2	1058 ± 706.5
Intermediate clones	10	292.7 ± 32.72	11	201.1 ± 15.07
Small clones	17	127.7 ± 6.764	13	127.8 ± 3.575
Microclones	1568	4.013 ± 0.283	1636	3.429 ± 0.239

A total of 2259, 3103 and 1597 V1-72DJ$_H$-Cγ3, and 3916, 1842 and 1662 V1-72DJ$_H$-Cγ2b total recombined and discrete V1-72DJ$_H$ clones was identified in the 3 *Tcrβ*$^{-/-}$*Tcrδ*$^{-/-}$ mice injected with NP-LPS. Clones were categorized as dominant clones (those comprising a mean of 963 to 1869 V1-72DJ$_H$-Cγ3 or V1-72DJ$_H$-Cγ2b transcripts, all identical but differing only by unique mutations), intermediate clones (mean of 201-826 transcripts), small clones (mean of 127-194 transcripts) and microclones (mean of 2-5 transcripts). In the K201 and K202 *Tcrβ*$^{-/-}$*Tcrδ*$^{-/-}$ mice, the total B cell clones could be segregated into 3 dominant clones (both V1-72DJ$_H$-Cγ3 and V1-72DJ$_H$-Cγ2b), 10 to 11 (V1-72DJ$_H$-Cγ3) and 10 to 13 (V1-72DJ$_H$-Cγ2b) intermediate clones, 17 to 34 (V1-72DJ$_H$-Cγ3) and 12 to 26 (V1-72DJ$_H$-Cγ2b) small clones, and, finally, 1568 to 3057 (V1-72DJ$_H$-Cγ3) and 1636 to 3874 (V1-72DJ$_H$-Cγ2b) microclones. The total B cell clones in the other *Tcrβ*$^{-/-}$*Tcrδ*$^{-/-}$ mouse (K203) were segregated into 2 dominant clones (both V1-72DJ$_H$-Cγ3 and V1-72DJ$_H$-Cγ2b), 10 (V1-72DJ$_H$-Cγ3) and 11 (V1-72DJ$_H$-Cγ2b) intermediate clones, 17 (V1-72DJ$_H$-Cγ3) and 13 (V1-72DJ$_H$-Cγ2b) small clones and 1568 (V1-72DJ$_H$-Cγ3) and 1636 (V1-72DJ$_H$-Cγ2b) microclones.

Table S4. B cell clonality induced by NP-CGG in C57BL/6 mice.

V1-72DJ$_H$-Cγ1 transcripts						
	C57BL/6 WT104		C57BL/6 WT105		C57BL/6 WT106	
Clones by size	Number of clones	Number of identical transcripts per clone, mean±SEM	Number of clones	Number of identical transcripts per clone, mean±SEM	Number of clones	Number of identical transcripts per clone, mean±SEM
Dominant Clones	3	847.3 ± 167.8	2	1971 ± 486.5	2	3090 ± 1693
Intermediate Clones	17	202.8 ± 28.92	8	747.0 ± 168.6	16	335.6 ± 78.50
Small Clones	110	30.01 ± 1.996	98	23.41 ± 1.615	86	25.38 ± 1.952
Microclones	5764	1.152 ± 0.010	4789	1.207 ± 0.013	1656	1.406 ± 0.031

A total of 5894, 4897 and 1760 V1-72DJ$_H$-Cγ1 total recombined and discrete V1-72DJ$_H$ clones was identified in the 3 C57BL/6 mice injected with NP-CGG. Clones were categorized as dominant clones (those comprising a mean of 847-3090 V1-72DJ$_H$-Cγ1 transcripts, all identical but differing only by unique mutations), intermediate clones (mean of 202-336 transcripts), small clones (mean of 23-30 transcripts) and microclones (mean of 1-2 transcripts). The total B cell clones could be segregated into 3 dominant clones, 8 to 17 intermediate clones, 86 to 110 small clones, and, finally, 1656 to 5764 microclones.

**Table S5**. CDR3 lengths of V_H1DJ_H-Cγ3, V_H1DJ_H-Cγ2b and V_H1DJ_H-Cα transcripts in *Tcrβ*$^{-/-}$*Tcrδ*$^{-/-}$ mice injected with *S.* Typhimurium flagellin.									
	V_H1DJ_H-Cγ3 CDR3 length (AA)			V_H1DJ_H-Cγ2b CDR3 length (AA)			V_H1DJ_H-Cα CDR3 length (AA)		
Tcrβ$^{-/-}$ *Tcrδ*$^{-/-}$ mouse	Clone 1	Clone 2	Clone 3	Clone 1	Clone 2	Clone 3	Clone 1	Clone 2	Clone 3
K204	12	9	9	9	12	10	15	6	6
K205	12	10	14	11	12	11	11	10	10
K206	10	10	11	11	8	13	11	15	10
CDR3 AA length	9 to 14			8 to 13			6 to 15		
CDR3 AA length, mean±SD	10.78 ± 1.64			10.78 ± 1.56			10.44 ± 3.21		

Table S6. B cell clonality induced by flagellin as mediated through TLR5-BCR linked coengagement in *Tcrβ*$^{-/-}$*Tcrδ*$^{-/-}$ mice.

Tcrβ$^{-/-}$*Tcrδ*$^{-/-}$ K204						
	V_H1DJ_H-Cγ3		V_H1DJ_H-Cγ2b		V_H1DJ_H-Cα	
Clones by size	Number of clones	Number of identical transcripts per clone, mean±SEM	Number of clones	Number of identical transcripts per clone, mean±SEM	Number of clones	Number of identical transcripts per clone, mean±SEM
Dominant clones	3	266.3 ± 44.52	3	49.33 ± 2.028	2	570.5 ± 101.5
Intermediate clones	13	81.54 ± 10.55	8	29.38 ± 2.251	24	63.04 ± 9.155
Small clones	79	13.18 ± 0.766	27	9.926 ± 0.846	34	12.41 ± 1.193
Microclones	788	1.438 ± 0.031	522	1.446 ± 0.041	381	1.325 ± 0.042
Tcrβ$^{-/-}$*Tcrδ*$^{-/-}$ K205						
	V_H1DJ_H-Cγ3		V_H1DJ_H-Cγ2b		V_H1DJ_H-Cα	
Clones by size	Number of clones	Number of identical transcripts per clone, mean±SEM	Number of clones	Number of identical transcripts per clone, mean±SEM	Number of clones	Number of identical transcripts per clone, mean±SEM
Dominant clones	2	410.0 ± 62.00	2	116.5 ± 18.50	2	334.5 ± 62.50
Intermediate clones	39	66.44 ± 6.602	9	27.78 ± 1.847	17	80.24 ± 13.77
Small clones	100	12.77 ± 0.675	36	10.92 ± 40.6870	20	16.45 ± 1.704
Microclones	576	1.422 ± 0.038	303	1.455 ± 0.055	332	1.319 ± 0.043
Tcrβ$^{-/-}$*Tcrδ*$^{-/-}$ K206						
	V_H1DJ_H-Cγ3		V_H1DJ_H-Cγ2b		V_H1DJ_H-Cα	
Clones by size	Number of clones	Number of identical transcripts per clone, mean±SEM	Number of clones	Number of identical transcripts per clone, mean±SEM	Number of clones	Number of identical transcripts per clone, mean±SEM
Dominant clones	4	630.5 ± 199.0	3	75.67 ± 9.821	1	1239 ± 0.000
Intermediate clones	72	56.14 ± 3.980	8	28.38 ± 2.390	17	116.3 ± 24.31
Small clones	366	12.71 ± 0.353	63	9.365 ± 0.431	41	12.46 ± 1.198
Microclones	2866	1.534 ± 0.019	1072	1.398 ± 0.027	442	1.364 ± 0.039

A total of 717, 883 and 3308 V_H1DJ_H-Cγ3, 350, 560 and 1146 V_H1DJ_H-Cγ2b and 371, 441 and 502 V_H1DJ_H-Cα clones was identified in the 3 *Tcrβ*$^{-/-}$*Tcrδ*$^{-/-}$ mice injected with flagellin. Clones were categorized as dominant clones (those comprising 116 to 1239 V_H1DJ_H-Cγ3, V_H1DJ_H-Cγ2b or V_H1DJ_H-Cα transcripts, all identical but differing only by unique mutations), intermediate clones (mean of 28 to 117 transcripts), small clones (mean of 9 to 17 transcripts) and microclones (mean of 1 to 2 transcripts). The total B cell clones could be segregated into 2 to 4, 2 to 3 and 2 to 4 (V_H1DJ_H-Cγ3, V_H1DJ_H-Cγ2b and V_H1DJ_H-Cα) dominant clones, 13 to 72 , 8 to 9 and 15 to 24 (V_H1DJ_H-Cγ3, V_H1DJ_H-Cγ2b and V_H1DJ_H-Cα) intermediate clones, 79 to 366, 27 to 63 and 20 to 41 (V_H1DJ_H-Cγ3, V_H1DJ_H-Cγ2b and V_H1DJ_H-Cα) small clones, and 576 to 2866, 303 to 1072 and 332 to 442 (V_H1DJ_H-Cγ3, V_H1DJ_H-Cγ2b and V_H1DJ_H-Cα) microclones.

**Table S7**. TLR4 and TLR5 fold expression in naïve and TL4-BCR/TLR5-BCR linked coengagement-induced B cells.				
	TLR4		TLR5	
**Mouse B cells stimulated with:**	Pixel density	Fold increase	Pixel density	Fold increase
Nil	3468.811	1	2647.57	1
NP-LPS + IL-4	12116.125	3.49287551	15996.7	6.04204
Flagellin + IL-4	22502.522	6.48709947	18973.7	7.16647
Nil	2177.669	1	3580.15	1
NP-LPS + TGF-β + RA	14621.246	6.71417282	13887	3.87888
Flagellin + TGF-β + RA	23879.924	10.965819	25842.7	7.21831
	TLR4		TLR5	
**Human B cells stimulated with:**	Pixel density	Fold increase	Pixel density	Fold increase
Nil	3010.397	1	5653.76	1
NP-LPS + IL-2 + IL4 + IL-21	16947.246	5.62957178	18904.9	3.34377
Flagellin + IL-2 + IL-4 + IL-21	18052.903	5.99685125	27253.3	4.82038
Values were derived from analysis of at least 50 single cell images per group of Fig. 9 by ImageJ Pixel Density analysis.				

**Table S8.** Antibodies and reagents.		
Flow Cytometry		
α–moCD19-Pacific Blue™ mAb	BioLegend	Cat. # 115523 (Clone 6D5)
α–moCD19- PerCP-Cy5.5 mAb	BioLegend	Cat. # 144605 (Clone 1D3)
α–moCD19-APC-Cy7 mAb	BioLegend	Cat. # 115529 (Clone 6D5)
α–moCD19-PE mAb	BioLegend	Cat. # 115508 (Clone 6D5)
α–moCD19-PE-Cy7 mAb	TONBO Biosciences	Cat. # 60-0193 (Clone 1D3)
α–moCD19-APC mAb	TONBO Biosciences	Cat. # 20-0193 (Clone 1D3)
α–moCD19-FITC mAb	TONBO Biosciences	Cat. # 35-0193 (Clone 1D3)
α–moCD138-PE-Cy7 mAb	BioLegend	Cat. # 142513 (Clone 281-2)
α–moCD138-APC mAb	BioLegend	Cat. # 142505 (Clone 281-2)
α–moIgM-APC mAb	BioLegend	Cat. # 406509 (Clone RMM-1)
α–moIgM-FITC mAb	BioLegend	Cat. # 406506 (Clone RMM-1)
α–moIgM-PE mAb	BioLegend	Cat. # 406507 (Clone RMM-1)
α–moIgM-BV605™ mAb	BioLegend	Cat. # 406523 (Clone RMM-1)
α–moIgD-PerCp/Cyanine5.5 mAb	BioLegend	Cat. # 405709 (Clone 11-26c.2a)
α–moIgD-APC mAb	BioLegend	Cat. # 405714 (Clone 11-26c.2a)
α–moIgG-Alexa Flour® 647 pAb	BioLegend	Cat. # 405322 (Poly4053)
α–moIgG-FITC pAb	BioLegend	Cat. # 406001 (Poly4060)
α–moIgG1-FITC mAb	BioLegend	Cat. # 406606 (Clone RMG1-1)
α–moIgG1-APC mAb	BioLegend	Cat. # 406609 (Clone RMG1-1)
α–moIgG3-FITC mAb	BD Biosciences	Cat. # 553403 (Clone R40-82)
α–moIgG3-BV421™ mAb	BD Biosciences	Cat. # 565808 (Clone R40-82)
α–moIgG2a-APC mAb	BioLegend	Cat. # 407110 (Clone RMG2a-62)
α–moIgG2b-FITC mAb	BioLegend	Cat. # 406705 (Clone RMG2b-1)
α–moIgA-FITC mAb	eBioscience	Cat. # 11-4204-82 (Clone mA-6E1)
α–moIgA-PE mAb	eBioscience	Cat. # 12-4204-81 (Clone mA-6E1)
α–moIgA-APC mAb	eBioscience	Cat. # 17-4204-82 (Clone mA-6E1)
α–moCD3-Pacific Blue™ mAb	BioLegend	Cat. # 100214 (Clone 17A2)
α–moCD3-APC-Cy7 mAb	BioLegend	Cat. # 100221 (Clone 17A2)
α–moCD3-biotin mAb	BioLegend	Cat. # 100243 (Clone 17A2)
α–moCD11b-APC mAb	BioLegend	Cat. # 101212 (Clone M1/70)
α–moCD11b-biotin mAb	BioLegend	Cat. # 101204 (Clone M1/70)
α–moCD11c-APC-Cy7 mAb	BioLegend	Cat. # 117323 (Clone N418)
α–moCD45-Pacific Blue™ mAb	BioLegend	Cat. # 103126 (Clone 30-F11)
α–moCD45-PerCP mAb	BioLegend	Cat. # 103130 (Clone 30-F11)
α–moGL7-FITC mAb	BioLegend	Cat. # 144603 (Clone GL7)
α–moGL7-PE mAb	BioLegend	Cat. # 144607 (Clone GL7)
α–moGL7-PerCP-Cy5.5 mAb	BioLegend	Cat. # 144609 (Clone GL7)
α–moCD95-BV510™ mAb	BD Biosciences	Cat. # 563646 (Clone Jo2)
α–moFoxp3-PE-Cy7 mAb	TONBO Biosciences	Cat. # 60-5773 (Clone 3G3)
α–moIFNγ-BV650™ mAb	BD Biosciences	Cat. # 505832 (Clone XMG 1.2)
α–moIL-4-BV421™ mAb	BD Biosciences	Cat. # 504127 (Clone 11B11)
α–moIL-17A-PE mAb	BioLegend	Cat. # 506904 (Clone TC11-18H10.1)
α–mo/hu-Bcl-6-PE mAb	BioLegend	Cat. # 648303 (Clone IG191E/A8)
α–mo-Blimp-1-PE mAb	BioLegend	Cat. # 150005 (Clone 5E7)
α–mo-AID-Alexa Flour® 647 pAb	Bioss Antibodies	Cat. # BS-7855R-A647
Immunofluorescence staining		
α–moB220-FITC mAb	BioLegend	Cat. # 103206 (Clone RA3-6B2)
α–moB220-PE mAb	TONBO Bioscience	Cat. # 50-0452-U025 (Clone RA3-6B2)
α–moGL7-Alexa Flour® 647 mAb	BioLegend	Cat. # 144605 (Clone GL7)
Lectin PNA-Alexa Flour® 488	ThermoFisher	Cat. # L21409

ELISA		
α–moIgM-UNLB pAb	SouthernBiotech	Cat. # 1020-01
α–moIgG-UNLB pAb	SouthernBiotech	Cat. # 1030-01
α–moIgA-UNLB pAb	SouthernBiotech	Cat. # 1040-01
α–moIgM-biotin pAb	SouthernBiotech	Cat. # 1020-08
α–moIgG1-biotin pAb	SouthernBiotech	Cat. # 1070-08
α–moIgG2a-biotin pAb	SouthernBiotech	Cat. # 1080-08
α–moIgG2b-biotin pAb	SouthernBiotech	Cat. # 1090-08
α–moIgG3-biotin pAb	SouthernBiotech	Cat. # 1100-08
α–moIgA-biotin pAb	SouthernBiotech	Cat. # 1040-08
α–moIgG-biotin pAb	SouthernBiotech	Cat. # 1030-08
Other flow cytometry reagents		
Fixable Viability Dye eFluor™ 780	eBioscience	Cat. # 65-0865-14
Fixable Viability Dye eFluor™ 506	eBioscience	Cat. # 65-0866-14
NP_5-PE	Biosearch Technologies	Cat. # N-5070-1
NP_{23}-PE	Biosearch Technologies	Cat. # N-5070-1

**Table S9.** Anti-human B cell antibodies and reagents.		
Flow Cytometry		
α–huCD19-PE-Cy7 mAb	TONBO Biosciences	Cat. # 60-0199 (Clone MI15)
α–huCD138-BV510™mAb	BioLegend	Cat. # 356518 (Clone B-B4)
α–huCD284 (TLR4)-PE mAb	BioLegend	Cat. # 312805 (Clone HTA125)
α–huCD285 (TLR5)-APC mAb	BioLegend	Cat. # 394507 (Clone S16021I)
α–huCD27-PE mAb	BioLegend	Cat. # 356405 (Clone M-T271)
α–huIgM-BV421™ mAb	BioLegend	Cat. # 314516 (Clone MHM-88)
α–huIgD-PerCp mAb	BioLegend	Cat. # 348233 (Clone IA6-2)
α–huIgG-FITC mAb	BD Biosciences	Cat. # 555786 (Clone G18-145 (RUO))
α–huIgA-APC mAb	Miltenyi Biotec	Cat. # 130-116-879 (Clone REA1014)
Fixable Viability Dye eFluor™ 780	eBioscience	Cat. # 65-0865-14

Table S10. Mouse PCR oligonucleotide primers.

	Forward Primer	Reverse Primer
Mouse genes		
Aicda	5'-AGAAAGTCACGCTGGAGACC-3'	5'-CTCCTCTTCACCACGTAGCA-3'
Prdm1	5'-GCTGCTGGGCTGCCTTTGGA-3'	5'-GGAGAGGAGGCCGTTCCCCA-3'
Gapdh	5'-TTCACCACCATGGAGAAGGC-3'	5'-GGCATGGACTGTGGTCATGA-3'
Cd79b	5'-CCACACTGGTGCTGTCTTCC-3'	5'-GGGCTTCCTTGGAAATTCAG-3'
Mouse germline transcripts		
Iμ-Cμ	5'-ACCTGGGAATGTATGGTTGTGGCTT-3'	5'-GCAGGCAGGGCTAGATATGG-3'
Iγ3-Cγ3	5'-AACTACTGCTACCACCACCACCAG-3'	5'-AGCCAGGGACCAAGGGATAGAC-3'
Iγ1-Cγ1	5'-TCGAGAAGCCTGAGGAATGTG-3'	5'-ATGGAGTTAGTTTGGGCAGCA-3'
Iγ2b-Cγ2b	5'-GATGGGGAGGAGTTGGCAGAT-3'	5'-CGGAGGAACCAGTTGTATC-3'
Iγ2a-Cγ2a	5’-GCTGATGTACCTACCGAGAGA-3’	5’-GCTGGGCCAAGGTGCTCGAGGTT-3’
Mouse post-recombination transcripts		
Iμ-Cγ3	5'-ACCTGGGAATGTATGGTTGTGGCTT-3'	5'-AGCCAGGGACCAAGGGATAGAC-3'
Iμ-Cγ1	5'-ACCTGGGAATGTATGGTTGTGGCTT-3'	5′-ATGGAGTTAGTTTGGGCAGCA-3′
Iμ-Cγ2b	5'-ACCTGGGAATGTATGGTTGTGGCTT-3'	5'-CGGAGGAACCAGTTGTATC-3'
Iμ-Cγ2a	5'-ACCTGGGAATGTATGGTTGTGGCTT-3'	5'-GCTGGGCCAGGTGCTCGAGGTT-3'
Somatic mutations		
V1-72DJ_H-Cγ3	5'-CATGCTCTTCTTGGCAGCAACAGC-3'	5'-ACCAAGGGATAGACAGATGGGG-3'
V1-72DJ_H-Cγ1	5'-CATGCTCTTCTTGGCAGCAACAGC-3'	5'-GTGCACACCGCTGGACAGGGATCC-3'
V1-72DJ_H-Cγ2b	5'-CATGCTCTTCTTGGCAGCAACAGC-3'	5'-CGGAGGAACCAGTTGTATC-3'
V_{H1}DJ_H-Cμ	5’-CCTGTCAGKAAYTGCAGGTGTCC-3'	5’-GAAATGGTGCTGGGCAGGAA-3'
V_{H1}DJ_H-Cγ3	5’-CCTGTCAGKAAYTGCAGGTGTCC-3'	5'-ACCAAGGGATAGACAGATGGGG-3'
V_{H1}DJ_H-Cγ2b	5’-CCTGTCAGKAAYTGCAGGTGTCC-3'	5'-CGGAGGAACCAGTTGTATC-3'
V_{H1}DJ_H-Cα	5’-CCTGTCAGKAAYTGCAGGTGTCC-3'	5’-CTTGACAGAGCTCGTGGGAG-3'

**Table S11.** Human PCR oligonucleotide primers.		
	Forward Primer	Reverse Primer
Human genes		
AICDA	5'-GTCACCTGGTTCACCTCCTG-3'	5'-CTTGCGGTCCTCACAGAAGT-3'
PRDM1	5'-ATCTTGGGGTAAAAGCGGGT-3'	5'-TCCTGCACTACTGGACACAC-3'
TLR4	5'-CAGAACTGCAGGTGCTGGAT-3'	5'-GGGCTAAACTCTGGATGGGG-3'
TLR5	5'-ACGAGGATCATGGAGACCA-3'	5'-CCTGGGTGAGGTTGCAGAAA-3'
B-ACTIN	5'-AGAGCTACGAGCTGCCTGAC-3'	5'-AGCACTGTGTTGGCGTACAG-3'
Human germline transcripts		
Iμ-Cμ	5'-AGCCCCAGCCCTTGTTAATG-3'	5'-CTTTCGCTCCAGGTCACACT-3'
Iγ1-Cγ1	5'-CTCCACCAAGGGCCCATCGGTCTT-3'	5'-AGTAGTCCTTGACCAGGCAGCC-3'
Iγ2-Cγ2	5'-AGTGCTGGCCGTGGGGCCCTCTC-3'	5'-GTGGGCACTCGACACAACATTTGCG-3'
Iγ3-Cγ3	5'-GACCAAGGACAGCAGGTGAGCT-3'	5'-TTGTGTCACCAAGTGGGGTTTTGAGC-3'
Iγ4-Cγ4	5'-TGTCCAGGCCGGCAGCATCACCAG-3'	5'-ATGGGCATGGGGGACCAATTTGGA-3'
Iα-Cα	5'-AAGGTGAACAGSTTTCATGGC-3'	5'-GTGGGAAGTTTCTGGCGGT-3'
Iε-Cε	5'-GACGGGCCACACCATCC-3'	5'-CGGAGGTGGCATTGGAGG-3'
Human post-recombination transcripts		
Iμ-Cγ1	5'-AGCCCCAGCCCTTGTTAATG-3'	5'-AGTAGTCCTTGACCAGGCAGCC-3'
Iμ-Cγ2	5'-AGCCCCAGCCCTTGTTAATG-3'	5'-GTGGGCACTCGACACAACATTTGCG-3'
Iμ-Cγ3	5'-AGCCCCAGCCCTTGTTAATG-3'	5'-TTGTGTCACCAAGTGGGGTTTTGAGC-3'
Iμ-Cγ4	5'-AGCCCCAGCCCTTGTTAATG-3'	5'-ATGGGCATGGGGGACCAATTTGGA-3'
Iμ-Cα	5'-AGCCCCAGCCCTTGTTAATG-3'	5'-GTGGGAAGTTTCTGGCGGT-3'
Iμ-Cε	5'-AGCCCCAGCCCTTGTTAATG-3'	5'-CGGAGGTGGCATTGGAGG-3'
